# Unsupervised machine learning for exploratory data analysis in imaging mass spectrometry

**DOI:** 10.1002/mas.21602

**Published:** 2019-10-11

**Authors:** Nico Verbeeck, Richard M. Caprioli, Raf Van de Plas

**Affiliations:** ^1^ Delft Center for Systems and Control Delft University of Technology ‐ TU Delft Delft The Netherlands; ^2^ Aspect Analytics NV Genk Belgium; ^3^ STADIUS Center for Dynamical Systems, Signal Processing, and Data Analytics, Department of Electrical Engineering (ESAT) KU Leuven Leuven Belgium; ^4^ Mass Spectrometry Research Center Vanderbilt University Nashville TN; ^5^ Department of Biochemistry Vanderbilt University Nashville TN; ^6^ Department of Chemistry Vanderbilt University Nashville TN; ^7^ Department of Pharmacology Vanderbilt University Nashville TN; ^8^ Department of Medicine Vanderbilt University Nashville TN

**Keywords:** unsupervised, machine learning, data analysis, imaging mass spectrometry, MALDI, SIMS, DESI, LAESI, LAICP, matrix factorization, clustering, manifold learning

## Abstract

Imaging mass spectrometry (IMS) is a rapidly advancing molecular imaging modality that can map the spatial distribution of molecules with high chemical specificity. IMS does not require prior tagging of molecular targets and is able to measure a large number of ions concurrently in a single experiment. While this makes it particularly suited for exploratory analysis, the large amount and high‐dimensional nature of data generated by IMS techniques make automated computational analysis indispensable. Research into computational methods for IMS data has touched upon different aspects, including spectral preprocessing, data formats, dimensionality reduction, spatial registration, sample classification, differential analysis between IMS experiments, and data‐driven fusion methods to extract patterns corroborated by both IMS and other imaging modalities. In this work, we review unsupervised machine learning methods for exploratory analysis of IMS data, with particular focus on (a) factorization, (b) clustering, and (c) manifold learning. To provide a view across the various IMS modalities, we have attempted to include examples from a range of approaches including matrix assisted laser desorption/ionization, desorption electrospray ionization, and secondary ion mass spectrometry‐based IMS. This review aims to be an entry point for both (i) analytical chemists and mass spectrometry experts who want to explore computational techniques; and (ii) computer scientists and data mining specialists who want to enter the IMS field. © 2019 *The Authors*. Mass Spectrometry Reviews published by Wiley Periodicals, Inc. Mass SpecRev 00:1–47, 2019.

## I INTRODUCTION

In the area of molecular imaging, imaging mass spectrometry (IMS) (Caprioli et al., 1997; Pacholski & Winograd, 1999; Stoeckli et al., 2001; Vickerman & Briggs, 2001; McDonnell & Heeren, 2007; Vickerman, 2011; Spengler, 2014) is advancing rapidly as a means of mapping the spatial distribution of molecules throughout a sample. Since IMS does not require prior tagging of the molecular target of interest and can measure multiple ions concurrently in a single experiment, it has proven to be particularly suited for exploratory analysis. Consequently, IMS is currently finding application in an expansive set of domains, ranging from the biomedical exploration of organic tissue (Boxer, Kraft, & Weber, 2009; Schwamborn & Caprioli, 2010; Hanrieder et al., 2013; Schöne, Höfler, & Walch, 2013; Wu et al., 2013; Cassat et al., 2018) and the forensic analysis of fingerprints (Wolstenholme et al., 2009; Elsner & Abel, 2014), to the chemical examination of geological samples (Orphan & House, 2009; Senoner & Unger, 2012) and material science‐related studies (McPhail, 2006; Clark et al., 2016). Furthermore, IMS entails many different instrument platforms, ionization techniques, and mass analyzers. This has led to a variety of different IMS modalities, including matrix‐assisted laser desorption/ionization (MALDI), desorption electrospray ionization (DESI), laser ablation electrospray ionization (LAESI), laser ablation inductively coupled plasma (LAICP), and secondary ion mass spectrometry (SIMS)‐based IMS, each with their own advantages and disadvantages.

Traditionally, there has been a lot of focus on solving sample preparation and instrumental challenges. However, as these are being addressed, some of the complexity in IMS has shifted toward the computational analysis of its data and to the extraction of information from the often‐massive amounts of measurements that an IMS experiment can yield. Computational IMS research tends to be heterogeneous and the type of challenges being addressed currently runs the gamut from the spectrum level (e.g., preprocessing (Deininger et al., 2011; Jones et al., 2012a), peak picking (Du, Kibbe, & Lin, 2006; Alexandrov et al., 2010; McDonnell et al., 2010)) to the intraexperiment level (e.g., clustering/segmentation (Alexandrov & Kobarg, 2011), and from the interexperiment level (e.g., differential analysis between IMS experiments (Piantadosi & Smart, 2002; Le Faouder et al., 2011; Verbeeck et al., 2014a; Carreira et al., 2015) to the intertechnology level (e.g., data‐driven fusion between IMS and microscopy (Van de Plas et al., 2015).

Even within the data mining of IMS experiments, there are clear distinctions between supervised and unsupervised machine learning approaches (Bishop, 2006). Supervised methods for IMS analysis will seek to model a specific recognition task. For example, classification approaches applied to MALDI IMS in a digital pathology context can predict tissue classes and tumor labels after having been shown representative example measurements annotated by a pathologist (Lazova et al., 2012; Meding et al., 2012; Hanselmann et al., 2013; Casadonte et al., 2014; Veselkov et al., 2014). This supervised branch also includes any regression approaches, such as data‐driven multimodal image fusion (Van de Plas et al., 2015), which seeks to model ion distributions in terms of variables measured by another imaging technology. Unsupervised approaches to IMS analysis, on the other hand, are not focused on a particular recognition task, but instead seek to discover the underlying structure within an IMS dataset, uncovering trends, correlations, and associations along the spatial and spectral domains. These methods are generally applied to provide a more open‐ended exploratory perspective on the data, without particular spatial areas or ions of interest in mind. The structure they find in the data can be employed for aiding human interpretation, but can also serve to reduce the dimensionality and computational load for subsequent computational analyses. Unsupervised methods include, for example, factorization methods such as principal component analysis (PCA) and nonnegative matrix factorization (NMF) (Lee & Seung, 1999; Jolliffe, 2002; Van de Plas et al., 2007b), but also clustering approaches seeking to delineate underlying groups of spectra or pixels with similar chemical expression (McCombie et al., 2005; Rokach & Maimon, 2005; Alexandrov et al., 2010).

**Figure 1:**
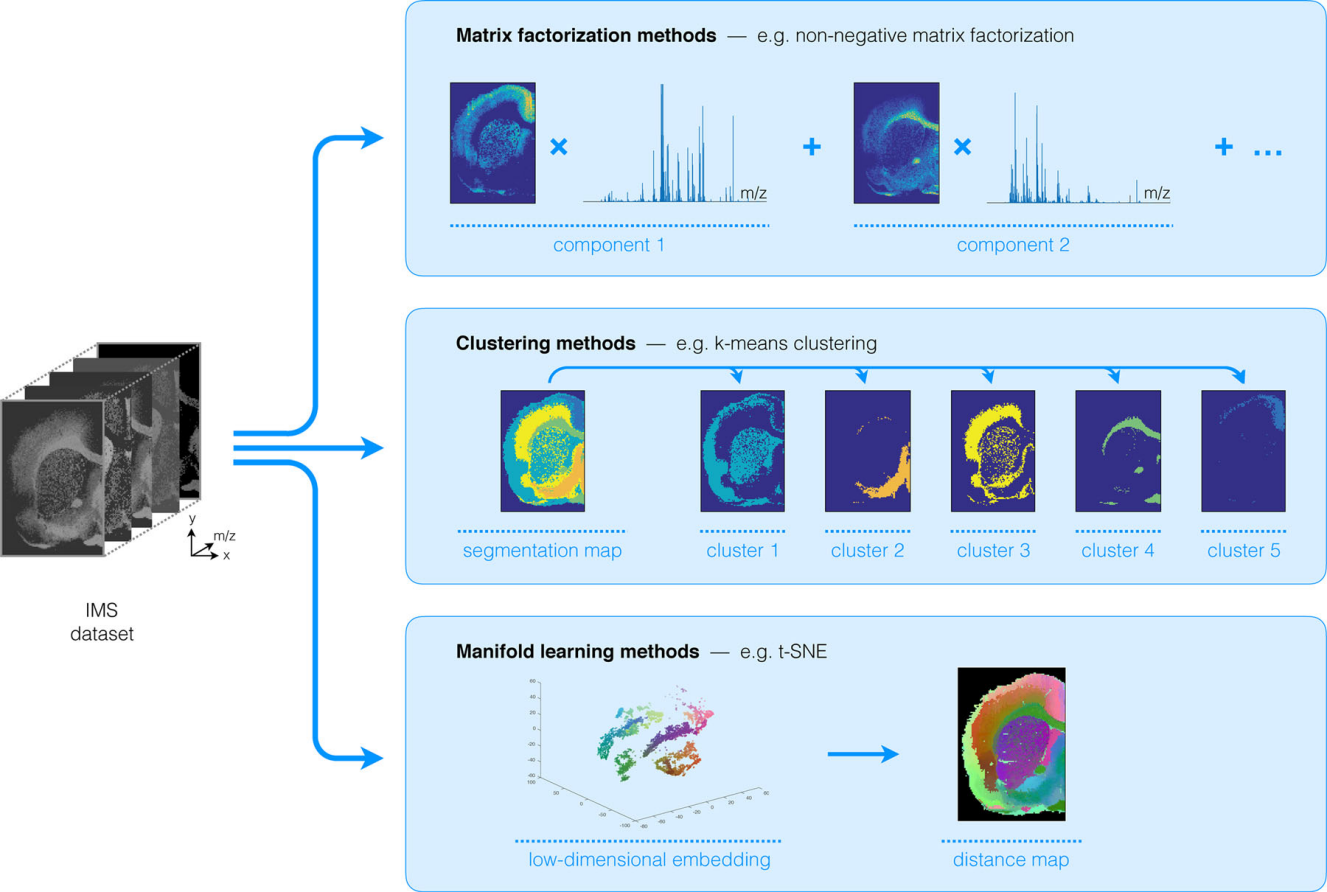
Unsupervised machine learning methods for exploratory data analysis in IMS. An overview of three reviewed method branches, with application to a MALDI FTICR IMS dataset acquired from rat brain (Verbeeck et al., 2017). (Top) Matrix factorization, with nonnegative matrix factorization as a representative example. (Middle) Clustering analysis, with standard *k*‐means clustering as a representative example. (Bottom) Manifold learning, with t‐SNE as a representative example. IMS, imaging mass spectrometry; FTICR, Fourier transform ion cyclotron resonance; MALDI, matrix‐assisted laser desorption/ionization; t‐SNE, t‐distributed stochastic neighborhood embedding. [Color figure can be viewed at wileyonlinelibrary.com]

Providing a comprehensive overview of computational methods in IMS has grown beyond the scope of a single review paper, making any review article necessarily focused on a particular branch of computational analysis. Since we aim to provide a resource for those starting out in IMS data analysis, and since one of the advantages of most forms of IMS is its exploratory potential (due to its multiplexed nature and not requiring prior chemical tagging), this paper will specifically review computational methodology for the exploratory analysis of IMS data. More precisely, this review attempts to collect representative examples of unsupervised machine learning algorithms and their applications in an IMS context. This means that work related to preprocessing (e.g., normalization (Deininger et al., 2011; Fonville et al., 2012; Källback et al., 2012), baseline correction (Coombes et al., 2005; Källback et al., 2012), peak picking and feature detection (McDonnell et al., 2010; Alexandrov et al., 2010; Bedia, Tauler, & Jaumot, 2016; Du, Kibbe, & Lin, 2006), data formats (Schramm et al., 2012; Rübel et al., 2013; Verbeeck et al., 2014a; Verbeeck, 2014b; Verbeeck et al., 2017), spatial registration (Schaaff, McMahon, & Todd, 2002; Abdelmoula et al., 2014a; Anderson et al., 2016; Patterson et al., 2018a, 2018b), and supervised methods such as classification (Luts et al., 2010) and regression (Van de Plas et al., 2015) do not fall within the scope of this review, unless there is a substantial contribution to their analysis pipeline by an unsupervised machine learning algorithm. Our focus will lie on three particular subbranches within unsupervised methods, namely (i) factorization methods, (ii) clustering methods, (iii) manifold learning methods, and any hybrid methods that feature a strong relationship to these approaches. Figure 1 gives an overview of these unsupervised methods, with an application to a MALDI Fourier transform ion cyclotron resonance (FTICR) IMS dataset acquired from rat brain (Verbeeck et al., 2017).

Furthermore, to provide a view across the various instrumental approaches within IMS, we have attempted to include, for each algorithm type, examples from different varieties of IMS. Some analysis methods will show broad application with examples in, for example, MALDI, SIMS, and DESI‐based IMS, while the development of other methods in an IMS context seems to have been confined to a particular instrumental branch. The latter highlights potential for further bridging of computational approaches between the various IMS modalities.

Overall, we have tried to be as encompassing as practically possible and we have attempted to include representative papers from allied areas. Due to the broad scope of IMS and its wide variety of applications, by no means do we imply to give a complete overview of exploratory/unsupervised IMS analysis. However, we do hope that this review can serve as a context‐rich stepping stone or entry point for (i) analytical chemists and mass spectrometry experts who want to explore computational techniques; and (ii) computer scientists and data mining specialists who want to enter the IMS field. To guide the reader, we provide an overview of the discussed methods in Table 1, with a reference to the relevant section and a brief description of the method's demonstrated application area within the field of IMS.

**Table 1:** Method Index. Alphabetic index of methods treated in this review and the IMS application areas in which they have been demonstrated

Methods	Section	Demonstrated applications in IMS
Adaptive edge‐preserving denoising	Section III.E	Image segmentation incorporating spatial information
AMASS	Section III.F	Soft image segmentation, probability‐based model, built‐in feature selection
Artificial neural networks	Section IV	Nonlinear dimensionality reduction, image segmentation, visualization of high‐dimensional IMS data
Autoscaling	Section II.5	Data preprocessing
Autoencoders	Section IV	Nonlinear dimensionality reduction, image segmentation, visualization of high‐dimensional IMS data
Bisecting *k*‐means	Section III.C	Image segmentation, interactive exploration of clustering tree
Compressive sensing	Section II.B	Dimensionality reduction, increase spatial resolution
CX/CUR matrix decomposition	Section II.4	Non‐negative pattern extraction and unmixing (in context of IMS), data size and dimensionality reduction
DWT	Section II.B	Dimensionality reduction, feature extraction
FCM	Section III.F	Soft image segmentation
Filter scaling	Section II.5	Data preprocessing
GMM clustering	Section III.D	Image segmentation (hard and soft)
GSOM	Section IV.B	Nonlinear dimensionality reduction with built‐in dimensionality selection, image segmentation, visualization of high‐dimensional IMS data
HC	Section III.B	Image segmentation, interactive exploration of clustering tree
HDDC	Section III.D	Image segmentation (hard and soft), built‐in dimensionality reduction
ICA	Section II.C	Pattern extraction and unmixing, dimensionality reduction
*k*‐means clustering	Section III.C	Image segmentation, grouping of similar ion images
Kohonen map	Section IV.B	Nonlinear dimensionality reduction, image segmentation, visualization of high‐dimensional IMS data
Latent Dirichlet allocation	Section III.F	Soft image segmentation, probability‐based and generative model
MAF	Section II.D	Pattern extraction and unmixing incorporating spatial information, dimensionality reduction
MCR	Section II.1	Non‐negative pattern extraction and unmixing, dimensionality reduction
MCR‐ALS	Section II.1	Non‐negative pattern extraction and unmixing, dimensionality reduction
MNF transform	Section II.D	Pattern extraction and unmixing incorporating spatial information, dimensionality reduction
MOLDL	Section II.5	Non‐negative pattern extraction and unmixing (in context of IMS) using prior information, dimensionality reduction
MRF	Section III.E	Image segmentation incorporating spatial information
NMF	Section II.2	Non‐negative pattern extraction and unmixing, dimensionality reduction
NN‐PARAFAC	Section II.E.3	Non‐negative pattern extraction and unmixing, pattern extraction, dimensionality reduction
PCA	Section II.A	Pattern extraction and unmixing, data size and dimensionality reduction
pLSA	Section II.3	Non‐negative pattern extraction and unmixing, dimensionality reduction, generative and statistical mixture model
PMF	Section II.2	Non‐negative pattern extraction and unmixing, dimensionality reduction
Poisson scaling	Section II.5	Data preprocessing
Random projections	Section II.B	Dimensionality reduction
Shift‐variance scaling	Section II.5	Data preprocessing
SMCR	Section II.5	Non‐negative pattern extraction and unmixing, dimensionality reduction
SOM	Section IV.B	Nonlinear dimensionality reduction, image segmentation, visualization of high‐dimensional IMS data, image registration
Spatial shrunken centroids	Section III.F	Image segmentation (hard and soft), built‐in feature selection
Spatially aware clustering	Section III.E	Image segmentation incorporating spatial information
SVD	Section II.A	Pattern extraction and unmixing, dimensionality reduction
t‐SNE	Section IV.A	Nonlinear dimensionality reduction, image segmentation, visualization of high‐dimensional IMS data
Varimax	Section II.A.6	Improve interpretability of matrix decomposition

AMASS, algorithm for MSI analysis by semisupervised segmentation; DWT, discrete wavelet transform; FCM, fuzzy c‐means clustering; GMM, Gaussian mixture model; GSOM, growing self‐organizing map; HC, hierarchical clustering; HDDC, high dimensional data clustering; ICA, independent component analysis; IMS, imaging mass spectrometry; MAF, maximum autocorrelation factorization; MCR, multivariate curve resolution; MCR‐ALS, multivariate curve resolution by alternating least squares; MNF, minimum noise fraction; MOLDL, MOLecular Dictionary Learning; MRF, Markov random fields; NMF, non‐negative matrix factorization; NN‐PARAFAC, non‐negativity constrained parallel factor analysis; PCA, principal component analysis; pLSA, probabilistic latent semantic analysis; PMF, positive matrix factorization; SMCR, self modeling curve resolution; SOM, self‐organizing map; SVD, singular value decomposition; t‐SNE, t‐distributed stochastic neighborhood embedding.

## II FACTORIZATION

Matrix factorization techniques are an important class of methods used in unsupervised IMS data analysis. These methods take a large and often high‐dimensional dataset acquired by an IMS experiment, and decompose it into a (typically reduced) number of trends that underlie the observed data. This reduced representation enables the analyst to gain visual insight into the underlying structure of the IMS data, and it often exposes the spatial and molecular signals that tend to colocalize and correlate (usually under the assumption of linear mixing). Furthermore, as these techniques can provide a lower‐dimensional and lower‐complexity representation of the original data, they regularly serve as a starting point for follow‐up computational analysis as well.

### A Principal Component Analysis

Principal Component Analysis (PCA) (Jolliffe, 2002) is a widespread data analysis method, with applications ranging from finance (Brockett et al., 2002), ecology (Wiegleb, 1980), and psychology (Russell, 2002), to genetics (Wall, Rechtsteiner, & Rocha, 2003), image processing (Liu et al., 2010), and facial recognition (Turk & Pentland, 1991). It has been widely employed in IMS research and is the most commonly used multivariate analysis technique in SIMS‐based IMS (Graham & Castner, 2012). Early proponents of PCA as a data processing tool for SIMS include Gouti et al. (1999), Biesinger et al., (2002), and Pachuta (2004). PCA was also one of the first multivariate analysis techniques to be applied to MALDI IMS data (McCombie et al., 2005; Gerhard et al., 2007; Muir et al., 2007; Van de Plas et al., 2007b; Trim et al., 2008). Given the ubiquitous use of PCA in IMS analysis, we devote extra attention to the underlying principle of this technique. Throughout this review, we have tried to organize principles of methods and their particular interpretations and applications in an IMS context into separate subsections. This should allow the reader to read subsections relevant to their interests.

#### 1 Principle

The goal of PCA is to reduce the dimensionality of a dataset, that is, describe the dataset with a lower number of variables, while still retaining as much of the original variation as possible (Jolliffe, 2002). These new variables, called the principal components (PCs), are linear combinations of the original variables and each of them is uncorrelated to the others. This means that the PCs are constructed such that they give the orthonormal directions of maximum variation in the dataset. The first PC is defined such that it explains the largest possible amount of variance in the data. Each subsequent PC is orthogonal to the previous PCs and describes the largest possible variance that remains in the data after removal of the preceding PCs. This formulation ensures that most of the variance in the dataset is captured by the earlier PCs, while later PCs report consistently decreasing variance and thus often reduced impact on the data. In data that describes genuine instrumental measurements, it is often possible to account for most of the observed dataset variance with a number of PCs that is substantially smaller than the original number of variables, in which that dataset was described. By attempting to represent data using a smaller number of variables than the number of variables it was initially recorded with, PCA can help to uncover the underlying, or latent, structure of the data, which is often difficult to observe natively in high‐dimensional datasets such as IMS measurements. In current IMS datasets, the largest trends in the data are often captured by the first 10–20 PCs, making those PCs particularly useful for human exploration. These PCs essentially provide by means of 10–20 images (and their corresponding spectral signatures) a summarized view into the major underlying spatial and spectral patterns present in the data, side‐stepping the need to exhaustively examine hundreds to thousands of ion images individually.

PCA can be written in the form of a matrix decomposition. Let us take the data matrix D of size m×n, where m is the number of pixels and n is the number of spectral bins or m/z bins, that is, each row of D represents the mass spectrum of a pixel in the sample. PCA then decomposes D as

$$D=SL^{T},$$

where T represents matrix transposition, S is an m×p matrix with orthogonal columns, often called the score matrix, and L is an n×p matrix with orthonormal columns, traditionally called the loading matrix. The number of columns p in S and L is the number of PCs, and p=min(m,n). Since in many IMS experiments the number of spectral bins exceeds the number of pixels, in those cases m is smaller than n and thus the total number of PCs is limited to the number of pixels. In cases where the number of pixels m is larger than the number of variables per pixel n, which for example sometimes occurs in peak‐picked IMS data, the number of PCs is limited to n. The columns in S and L are ranked from high to low variance. If we wish to explore the k most important trends in the data (from a variance perspective) or wish to approximate the original dataset D as close as possible using only k variables instead of m or n variables, we need to retain only the k first PCs, that is, retain and store only the first k columns of S and L. By representing the data as a product of a score matrix and a loading matrix, and having variance directly accessible in terms of the columns of S and L, PCA can provide the best approximation of the data (with respect to the mean square error, and assuming linear mixing) using only k components, with k often being a user‐specified number. More formally, PCA provides the best rank‐k approximation of a dataset with respect to the L2‐norm (Jolliffe, 2002).

The PCA matrix decomposition of dataset D can be obtained by performing an eigenvalue decomposition of $D^{T}D$, a matrix that (if D is zero mean or column‐centered) is proportional to the covariance matrix of the measurements D up to a scaling factor 1/(N−1) with N as the number of samples. The PCA decomposition of D (and its underlying eigenvalue decomposition of $D^{T}D$) are generally, however, calculated via another closely related matrix decomposition called the singular value decomposition (SVD) (Golub & Van Loan, 1996), which can provide some additional insights into the meaning of S and L (Keenan & Kotula, 2004a). SVD decomposes D as

(1) $$D={U𝚺V}^{T},$$

where 𝚺 is an m×n matrix, which only contains nonzero elements on its diagonal (called the singular values), U is a m×m orthogonal matrix containing the left singular vectors of D, and V is an n×n orthogonal matrix containing the right singular vectors of D. By convention, the singular values are sorted from high to low, determining the order of the singular vectors. The number of nonzero singular values is determined by the rank of the matrix, and rank⁢ (D)≤min(m,n). Matrices U and V have specific meanings in case of a matrix D representing the IMS data (with each row of D representing the mass spectrum at a particular pixel). The left singular vectors in U form an orthonormal basis for the IMS experiment's pixel space. The right singular vectors in V form an orthonormal basis for the mass spectral space (Wall, Rechtsteiner, & Rocha, 2003). By using SVD (Equation 1), the covariance matrix of D (omitting the scaling factor) can now also be written as:

(2) $$\begin{array}{ll} C & =\;D^{T}D\\ & =\;{\left(U𝚺V^{T}\right)}^{T}(U𝚺V^{T})\\ & =\;V𝚺U^{T}U𝚺V^{T}\\ & =\;V{𝚺}^{2}V^{T}. \end{array}$$

It can be shown that the matrix V now actually contains the eigenvectors of the covariance matrix, which also form the PCA loadings L. When D is column‐centered, that is, the mean is subtracted for each column of D, the PCA loading matrix can be directly derived from the SVD results (and Equation 2) as:

L=V,

and the score matrix as:

S=U𝚺.

When PCA is applied as a dimensionality reduction technique, only the first k components (and columns) are retained, and these relationships become:

$$S_{k}={\left(U𝚺\right)}_{k},$$

$$\;L_{k}=V_{k}.$$

The PC loadings L are equivalent to the right singular vectors spanning the spectral space, and the PC scores S are the left singular vectors U spanning the pixel space, multiplied by the singular values 𝚺. From this comparison, it also becomes clear that performing PCA on the transpose of D,

$$D^{T}={\left(U𝚺V^{T}\right)}^{T}=V𝚺U^{T},$$

simply switches the positions of U and V around, as 𝚺 only contains scalar values on the diagonal. Due to the existence of efficient SVD algorithms and its superior numerical stability over eigenvalue decomposition of the covariance matrix (Wilamowski & Irwin, 2011), PCA is generally calculated using the SVD decomposition. Furthermore, given that only the first l=min(m,n) singular values are nonzero, only the first l PCs will be nonzero, and it therefore suffices to calculate only the first l PCs.

#### 2 Interpretation

**Figure 2:**
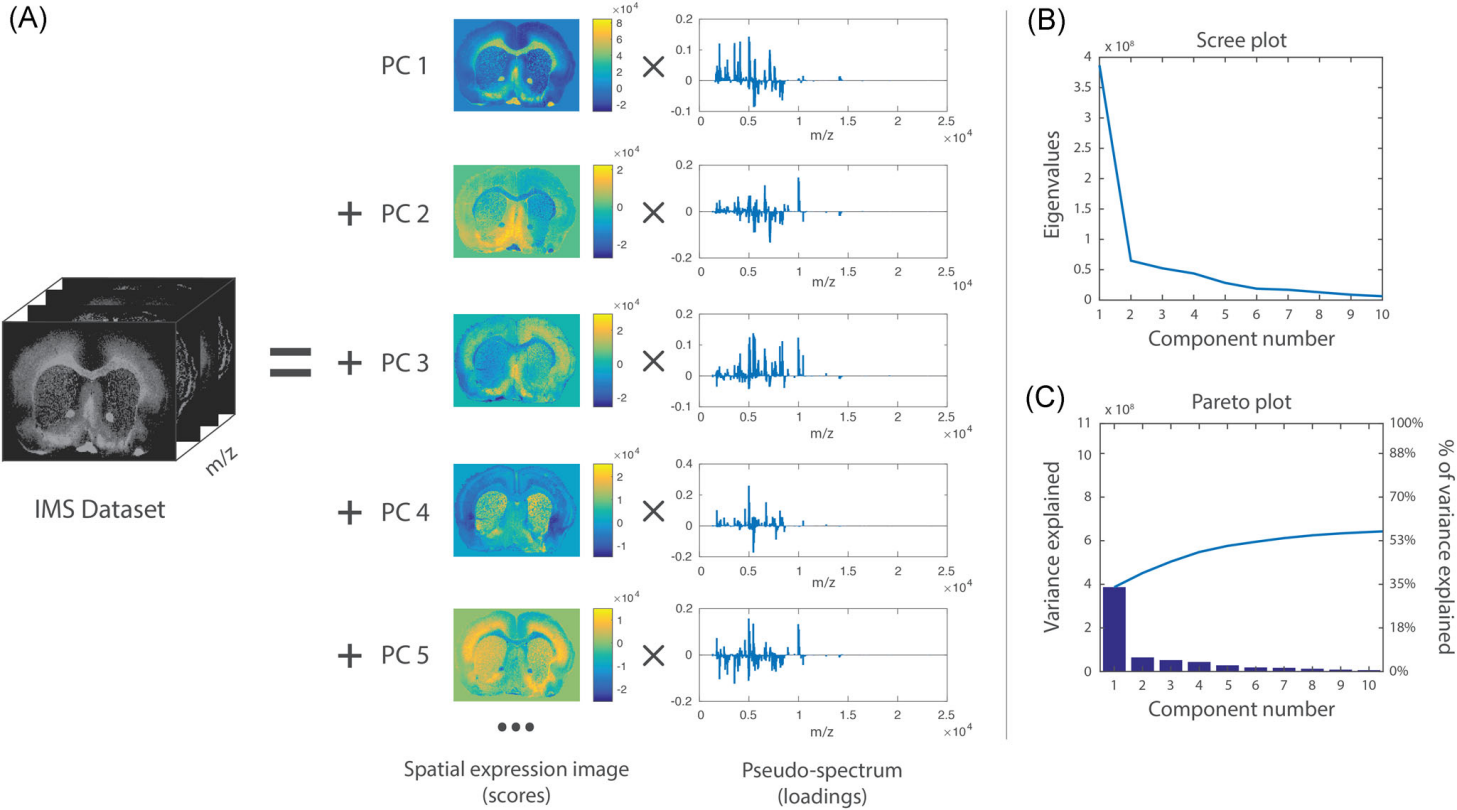
Example of PCA. PCA applied to a MALDI IMS dataset acquired from a coronal rat brain section of a Parkinson's disease model. Details of the dataset are available in Verbeeck et al., 2017. (**a**) PCA decomposes the original IMS dataset into a sum of principal components (PCs), where each component is characterized by a spatial expression image (score) times a pseudospectrum (loading). The PCs are ranked by the amount of variance they account for in the original dataset. The first five PCs are displayed, showing extraction of molecular patterns specific to various anatomical structures and with some exhibiting clear differences between the left and right hemispheres (as expected in this disease model). To estimate the number of relevant principal components for a dataset, a scree plot (**b**) or Pareto plot (**c**) can be used. These visualize the variance explained per PC, and can thus suggest a cut‐off threshold. IMS, imaging mass spectrometry; MALDI, matrix‐assisted laser desorption/ionization; PCA, principal component analysis. [Color figure can be viewed at wileyonlinelibrary.com]

The interpretation of the loadings and scores provided by PCA is not always straightforward (Wall, Rechtsteiner, & Rocha, 2003). An example of PCA applied to a MALDI IMS dataset acquired from a rat brain section of a Parkinson disease model (Verbeeck et al., 2017) is shown in Figure 2. The loading of each PC can be seen as a pseudospectrum, a spectral signature that explains a part of the variance of the dataset. The magnitude of the total explained variance for a component is codetermined by its scaling factor in the score matrix. Generally, it is not advisable to tie biological meaning directly to this pseudospectrum (Wall, Rechtsteiner, & Rocha, 2003). These pseudospectra constitute linear combinations of m/z bins, optimized to explain as much of the data variance as possible, but that is not necessarily the same as correctly modeling the underlying biology or sample content. One example of this is that PCA‐provided pseudospectra often contain negative peaks. These can be difficult to interpret from a mass spectrometry perspective since the original IMS data only contains positive values, namely ion counts. Regardless of how the sign of the intensity signal for a m/z bin in these pseudospectra is interpreted, it is clear that if its absolute signal intensity is relatively high, the m/z bin plays a role in explaining the overall variations and patterns observed in the data. Furthermore, the higher the absolute peak height for a particular m/z bin is within a PC's pseudospectrum, the larger its contribution to the variance accounted for by that component, and thus the larger its importance within the data trend captured by that component (Wall, Rechtsteiner, & Rocha, 2003). As such, without over‐interpreting the PCs, PCA can serve to highlight among thousands of measured m/z bins those bins that seem to play a more prominent role in the patterns that underlie the data.

Each loading vector has an accompanying score vector, which constitutes the spatial expression tied to the PC. These images obtained from the score matrix (and its equivalent in other factorization methods) can be considered spatial expression images or spatial mappings. The scores can be seen as the strength with which a loading is expressed at a particular location in the tissue or sample. These, again, consist of linear combinations of the pixels in the dataset, and thus negative values are generally included. Nevertheless, the spatial expression images of the k most important PCs often give a good overview of the k most prominent spatial patterns that make up the majority of measured variation in the IMS dataset, and can thus be very useful in gaining initial insight into the data. One helpful way of using these spatial expression images (scores) and their accompanying spectral signatures (loadings) is to employ them as guides to find correlating and anticorrelating ion peaks and ion images. Effectively, PCA can be used as a multivariate way of finding among thousands of pixels those that show similar spectral content, or among thousands of ions those that exhibit a similar spatial distribution. A less powerful, univariate version of this task can also be explored using correlation, which will be discussed in greater detail in Section III.G.

#### 3 Number of Components

Determining the correct number of components to retain after a PCA analysis is an important but difficult challenge to tackle, and several papers have been dedicated to the subject (Peres‐Neto et al., 2005). As mentioned by Jolliffe (2002), there is no straightforward solution to the problem available. One of the most commonly used and easiest methods to determine the number of components is by means of a scree plot (Cattell, 1966) (Fig. 2b), which consists of plotting the eigenvalues of the PCs in descending order, and selecting a cut‐off at the point where there is no longer a significant change in the value of two consecutive eigenvalues. Alternatively, a Pareto plot (Fig. 2c) can be used, which plots the cumulative percentage of variance explained by each consecutive PC. Here, one can either use a predefined threshold of what percentage of variance must be maintained, or, similar to the scree plot method, select a cut‐off point when there is no longer a significant change in percentage explained. Determining the correct number of components is a recurring issue for various pattern extraction techniques in IMS, and various methods exist that can be readily applied, including the minimum description length (Rissanen, 1978; Verbeeck, 2014b), the Bayesian information criterion (BIC) (Schwarz, 1978; Hanselmann et al., 2008), the Akaike information criterion (AIC) (Akaike, 1973), and the Laplace method (Minka, 2000; Verbeeck, 2014b).

#### 4 Application to IMS

Graham & Castner (2012) lists applications of PCA in SIMS imaging, along with other multivariate analysis methods. Recent work by Bluestein et al. (2016) shows the use of PCA in lipid‐focused TOF‐SIMS data, acquired from breast cancer tissue, using PCA as a means of selecting regions of interest (ROIs). Fletcher et al. (2011) used PCA for data reduction and visualization of 3D TOF‐SIMS measurements collected from HeLa‐M cells, allowing for visualization of different cellular components such as the membrane and nucleus. Additionally, PCA provided a method to adjust and register the cellular signal along the *z* axis.

PCA has also been extensively used in DESI‐based imaging. Dill et al. (2009) used PCA to distinguish tumor regions and associated lipid profiles in DESI IMS data, acquired from canine bladder cancer samples. In follow‐up work (Dill et al., 2011), PCA was applied to DESI imaging data of a relatively large set of 20 human bladder carcinomas, to investigate the variation within and between both healthy and diseased tissues. The first PC showed a high expression in the tumor tissue and a low expression in the healthy tissue, indicating a strong difference in molecular expression between the two tissue types. However, as PCA does not provide explicit classification rules, a follow‐up with a supervised approach, namely orthogonal projection to latent structures (O‐PLS), was used to create a classification model for these samples. Pirro et al. (2012) have used PCA to provide an interactive way to explore DESI IMS data, while Calligaris et al. (2014) employed PCA in the characterization of biomarkers in DESI imaging data collected from breast cancer samples.

PCA has also found application in the analysis of rapid evaporative ionization mass spectrometry (REIMS) imaging data of human liver samples and bacterial cultures, where it enabled differentiation between healthy and cancerous tissue and between three bacterial strains (Golf et al., 2015).

In MALDI IMS, there has been extensive use of PCA both for aiding human interpretation of IMS data, as well as for reducing the dimensionality and size of the data to enable subsequent computational analysis. Examples include McCombie et al. (2005), who used PCA to extract spatial trends from MALDI IMS measurements acquired from the mouse brain, employing the technique primarily as a dimensionality reduction step preceding further data analysis. Gerhard et al. (2007) used PCA in the analysis of MALDI IMS data acquired as part of a clinical breast cancer study, with spatial expression images correlating well with the histology and showing a clear separation of the different tissue parts. Deininger et al. (2010, 2008) used PCA in the analysis of MALDI IMS of gastric cancer tissue sections, where the first PCs reflected the histology quite well. However, they noted that the PC loadings and scores were difficult to interpret, particularly when compared with hierarchical clustering (HC) (see Section III.B), which can summarize the data in a single image rather than spread out over multiple components. Siy et al. (2008) applied PCA in the analysis of MALDI IMS data from the mouse cerebellum, and compared the results to those of independent component analysis (ICA) and non‐negative matrix factorization (NMF), which we discuss in Sections II.C and II.E, respectively. While PCA allowed identification of the major spatial patterns, both the loadings and scores were noisier than those of NMF and ICA. Hanselmann et al. (2008) used PCA in a comparison with pLSA, ICA, and NN‐PARAFAC on SIMS as well as on MALDI IMS datasets. In this setting, PCA was found to be the least successful of the compared techniques for extracting spatial patterns from the dataset. Furthermore, Hanselmann et al. note that the pseudospectra are difficult to interpret due to the negative values they contain. Gut et al. (2015) included PCA as one of several matrix decomposition methods to analyze MALDI IMS data acquired from pharmaceutical tablets. In this study, PCA allowed for good retrieval of sources of variation in the data, but provided limited spectral information compared to the other matrix decomposition methods used.

Due to the ubiquity of PCA in IMS research, subsequent subsections focus primarily on papers that made changes to the standard PCA workflow, adapting it specifically toward application in an IMS setting.

#### 5 Preprocessing—Scaling

While PCA offers a robust method for the extraction of underlying components, statistical preprocessing of the data can have a large impact on the decomposition results. This topic has been studied in depth in the SIMS imaging community (Keenan & Kotula, 2004a; Tyler, Rayal, & Castner, 2007). There are several reasons for this. First, preprocessing will influence the impact or weight that a single variable has in the overall PCA analysis. PCA pushes variables toward more important PCs on the basis of how much variance the variables represent in the data. In the case of IMS data, high‐intensity peaks that vary will tend to represent a much larger part of the overall variance than low‐intensity peaks that vary in a similar way. As a result, tall peaks can exert a greater influence on the retrieved PCs than short peaks. On the one hand, this is a desirable effect: large differences in high intensity peaks are often very informative, and should therefore be reported prominently. On the other hand, these peaks can be so dominant that changes in relatively small, yet biologically relevant, peaks can be lost in the overall PCA decomposition of a dataset, as these small peaks are not essential to explaining the majority of variance in the data. One way of countering this phenomenon is by scaling the variables prior to performing PCA analysis.

5.1

###### Autoscaling

One popular method is autoscaling (Jackson, 1991; Keenan & Kotula, 2004a; Tyler, Rayal, & Castner, 2007), where each variable has its mean subtracted and is divided by its standard deviation. This scaling is equivalent to performing the PCA analysis on the correlation (coefficient) matrix instead of the covariance matrix. Autoscaling makes each m/z bin exhibit unit variance, thus giving high and low intensity peaks equal influence in the PCA analysis (Tyler, Rayal, & Castner, 2007; Deininger et al., 2010). It is important to note though that this type of scaling can allow noisy low‐signal m/z bins to have an increased (and sometimes disproportionate) impact on the PCA analysis results, potentially delivering noisy uninformative PCs in the process.

###### Poisson Scaling

Besides the weight that a variable (pixel or m/z bin) has in an overall PCA decomposition, a more fundamental reason to apply statistical preprocessing or scaling prior to PCA lies in the underlying assumptions behind PCA. Due to the fact that PCA finds relationships between features based on their variance, and a Gaussian distribution is fully determined by its variance and mean, PCA works optimally on data with a Gaussian distribution. If the data are not Gaussian‐distributed, however, there are usually higher‐order statistics beyond variance present that are not being taken into account by PCA. While PCA captures components that are uncorrelated, these components are not necessarily statistically independent for general distributions, a topic we will discuss in greater detail in Section II.C. For example, the spectra in TOF‐based mass spectrometry are typically formed by counting the number of ions that hit a detector. This means that both the noise and variability of the signal are likely to be governed by Poisson statistics (Keenan & Kotula, 2004a) and will not necessarily approximate a Gaussian distribution. One of the further consequences of a Poisson distribution of ion counts is that the variance, and thus uncertainty, of an ion intensity measurement is directly proportional to the magnitude of the measurement (the mean and variance of a Poisson distribution are identical to each other). Thus, high peaks will tend to have higher noise intensities than low peaks, which will again influence their importance in the PCA decomposition.

This aspect also plays an important role in the selection of the correct number of PCs: the graph of eigenvalues or scree plot that is commonly used to select the correct number of PCs should be near‐zero for components that only describe (Gaussian) noise, and should take substantial values for components that describe systematic and structural information (Malinowski, 1987). However, this is not the case for variables that report a non‐Gaussian noise distribution, as is illustrated in Keenan & Kotula (2004a). Consequently, noisy but high‐intensity components can rank very high in the order of PCs, whereas small but relevant peaks can end up in components with a substantially lower rank, and might go unrecognized as a result. For this reason, a popular scaling method, especially in SIMS research, is the Poisson scaling described in Keenan & Kotula, (2004a). This is essentially a form of weighted PCA that aims to mitigate the effects of the Poisson distributed noise, by transforming the variables to a space better suited for PCA analysis, prior to performing the actual PCA analysis. This form of scaling involves dividing each row of the matrix D by the square root of the mean spectrum row vector, and dividing each column of the data matrix by the square root of the mean pixel (or image) column vector. In a related setting, Wentzell et al. (1997) applied a maximum likelihood PCA (MLPCA) approach to compensate for deviating noise structure in the PCA of near infrared spectra. Keenan (2005) compared Poisson‐scaled PCA to MLPCA on simulated SIMS data with known amounts of Poisson noise, and showed that, while MLPCA performed the best in extracting the original components, Poisson‐scaled PCA performed nearly as well, at a much lower computational cost. In another comparison of scaling methods, Keenan & Kotula (2004b) show that Poisson scaling outperforms autoscaling as a statistical preprocessing method.

###### Filter Scaling and Shift Variance Scaling

Several other scaling methods exist, including filter scaling (Tyler, Rayal, & Castner, 2007), which consists of dividing each peak by the standard deviation of a set of pixels near that peak, in order to account for local intensity variation and noise, and shift variance scaling (Tyler, Rayal, & Castner, 2007), which is based on the same concept as maximum autocorrelation factorization (MAF), a matrix factorization method akin to PCA that we will discuss in Section II.D. Briefly, shift variance scaling entails dividing each peak by its standard deviation in the shift matrix, a data matrix obtained by subtracting from the original data matrix a copy that has been spatially shifted by a pixel horizontally and/or vertically. In a comparison of PCA using autoscaling, Poisson scaling, filter scaling, shift variance scaling, and MAF, Tyler et al. (2007) demonstrated that MAF performed best, albeit at the cost of greater computation time, while Poisson‐scaling and shift variance scaling provided results similar to MAF at a lower computational cost.

###### Intensity Scaling to Incorporate Prior Knowledge

Besides its use for noise correction, weighted PCA by means of scaling also provides the tools for directly incorporating domain‐specific knowledge into the analysis. Scaling the absolute intensity values of specific m/z bins on the basis of prior knowledge provides a mechanism for giving known ions of interest a greater weight, and thus pushing a normally unsupervised PCA algorithm to identify, in a more supervised way, ion species that correlate and anticorrelate with the specific ions of interest. In a similar fashion, the weight of known noise peaks or ion species that are not relevant to the biological mechanism at hand, can be diminished in the overall decomposition, essentially clearing up PCA bandwidth for patterns of interest. By downscaling undesirable peaks, their variance is reduced and consequently their corresponding components move into a less prominent position in the PC ranking. This essentially allows for prior knowledge to be straightforwardly incorporated into PCA, without a fundamental change to the underlying algorithm, and provides a means for dynamically testing the influence of ion species on the final PCA result (by testing the response to scaled versions). With these uses in mind, we demonstrated on MALDI IMS data the influence of weighted PCA (Van de Plas et al., 2007a). The scaling was done using digital image enhancement techniques, specifically gray level transformations on the basis of histograms, to enhance the contrast of individual ion images and eliminate noise patterns, while concurrently leading to a reduction in the resulting PCA expression images.

Although the issue of scaling is treated here within the context of PCA, it is clear that all unsupervised data mining methods can be influenced by it. This can serve different purposes, including accounting for underlying algorithm assumptions, noise reduction, as well as contrast enhancement and incorporation of prior knowledge.

#### 6 Postprocessing—Varimax

PCA, and several other factorization methods, suffer from what is known as “rotational ambiguity.” This means that there are an infinite number of orientations of the factors that account for the data equally well (Russell, 2002). More precisely, given a matrix factorization

$$D=AB^{T},$$

and any invertible transformation matrix R, the matrix D can also be written as

(3) $$D=(AR)(R^{-1}B^{T})=\tilde{A}{\tilde{B}}^{T}.$$

In other words, there exist an infinite number of factor pairs $\tilde{A}$ and $\tilde{B}$ that will fit the data equally well (Keenan, 2009). PCA aims to find the solution to this factorization where the matrices have mutually orthogonal vectors, and the components serially represent maximal variance in the original data. As stated, PCA provides the best rank‐k approximation of the data (in a least squares sense). This means that PCA aims to represent as much information as possible, using as few components as possible. This setup makes sense when PCA is used as a dimensionality and data reduction tool; however, it also leads to very “dense” components, components with many nonzero weights, which makes them difficult to interpret by humans. It is therefore often useful to apply a rotation of the resulting PCA loading vectors in order to simplify the factor model for human examination (Russell, 2002). Similar to Equation 3, this rotation does not affect the goodness of fit of the solution, or more formally: the subspace defined by the PCs remains the same. However, we are selecting a different solution among all the equally valid solutions of rank k (Paatero & Tapper, 1994). A rotation of the loadings will, however, relax the orthogonality constraints on the scores when projected on the new loadings, that is, the scores will no longer be uncorrelated (Jolliffe, 2002; Keenan, 2009).

**Figure 3:**
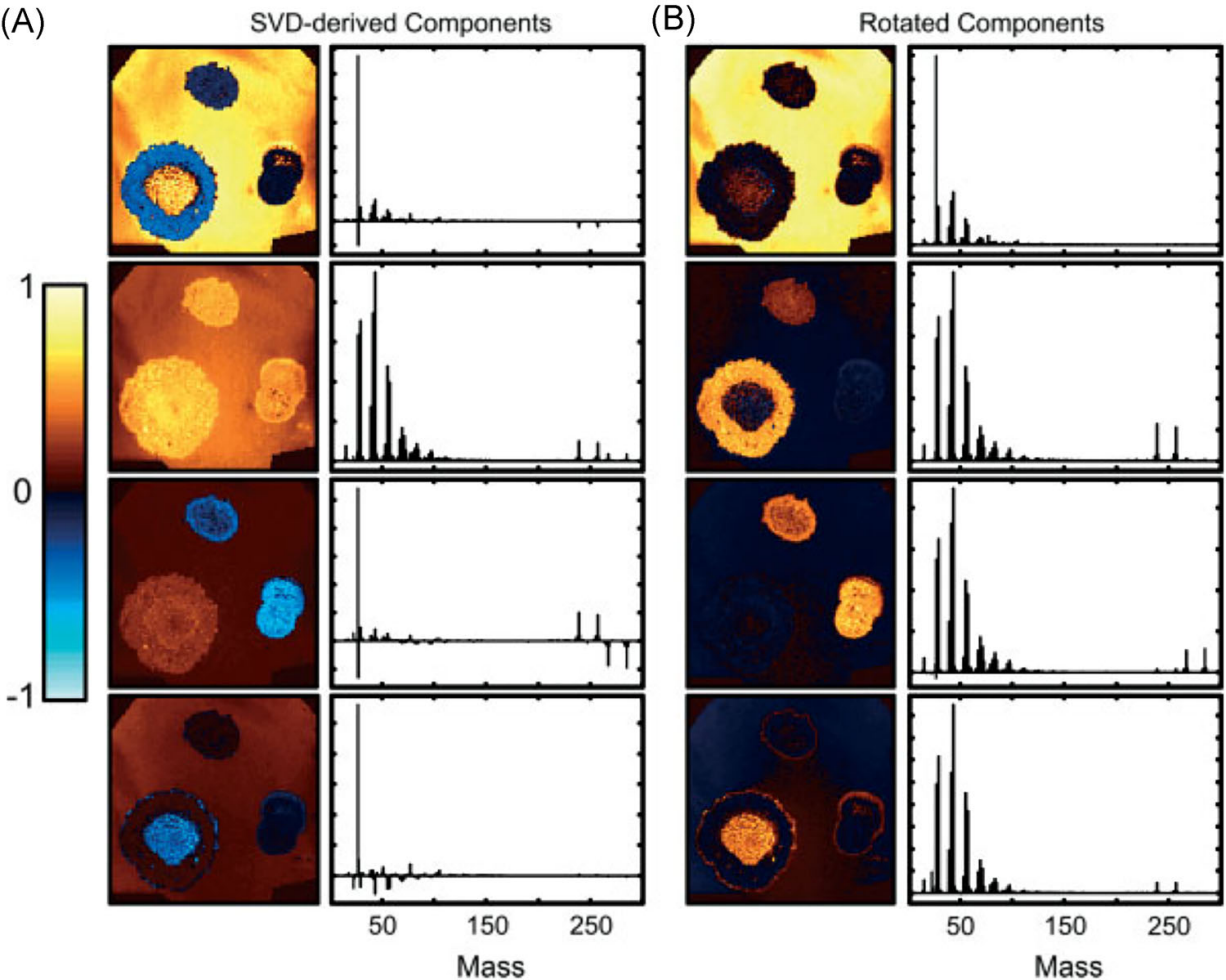
Example of Varimax rotation. Original caption: *Comparison between (**a**) the abstract factors obtained by SVD with (**b**) the same factors after Varimax rotation to maximize spatial‐domain simplicity for the palmitic/stearic acid sample*. Source: Keenan, 2009, Figure 7, reproduced with permission from John Wiley & Sons. SVD, singular value decomposition. [Color figure can be viewed at wileyonlinelibrary.com]

One of the most frequently used criteria for rotation of PCA components is the Varimax rotation proposed by Kaiser (1958), which performs an orthogonal rotation that maximizes the variance of the squared loadings. This approach results in loading vectors that are more sparse, that is, have many elements that are zero, while having a lower number of nonzero loading coefficients with higher absolute values. In the case where the loadings are the spectral features, this means fewer m/z bins that are nonzero, which greatly improves the interpretability of the loading components. Klerk et al. (Broersen, Van Liere, & Heeren, 2005; Klerk et al., 2007) have applied Varimax rotation to PCA components in LDI and SIMS imaging datasets. Performing the Varimax rotation on the chemical loadings increased calculation times only slightly, but led to a high increase in contrast in the spatial expression images, while simultaneously leading to sparser chemical component spectra with higher peaks. When comparing the results to those obtained through non‐negativity constrained parallel factor analysis (NN‐PARAFAC; see Section II.E), PCA + Varimax offered the best trade‐off between calculation time and result quality. On the other hand, NN‐PARAFAC offered the advantage that loadings were positive or zero, more closely approximating the non‐negative ion counts naturally encountered in MS. Due to its negligible calculation time, the authors recommended the use of Varimax as a default postprocessing step after PCA for improved interpretability. Fornai et al. (2012) used PCA + Varimax in the investigation of a 3D SIMS dataset of rat heart, consisting of over 49 billion spectra collected from 40 tissue sections. Keenan (2009) has applied Varimax rotation in SIMS data on the spatial‐domain components rather than the spectral components, which resulted in a higher contrast for the expression images, as shown in Figure 3. Furthermore, due to the fact that this was a relatively uncomplicated sample with only a few components present in each spatial location, the spectral components are relatively simple, making them similar to those obtained through MCR‐ALS, a NMF method discussed in Section II.E.

Despite the many strengths of PCA, that is, availability of an analytical solution, ease of use, existence of optimized algorithms (see Intermezzo below), and availability in many software packages, several disadvantages remain. One disadvantage is the presence of negative peaks in the pseudospectra and negative values in the spatial expression images, which can lead to difficulties in interpreting PCA results (Deininger et al., 2010). Another is that the assumptions underlying PCA do not necessarily agree with the characteristics of IMS data. Fortunately, the method has shown remarkable robustness to such violations of its assumptions, and continues to provide useful insight into IMS data regardless. An issue that we have not yet discussed is that PCA treats each pixel as an independent sample. This is technically not true in imaging data if detectable levels of spatial autocorrelation are present in the measurements (Van de Plas et al., 2015; Cassese et al., 2016), and it merits further investigation. Furthermore, standard PCA does not take the available spatial information into account in its decomposition, an aspect that could be used to improve the analysis further. Some of these disadvantages have been addressed by alternative matrix factorization approaches, which will be discussed in the next sections.

### B Intermezzo: Dimensionality Reduction and Computational Resources

Current state‐of‐the‐art IMS datasets can reach massive sizes, with raw data ranging in the GBs and TBs for a single experiment, typically containing $10^{4}$–$10^{6}$ m/z bins per pixel, and $10^{3}$–$10^{6}$ or more pixels, depending on the instrumentation used. When applying factorization or clustering techniques to these data, their sheer size can easily lead to computer memory shortages and very long calculation times on standard desktop computers. A common method to deal with these issues is to perform a feature selection step such as peak‐picking, which precedes the data analysis (McDonnell et al., 2010; Jones et al., 2012a; Alexandrov et al., 2010; Du, Kibbe, & Lin, 2006). This greatly reduces the number of variables, and consequently the size of the data, by eliminating from the analysis the spectral bins that do not contain peak centers. Peak picking is, however, a rather drastic form of feature selection that discards a large amount of information from the original data (e.g., peak shape), while also holding the risk of discarding peaks that go unrecognized by the peak‐picking algorithm. This makes the quality of the subsequent analysis dependent on the quality of the preceding peak‐picking or feature selection method, which may not always be desirable (Palmer, Bunch, & Styles, 2013, 2015). Furthermore, IMS datasets with large numbers of pixels may still be too large to be analyzed even after peak‐picking (Halko, Martinsson, & Tropp, 2011; Race et al., 2013). Other methods that are sometimes used to reduce the size of the data are spectral binning (Broersen et al., 2008a) and spatial binning (Henderson, Fletcher, & Vickerman, 2009), which average (or sum) ion intensities over multiple m/z bins and pixels, respectively. While these methods sometimes increase the signal‐to‐noise‐ratio, they inherently lead to loss of spatial or mass resolution, which is usually undesirable.

As a result, several groups have investigated efficient algorithms and techniques capable of handling these large amounts of data without the need for peak picking and with minimal loss of information.

#### 1 Memory‐Efficient PCA

A number of studies have focused specifically on the development of memory‐efficient PCA algorithms, which allow PCA analysis to be performed despite large data sizes. Race et al. (2013) have applied a memory‐efficient PCA algorithm that allows sequential reading of the pixel data, and thus does not require keeping the full dataset in computer memory. This allows for processing of very large datasets, albeit that the full covariance matrix must still be constructed and therefore extremely high‐dimensional datasets may still prove to be problematic. This method allowed for processing of a 50 GB 3D MALDI IMS dataset that was previously too large to analyze. A MATLAB (The Mathworks Inc., Natick, MA) toolbox by Race et al. (2016) carries an implementation of this method, and allows application of this and other IMS data mining algorithms without loading the full data into memory. Klerk et al. (2007) used the PCA algorithms in MATLAB together with its sparse data format to exploit the sparse nature of IMS data, that is, the fact that many of the values measured in IMS are near‐zero. Rather than loading the full data matrix, this MATLAB format only stores nonzero values, which allows for much larger datasets to be loaded into memory. They used the native MATLAB algorithms to solve the eigenvector problem for sparse matrices, using restarted Arnoldi iteration (Sorensen, 1992). Cumpson et al. (2015) have compared four different SVD algorithms for PCA in SIMS imaging data. The “random vectors” SVD method proposed by Halko et al. (2011) was selected. Similar to the method of Race et al., it did not require loading the full dataset into memory. This method was used to analyze a 134 GB TOF‐SIMS imaging dataset in around 6 hr on a standard desktop PC. In recent work, Van Nuffel et al. (2016) achieved good decomposition results using a random subsampling approach to perform PCA in a 3D TOF‐SIMS dataset from an embryonic rat cortical cell culture. The loadings were calculated using a training set consisting of only 6.11% of the total number of pixels, and the IMS data was then projected on the loading vectors to create the (score) images. Cumpson et al. (2016) have similarly used a subsampling approach for the PCA analysis of large size 3D SIMS datasets, although here quasirandom Sobol sampling was used to obtain a more even spatial sampling throughout the sample. Graphical processing units (GPUs) were used to speed up the calculation of the PCs, as has also previously been demonstrated by Jones et al. (2012b) for PCA, pLSA, and NMF (see Section II.E). The quasirandom subsampling strategy used by Cumpson et al. has also been used by Trindade et al. (2017) in the calculation of the NMF decomposition of 3D TOF‐SIMS IMS data with very high spatial resolution.

Broersen et al. (2008a) have alleviated some of the memory and calculation constraints of PCA by using a multiscale approach, in which PCA is first calculated on a rebinned version of the data, grouping together multiple m/z bins (e.g., through averaging). It reduces the number of variables and the size of the data, while increasing the signal‐to‐noise ratio. In an interactive approach, the user can then select features of interest, which can be zoomed in on, and the PCA can be recomputed (without rebinning) for that smaller area or reduced set of features. In other work, Broersen et al., 2008a showed how the denoising effect and dimensionality reduction provided by PCA can be used in the alignment and combination of multiple SIMS datasets, imaging different areas of a large sample.

#### 2 Non‐PCA Dimensionality Reduction

Besides peak‐picking and PCA, many other data reduction and feature extraction/selection techniques exist and allow for high levels of data compression while retaining much of the original information. These are often less memory‐intensive than PCA or can be serially applied on individual spectra, avoiding the need to load the full IMS dataset into memory. For example, we have demonstrated the application of the Discrete Wavelet Transform (DWT) for the dimensionality reduction of IMS data (Van de Plas, De Moor, & Waelkens, 2008; Van de Plas, 2010; Verbeeck, 2014b). DWT is a popular data transformation that allows compact representation of the original mass spectra in a much lower‐dimensional wavelet coefficient space, with arbitrarily little loss of signal or information. DWT‐based compression greatly reduced the size of MALDI IMS datasets, and resulted in a 63‐fold reduction in memory requirements when performing PCA on a MALDI TOF IMS measurement set, while obtaining comparable PCs to those calculated without prior dimensionality reduction. Furthermore, we used this technique (Van de Plas, 2010) to reduce the dimensionality of a 19.6 GB MALDI FTICR IMS dataset by a factor of 128, after which the reduced data could be successfully analyzed through k‐means clustering (see Section III.C), showing clear overlap with biologically significant histological features.

**Figure 4:**
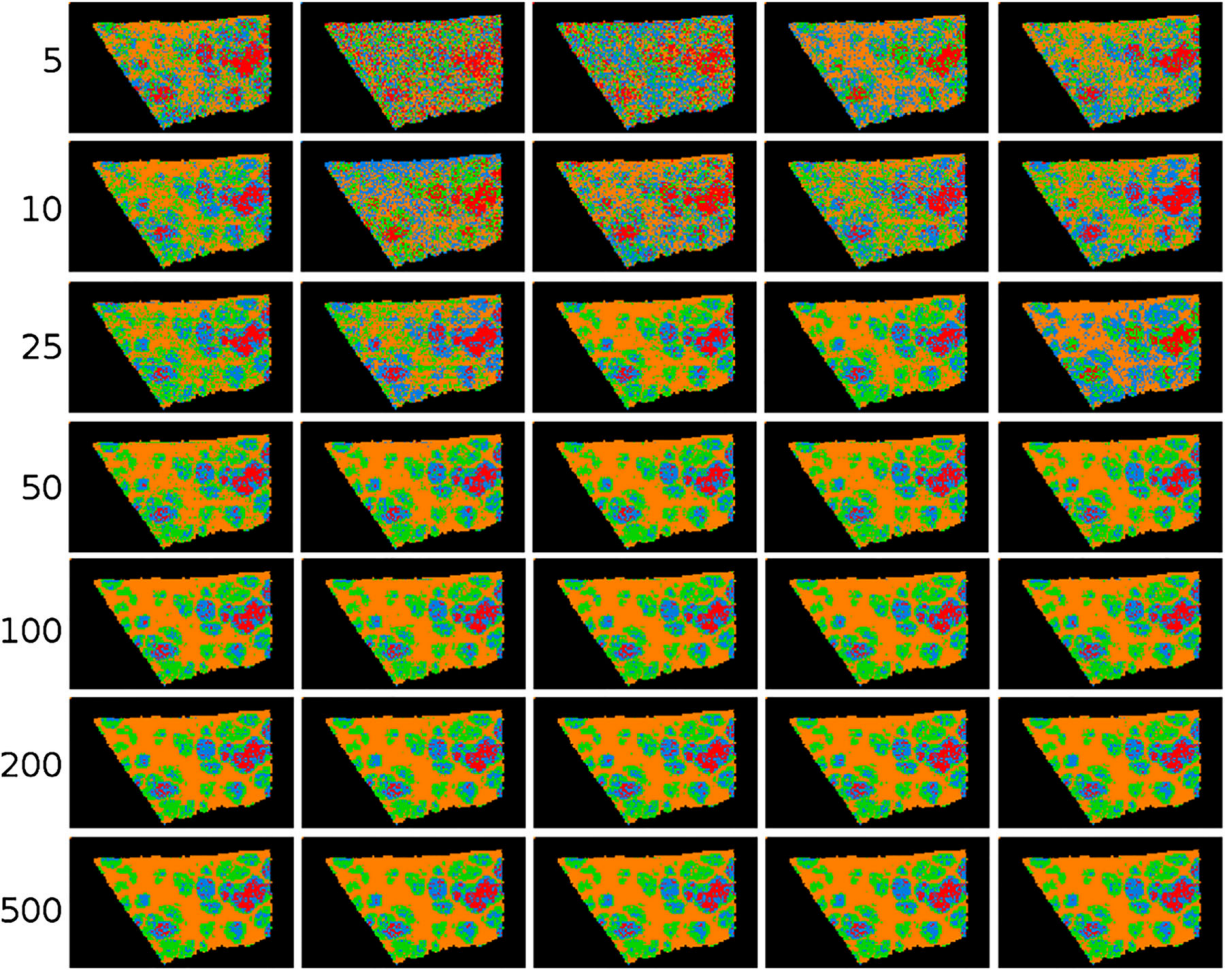
Example of k‐means clustering performed on random projections. Original caption: *Top‐to‐bottom: increasing the number of projections up to around 100 increases the segmentation reproducibility; after this point, the segmentation result completely stabilizes and the same tissue patterns are produced. Left‐to‐right: each column is the result of a different set of random vectors. At low numbers of projections, the exact choice of projection vectors affects the results of the segmentation, whereas for higher numbers of projections, the segmentation is stable and reproducible against a different choice of projection vectors*. Source: Reproduced from Palmer et al. (2015), Figure 4, under Creative Commons Attribution License. [Color figure can be viewed at wileyonlinelibrary.com]

In other work, Palmer et al. (2013) have investigated the use of random projections for dimensionality reduction of hyperspectral imaging datasets, such as MALDI IMS and Raman spectroscopy data. The full original IMS data are projected onto a set of randomly chosen vectors, rather than a calculated set of basis vectors as is the case in, for example, PCA. By projecting the data onto randomized vectors, a large part of the data redundancy that is often present in IMS data due to colocalization and correlation is removed, which reduces the data size, while preserving distances between points and angles between vectors, a property that is desirable in many machine‐learning approaches. Using this approach allowed impressive 100‐fold reductions in dimensionality and data size, while still allowing reconstruction of the original data within noise limits. In follow‐up work, Palmer et al. (2015) demonstrated consistent clustering results that followed histologic changes, using less than one percent of the original data, as is demonstrated in Figure 4.

#### 3 Compressive Sensing

A method for reducing the measurement dimensionality of IMS, and thus also keeping computational and instrumental requirements to a minimum, is the use of a compressive (or compressed) sensing framework. Compressed sensing builds on the famous Whittaker‐Nyquist‐Shannon‐Kotelnikov sampling theorem, which establishes the minimum sampling frequency required to fully reconstruct a signal. Candès, Tao, and Donoho (Candès & Tao, 2006; Donoho, 2006; Candès & Wakin, 2008) have posed that, given knowledge on the signal's sparsity, the original signal may still be reconstructed with fewer samples than the sampling theorem technically requires. Compressed sensing has been applied in various domains such as image reconstruction (Mairal, Elad, & Sapiro, 2008), hyperspectral imaging (Golbabaee, Arberet & Vandergheynst, 2010), and clustering (Guillermo, Sprechmann, & Sapiro, 2010). In the case of IMS, this would enable a reduction in the number of samples (pixels) collected in tissue, and enable the computational processing of smaller datasets. Gao et al. (2014) have demonstrated the use of compressed sensing in DESI imaging data, where it was used to reconstruct ion images using only half the sampling points in the original data. Only 40 out of approximately $10^{4}$ m/z values were identified as important to allow this reconstruction. Bartels et al. (2013) used compressed sensing with the assumption of sparse image gradients and compressible spectra, to reconstruct smoothed ion images using different fractions of samples of the original data (20–100%). Tang et al. (2015) have used compressed sensing to increase the spatial resolution in AFAI‐IMS data. To demonstrate this approach, the spatial resolution of the original imaging data was lowered by merging pixels. The compressed sensing approach was then used to reconstruct the image at the original resolution. Results were compared to bicubic interpolation, with compressed sensing reconstruction showing improved peak signal‐to‐noise‐ratio over the bicubic interpolation approach. In a similar application, Milillo et al. (2006) have used geospatial statistics, namely Kriging and inverse distance squared weighted methods, to reconstruct ion images from SIMS data, achieving reconstruction with recognizable images using only 10% of the original pixels in the image.

To conclude this intermezzo, it is worth noting that in recent years there has been increased use of high‐performance computing (HPC) resources in the processing of IMS data. For example, Smith et al. (2015) used HPC in the exploration of large FTICR imaging datasets. Further support comes from the emergence of HPC‐capable data processing environments such as openMSI (Rübel et al., 2013; Fischer, Ruebel, & Bowen, 2016). It is to be expected that this trend toward HPC will continue and even accelerate over the coming years, as IMS datasets grow ever larger due to advances in mass spectrometry instrumentation.

### C Independent Component Analysis

ICA is a matrix decomposition technique that originated from the area of blind source separation, and that aims to find statistically independent components that underlie the observed data (Jutten & Herault, 1991; Comon, 1994). ICA has been used extensively in a wide range of applications, including facial recognition (Bartlett, Movellan, & Sejnowski, 2002), functional magnetic resonance imaging (fMRI; Calhoun, Liu, & Adalı, 2009), and remote sensing (Du, Kopriva, & Szu, 2005; Wang & Chang, 2006). In IMS research, ICA has been used in the analysis of MALDI IMS measurements (Hanselmann et al., 2008; Siy et al., 2008; Verbeeck, 2014b; Gut et al., 2015), but, to our knowledge, not yet in other IMS varieties.

#### 1 Principle

While PCA and ICA are similar in premise, ICA requires its components to be statistically independent of each other, where PCA requires its components only to be uncorrelated. The requirement by ICA is stronger than that imposed by PCA: if two variables x and y are statistically independent, they are also uncorrelated, however, uncorrelated variables are not necessarily independent. Another distinction between PCA and ICA is that ICA assumes the underlying sources to follow a non‐Gaussian distribution, and in fact exploits this non‐Gaussianity for their recovery (Hyvärinen, 2013). While the assumption of non‐Gaussianity may seem limiting at first, many real‐life signals are in fact non‐Gaussian when considering the probability distribution of the signal (De Lathauwer, De Moor, & Vandewalle, 2000). Siy et al. (2008) note that the probability distribution of IMS data are highly non‐Gaussian given the large number of near‐zero values in a typical mass spectrum, and as we have previously noted (see Section II.A), the peaks in mass spectrometry signals tend to be Poisson‐distributed for most varieties of mass spectrometry. Furthermore, biological or other sample‐specific processes, underlying the observed mass spectra, could give rise to non‐normally distributed signals (Hebenstreit & Teichmann, 2011; Dobrzyński et al., 2014). In ICA, non‐Gaussianity of signals is an integral part of the analysis and is even utilized to accomplish its task. In PCA, most use cases will deal with non‐Gaussianity prior to analysis, and apply some form of statistical preprocessing (e.g., Poisson scaling) to make the data and noise more Gaussian‐like before doing PCA analysis.

Rather than a single technique, ICA constitutes a class of methods that use assumptions of independence and non‐Gaussianity to find underlying components. A number of different strategies exist and the ICA approaches can be roughly divided into two categories, although many ICA algorithms combine both: (1) approaches primarily rooted in information theory, which are aimed at minimizing mutual information between the components (or maximizing entropy exhibited by the components), such as the popular Infomax algorithm (Linsker, 1988); and (2) approaches aimed at finding maximally non‐Gaussian signals using higher‐order statistics (De Lathauwer, De Moor, & Vandewalle, 2000) such as kurtosis (skew or tailedness of the probability distribution) and negentropy (deviation from Gaussianity), for example, JADE (Cardoso & Souloumiac, 1993) and fastICA (Hyvärinen & Oja, 1997).

ICA is generally performed as a two‐step process. First, the data are whitened, and then the whitened data are used to retrieve the independent components. Whitening entails that the data are transformed to a new set of variables, such that its covariance matrix is the identity matrix, meaning that the new variables are decorrelated and all have unit variance. This is often done by performing PCA and then normalizing the PCs to unit length, but other approaches exist and it is important to note that this whitening step is not unique. Whereas for Gaussian variables, decorrelation by whitening implies independence, this is not necessarily the case for non‐Gaussian variables. Since the independent components ICA is looking for need to be at least decorrelated, that is, orthogonal to each other, PCA can however deliver a good starting point. As we discussed in Section II.A, the decorrelated variables provided by PCA will, however, suffer from rotational ambiguity, i.e there are still an infinite number of possible rotations of this decorrelated set of vectors possible, and PCA thus offers a “space” of possible solutions rather than a unique solution. The stronger requirements of ICA resolve the issue of rotational ambiguity, and the additional information provided by the higher order statistics can be used to retrieve the true independent sources (De Lathauwer, De Moor, & Vandewalle, 2000; Hyvärinen, 2013), that is, find a unique solution/rotation.

However, some considerations should be taken into account. Statistical independence is a strong property with potentially infinite degrees of freedom (Hyvärinen, 2013) (as it requires checking all possible combinations of all linear and nonlinear functions). Whitening the data reduces the degrees of freedom by restricting the search to orthogonal matrices, but the search space is still very large. To find a unique solution, most ICA algorithms operate in an iterative way using local optimization methods, and aim to increase an objective function. While a global optimum for the ICA problem exists, there is a chance that the algorithm gets stuck in a local minimum. For this reason, ICA often requires multiple runs with random initializations and a consequent search for consensus over these runs (e.g., using ICASSO (Himberg & Hyvärinen, 2003)). Additionally, most ICA algorithms cannot identify the actual number of source signals, and therefore require the user to specify the number of expected components beforehand (which further restricts the search space). Finally, unlike PCA, ICA does not provide an inherent ordering of the source signals or components it found.

#### 2 Application to IMS

Siy et al. (2008) applied the fastICA algorithm to MALDI IMS data of mouse cerebellum and compared the results to those of PCA and NMF. Overall, the components retrieved by ICA showed less noise than those obtained by PCA, both in the component images as in the pseudospectra. Contrary to PCA, much of the noise, including the baseline, was separated by ICA into a single component. The pseudospectra contained fewer negative values than those in PCA, and spectra and expression images were comparable to those of NMF (see also Section II.E). Hanselmann et al. (2008) also applied the fastICA algorithm to MALDI IMS measurements and compared its results to PCA, NN‐PARAFAC (which can be considered a type of NMF), and a newly introduced method, pLSA. The approaches were tested in both simulated and real IMS data. In the simulated data, ICA extracted fewer components than NN‐PARAFAC and pLSA, while in the real IMS data ICA returned component images with lower contrast than those of NN‐PARAFAC and pLSA. One critique was that its spectra contained a relatively high number of negative peaks, which are difficult to interpret. These components did not clearly delineate the underlying tissue or cell types, an aspect which was more readily apparent from NN‐PARAFAC and pLSA, both of which only give non‐negative components. In recent work, Gut et al. (2015) have compared pharmaceutical tablets of known composition, using PCA, ICA, NMF, and MCR‐ALS. Similar to previous studies, Gut et al. noted that the components provided by PCA are difficult to interpret. ICA provided the most accurate method to extract appropriate contributions of chemical compounds. In contrast, NMF and MCR‐ALS were better at distinguishing semiquantitative information in heterogeneous tablets due to their non‐negativity constraints. In our own research, we have used a variant of ICA, namely group ICA (GICA), to discover differences between different MALDI IMS experiments (Verbeeck, 2014b). GICA was originally developed for the differential analysis of fMRI data (Calhoun, Liu, & Adalı, 2009), and entails two pattern extraction and dimensionality reduction steps with PCA, one at the individual experiment level, and one at the level of the full dataset, after which ICA is performed. This two‐step pattern extraction improves retrieval of signals at the single experiment level, while reducing the size of the data at the level of the full dataset, making the analysis faster and less memory intensive. Similar to Siy et al. (2008), the resulting pseudospectra contained fewer peaks and contained fewer negative values than those obtained with PCA, while obtaining spatial expression images with very little noise. In an artificially generated dataset, GICA allowed near‐perfect retrieval of the original signals.

### D Maximum Autocorrelation Factorization

Maximum Autocorrelation Factorization (MAF) was originally proposed by Switzer and Green (1984) as an alternative method to PCA to analyze satellite and other multivariate spatial data. MAF has been used as a multivariate analysis technique in SIMS on several occasions (Tyler, Rayal, & Castner, 2007; Henderson, Fletcher, & Vickerman, 2009; Park et al., 2009; Hanrieder et al., 2014), but has been used only sparsely in MALDI IMS, primarily in combination with other data analysis techniques (Jones et al., 2011; Balluff et al., 2015). Stone et al. (2012) used the minimum noise fraction (MNF) transform, a related technique.

#### 1 Principle

Similar to PCA and ICA, MAF aims to decompose the original data as

$$D=S_{\text{MAF}}L_{\text{MAF}},$$

where $S_{\text{MAF}}$ and $L_{\text{MAF}}$ are the scores and loadings, respectively. MAF aims to find the transformation that maximizes the autocorrelation between neighboring observations, that is, neighboring pixels in images. This is under the assumption that signals of interest will exhibit a high autocorrelation, whereas most noise sources will exhibit much lower autocorrelation (Larsen, 2002), and will consequently have a lower ranking in the extracted components. The autocorrelation is generally calculated by taking the correlation between an image and that same image offset by a number of pixels, for example, one pixel diagonally (Henderson, Fletcher, & Vickerman, 2009). Switzer and Green (1984) originally proposed a horizontal offset of one and a vertical offset of one, followed by a pooling of the two variance‐covariance matrices resulting from each individual offset. The resulting covariance matrix is then used as the input for a PCA analysis, and the extracted PCs are the MAF factors, which are ranked from high autocorrelation to low autocorrelation. Due to the way it is formulated, MAF has the advantage that it can incorporate both spatial information (through the autocorrelation) and spectral information (correlation between mass channels) into a single analysis, contrary to other factorizations that consider these separately. As the technique uses PCA, the number of components does not need to be selected prior to the analysis. Larsen (2002) have shown that MAF analysis is equivalent to the Molgedey‐Schuster ICA algorithm (Molgedey and Schuster, 1994).

When applied to remotely sensed spectrometer data with 62 spectral channels, MAF performed much better than PCA in separating interesting signal from noise (Larsen, 2002). Furthermore, components retrieved by MAF tend to have a more intuitive ordering (components of interest ranked higher, noise ranked lower) compared with those retrieved by PCA (components of interest interspersed with noisy components). PCA's suboptimal result is most probably due to some of the interesting components having high autocorrelation (=high ranking in MAF), but having lower variance (=low ranking in PCA) than some of the noisy components. By incorporating additional structural information into the model, Larsen (2002) pose that MAF achieves a better ordering and compression of the data.

#### 2 Application to IMS

Tyler et al. (2007) compared MAF to PCA in analyses that used different types of scaling on SIMS data, acquired from several types of samples. Unlike PCA, MAF is scaling independent, as the correlation calculated between an image and an offset of that same image will cancel out any (linear) scaling applied to the imaging data (Tyler, Rayal, & Castner, 2007; Larsen, 2002). Overall, MAF produced the best result, achieving better dimensionality reduction, offering the highest image contrast, and recovering important spectral features. Furthermore, MAF was able to retrieve subtle features that were lost with PCA, and could not be visualized in individual ion images. Park et al. (2009) reported comparable findings. Although MAF outperforms PCA with different scalings, Tyler et al. (2007) did report that MAF was computationally more intensive, taking two to five times longer to compute for their data. Furthermore, MAF typically requires more computer memory as the autocorrelation needs to be computed on the full image, resulting in a matrix of size m×m, where m is the number of pixels in the image. With increasing sizes of IMS images, this requirement can become prohibitively large.

However, Nielsen (2011) proposed the use of chunking (splitting up the image in multiple sections) to counter this issue. Furthermore, Tyler et al. note that PCA using root mean scaling and shift variance scaling (which was inspired by MAF) offers comparable results, and is less computation and memory intensive. In contrast, Kono et al. (2008) did not retrieve any meaningful results using MAF, due to the fact that the algorithm did not converge.

**Figure 5:**
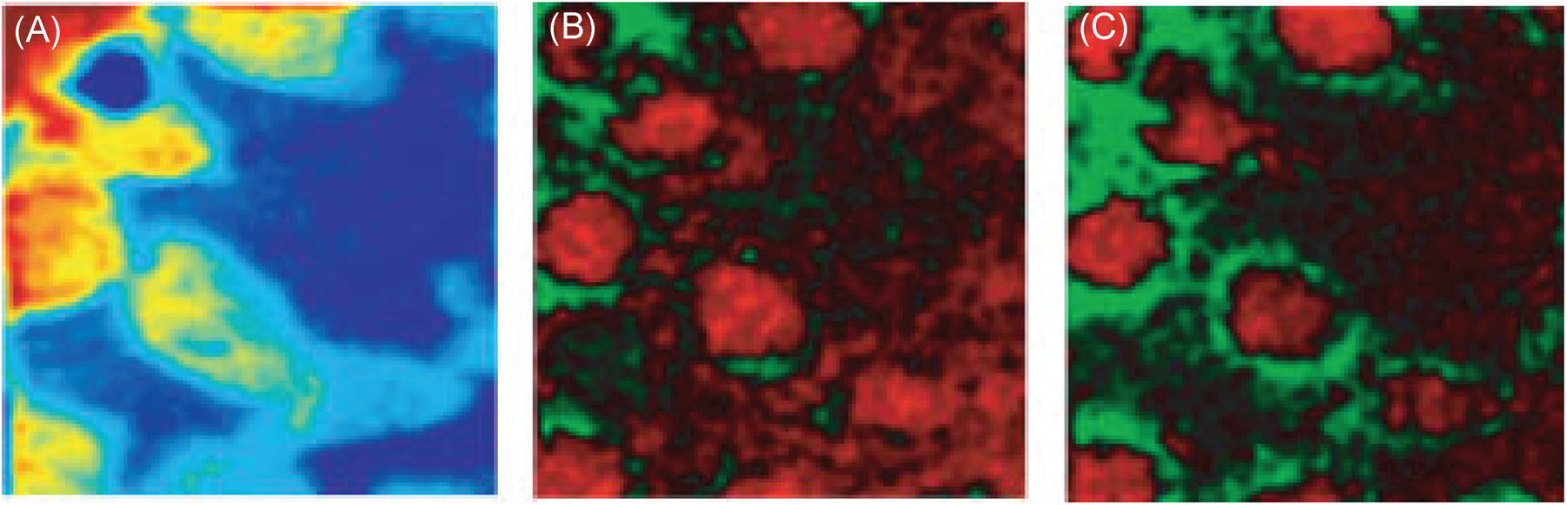
A comparison of PCA and MAF results. Original caption: *Scaled, smoothed images of HeLa cells showing (**a**) the total ion image; (**b**) PC 6; and (**c**) MAF 4. MAF captures the distinction between inner and outer cellular regions more clearly than PCA. Comparison with the total ion image shows the additional information available following multivariate analysis*. Source: Henderson et al. (2009), Figure 3, reproduced with permission from John Wiley & Sons. MAF, maximum autocorrelation factorization; PCA, principal component analysis. [Color figure can be viewed at wileyonlinelibrary.com]

Henderson et al. (2009) found that MAF outperforms PCA specifically in data with a low signal to noise ratio: the autocorrelation allows MAF to better filter out noise and extract genuine signal, as is demonstrated in Figure 5. They also investigated the effects of spatial resolution on MAF results by summing up neighboring pixels, which can be done to improve signal intensity in low intensity datasets. From this comparison, they concluded that MAF should not be used in situations where spatial resolution is important, such as where the size of the sample features of interest is approaching the spatial resolution of the measurement, as in such cases there will remain little autocorrelation to exploit. This observation holds true for most techniques that rely on the local pixel neighborhood to filter noise and improve analysis results (see e.g., Section III.E on the incorporation of spatial information into the clustering of IMS data). In such resolution‐strained cases, the spatial context is less informative since there are fewer local measurements available to describe the same underlying biological pattern. In that case, techniques that do not use neighborhood information have the advantage (by exclusively looking at the chemical information to make their assessment), while local neighborhood‐based techniques run the risk of filtering out such signals as noise (due to insufficient local representation of a genuine biological or sample‐born pattern). Ideally, a combination of both types of analysis would be used.

Stone et al. (2012) have used the minimum noise fraction (MNF) transform (Green et al., 1988) in the analysis of coronal murine midbrain sections. MNF has ties to MAF, and aims to incorporate spatial noise estimation into hyperspectral data analysis. In the original work on MNF, Green et al. (1988) used the shift difference MAF approach as a method to estimate the spatial variance in the data. However, more complex spatial relationships can be plugged into the analysis as well if desired. Stone et al. showed extraction of spatially coherent components from murine tissue data using MNF with a relatively simple local linear fit, outperforming what could be achieved with PCA. Furthermore, when using the extracted MNF components to perform HC (see Section III.B), the results demonstrated good segmentation of the image into clusters with high spatial coherence.

### E Non‐negativity Constrained Matrix Factorizations

A disadvantage of the previously discussed factorization methods is that they will often have negative peaks in the pseudospectra or negative values in their spatial expression images. Such negative peaks are often difficult to interpret, since there is no straightforward physical meaning to negative ion intensity counts. Nevertheless, these negative values can be relevant, for example, they can signify a downregulation of the peak compared to the mean or compared to other components. However, they can also be the result of mathematical constraints that do not necessarily line up with the physical constraints of the underlying measurement process or sample reality. For example, PCA of IMS data tries to capture as much variation as possible in each component. Although this objective is useful for data compression, it does not limit the results to follow the physical reality of mass spectrometry (i.e., allowing only zero or positive ion counts). As a result, negative values are not uncommon (since the data are often mean‐centered) and interpretation from a mass spectrometry viewpoint becomes more difficult.

In order to avoid the issue of difficult‐to‐interpret negative values, researchers have adopted the use of non‐negativity constrained matrix factorization. As the name suggests, it enforces the resulting components to contain only non‐negative (i.e., positive and zero) values. Non‐negativity is a useful constraint in a host of applications where negative values are absent from the native measurements, including facial recognition (Lee & Seung, 1999), audio signal separation (Wang, 2010), and analysis of gene expression data (Kim & Park, 2007). There are various approaches to the problem of non‐negativity constrained matrix factorization, with origins in different fields and differences in underlying ideas and implementation. Of these, multivariate curve resolution (MCR) has been predominantly used in SIMS research, whereas NMF has been mostly applied in MALDI IMS research.

#### 1 Multivariate Curve Resolution

MCR was originally introduced in analytical chemistry in the 1970s through the work of Lawton and Sylvestre as Self Modeling Curve Resolution (SMCR) (Lawton & Sylvestre, 1971). The goal was to extract the concentrations and pure representative spectra of the individual components in two‐compound mixtures measured by UV spectroscopy. SMCR is based on the multicomponent Lambert‐Beer law and aims to decompose the observed mixture data matrix D of size m×n as

(4) $$D=CS^{T}+E,$$

where C (of size m×k) and $S^{T}$ (of size k×n) contain, respectively, the pure concentration profiles and pure spectra of the k species of the unknown mixture (Ruckebusch & Blanchet, 2013), and E is a matrix containing error terms. Lawton and Sylvestre imposed a non‐negativity constraint to ensure that the obtained solutions were limited to physically realizable pure‐component abundances and spectra. The technique was later expanded to mixtures with more than two components (Borgen & Kowalski, 1985; Borgen et al., 1986). Tauler (1995) subsequently developed multivariate curve resolution by alternating least squares (MCR‐ALS), which has since become the most widely adopted MCR algorithm (Ruckebusch & Blanchet, 2013). One of the reasons for the popularity of the alternating least squares approach to MCR, is the ease with which constraints can be integrated into the calculation.

Like many bilinear decomposition methods, the solutions provided by MCR are not unique. The retrieved components suffer from rotational ambiguity (Albuquerque & Poppi, 2015) (see Section II.A.6) and, similar to ICA, they are not ranked (sometimes referred to as permutation ambiguity (Albuquerque & Poppi, 2015)). A third type of ambiguity, not previously discussed, that is present in many decomposition methods including MCR, is intensity ambiguity. Intensity ambiguity arises from the fact that any nonzero scalar multiplication on the spectral side can be compensated by a division with the same scalar on the spatial expression side, and vice versa. The result is that the spatial expression images and pseudospectra generally have different relative scales, and thus cannot be directly compared (in terms of absolute values) between components. This becomes especially important in a quantitative setting where the resulting components are used to estimate concentrations in the sample. Combined with the fact that absolute quantitation is a nontrivial challenge in many forms of IMS (e.g., due to occurrence of matrix and ion suppression effects (Heeren et al., 2009)), an aspect that goes beyond the scope of this review, one might be skeptical about the use of such methods for quantification in an IMS context. However, it should be noted that even with intensity ambiguity, many factorization methods can be very useful in delivering at least relative quantitative insight into underlying distribution patterns and correlations.

In light of the stated ambiguities, researchers using MCR often aim to narrow the range of potential solutions by supplementing the non‐negativity constraint with additional constraints. These constraints can be based on physical properties or prior information on the system at hand, such as kinetic constraints (De Juan et al., 2000). Other approaches narrow the search space by using prior information to limit the number of components, for example, the number of chemical compounds known to be present in certain regions of a Raman spectroscopy image (De Juan et al., 2008). The iterative alternating least squares approach devised by Tauler (1995) makes it straightforward to introduce such additional constraints into the calculations.

MCR has been used in a host of chemometrics applications, including various types of hyperspectral imaging, such as Raman Spectroscopy (Gallagher et al., 2004) and near‐infrared imaging (Gendrin et al., 2008). MCR has only recently started being employed in MALDI and DESI‐based imaging (Rao et al., 2013; Jaumot & Tauler, 2015; Gut et al., 2015). However, together with PCA, it is one of the most‐used techniques in the analysis of SIMS imaging, and has a large body of work dedicated to it. Some of the earliest applications of MCR in SIMS imaging were done at the Sandia National Laboratory and General Electric using the AXSIA toolbox, which was originally developed for the analysis of energy dispersive X‐ray spectroscopy (EDS) data. In 2004, Ohlhausen et al. (2004) analyzed SIMS imaging data of a MEMS device using MCR. The data was Poisson scaled and resulting spectra and images were easy to interpret, with the technique being able to retrieve small and unexpected peaks from the sample. Smentkowski et al. (2004) analyzed polymer samples, wherein MCR analysis allowed identification of two metallic contaminants that were not apparent using the standard data analysis protocol. Gallagher et al. (2004) developed a sequential MCR‐ALS that progressively expands to include additional factors, allowing very controlled integration of constraints, per factor and element. They presented a robust algorithm for initialization in SIMS imaging and Raman spectroscopy data. More recently, Hook et al. (2015) have used such an iteratively expanding approach to parallellize and speed up the calculation of MCR‐ALS in large SIMS imaging datasets, reducing calculation time, and memory requirements. Wagner et al. (2006) compared PCA and MCR with different preprocessing in SIMS data of a thin film of poly(methyl methacrylate) spin‐cast onto a silicon wafer substrate. PCA and MCR showed comparable image contrast, but MCR retrieved spectra the most reliably. Poisson scaling showed the best results preprocessing‐wise (over image normalization and unnormalized). Tyler (2006) compared MAF, PCA, and MCR in simulated SIMS data. Here, MAF achieved the best image contrast, followed by PCA and finally MCR. However, MCR achieved the best correlation with the original spectra. MCR was initialized using guesses for the component spectra. Yokoyama et al. (2015) compared PCA and MCR in SIMS data of polymer samples, and saw a larger influence/better detection of introduced contaminants (Ar‐ions sputtered onto the sample) by PCA than by (Poisson‐scaled) MCR.

Lee et al. (2008) have compared PCA and MCR with different types of preprocessing (no scaling, TIC normalization, variance scaling, and Poisson scaling) in SIMS imaging data of a PVC‐PC polymer blend. Both PCA and MCR were suitable for identification and quantification of components. Poisson scaling showed marked improvements over other preprocessing methods, allowing PCA to capture the data with fewer components, and MCR with Poisson scaling showing quantification and retrieval of the original spectra.

Lee et al. (2009) compared PCA to MCR on SIMS data of human hairs, using different scaling methods (no scaling, Poisson scaling, and binomial scaling). They found relatively good robustness to scaling for the MCR results (with binomial scaling providing the best scaling to deal with detector saturation). In SIMS data of a PP/PE polymer, Miyasaka et al. (2008) found good retrieval of the original PE and PP spectra using MCR. Unlike Lee et al., Miyasaka et al. reported high influence of scaling on the MCR results, with autoscaling giving the most accurate results. Gelb et al. (2015, 2013) developed a maximum a posteriori approach for multivariate analysis, rooted in Bayesian probability theory. It specifically considers the statistical characteristics of SIMS data, namely its Poisson distribution, and detector saturation. When compared to standard MCR‐ALS, their method showed better retrieval of the original signal from data simulating detector saturation. This method allows straightforward extension to include nonlinearity and other IMS‐specific properties, and facile incorporation of a priori knowledge into the analysis. In Gelb et al. (2013), simulated annealing was used to solve the optimization problem rather than ALS, as it allows more flexibility on the choice of the model. Aoyagi et al. (2011, 2012) used MCR with Poisson scaling in SIMS data of mouse skin to determine the concentrations of hair restoring compounds and their effectiveness on hair growth. Smentkowski et al. (2005) applied MCR to SIMS data of a mixed oxide/metal sample, using Poisson scaling and initialization by PCA+Varimax. Positive and negative mode ion images were concatenated and analyzed together. Later, Smentkowski et al. (2007) showed the application of MCR to 3D SIMS data of a nanoporous SiO2 film on a GaAs substrate. In order to make the analysis feasible, the data was reduced by mass‐binning prior to analysis. In recent work, Keenan et al. (2015) proposed a framework for application of MCR‐ALS to SIMS data. It allows the integration of preprocessing steps such as scaling directly into the MCR‐ALS algorithm, while also incorporating MAF‐like error estimation into the analysis. It also allows, for example, constraints on the angle between components, giving preference to components that are more orthogonal to each other. Aram et al. (2015) developed an efficient approach to MCR‐ALS that incorporates spatial correlation directly into the analysis, and they demonstrate this in SIMS data of a metabolite mixture. The algorithm uses 2D Gaussian basis functions to incorporate this information into the modeling task, and it employs the Shannon sampling theorem to determine the width of the basis functions. For high‐resolution images, the speed of the calculation can be increased at the cost of accuracy by choosing a larger width for the basis functions.

**Figure 6:**
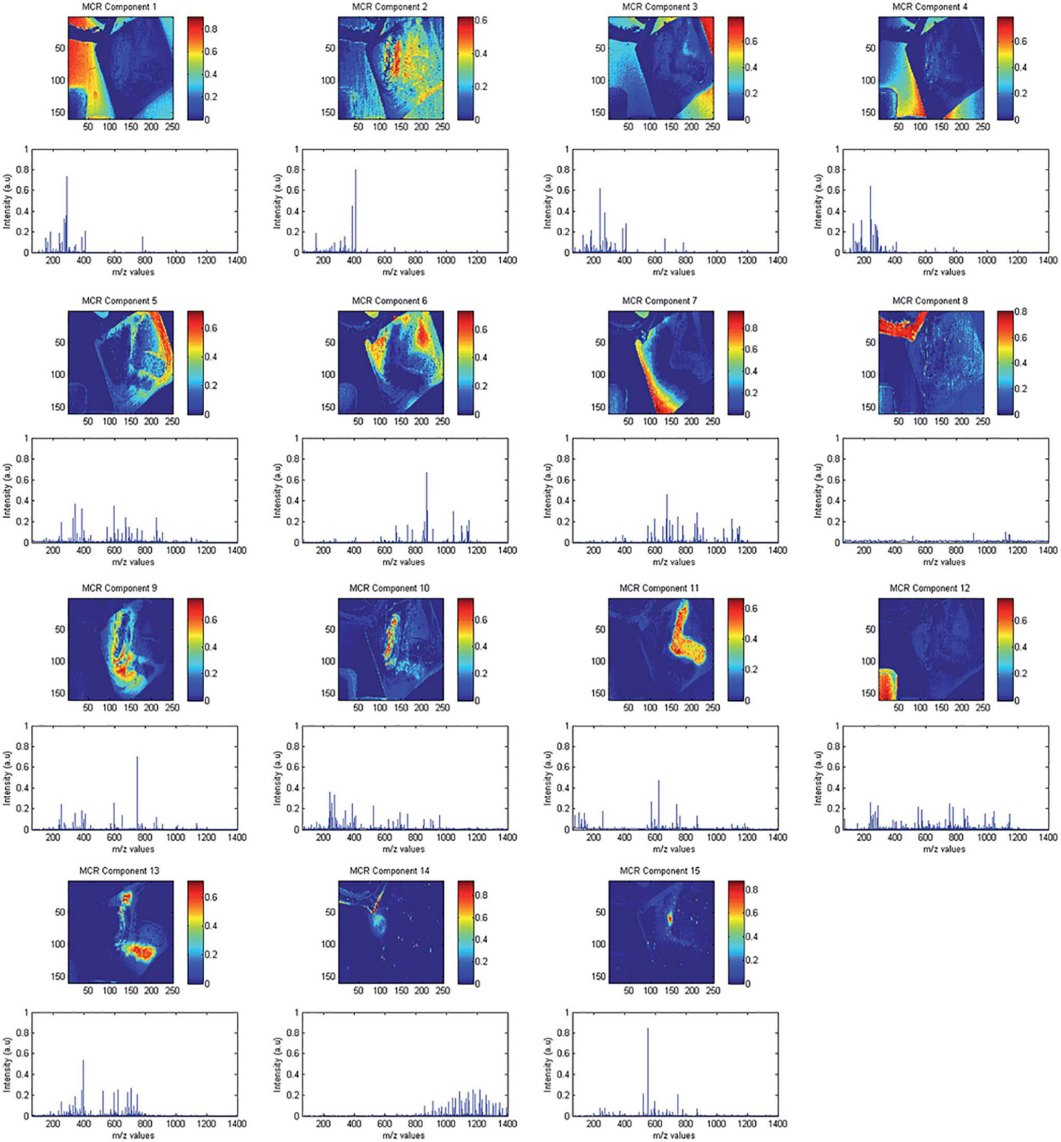
Example of MCR‐ALS applied in NIMS data. Original caption: *MCR‐ALS results of the microbe dataset. Distribution maps after refolding and MS spectra of all resolved components*. Source: Jaumot & Tauler (2015), Figure 2. Adapted with permission of the Royal Society of Chemistry. MCR‐ALS, multivariate curve resolution by alternating least squares; NIMS, nanostructure‐initiator mass spectrometry. [Color figure can be viewed at wileyonlinelibrary.com]

**Figure 7:**
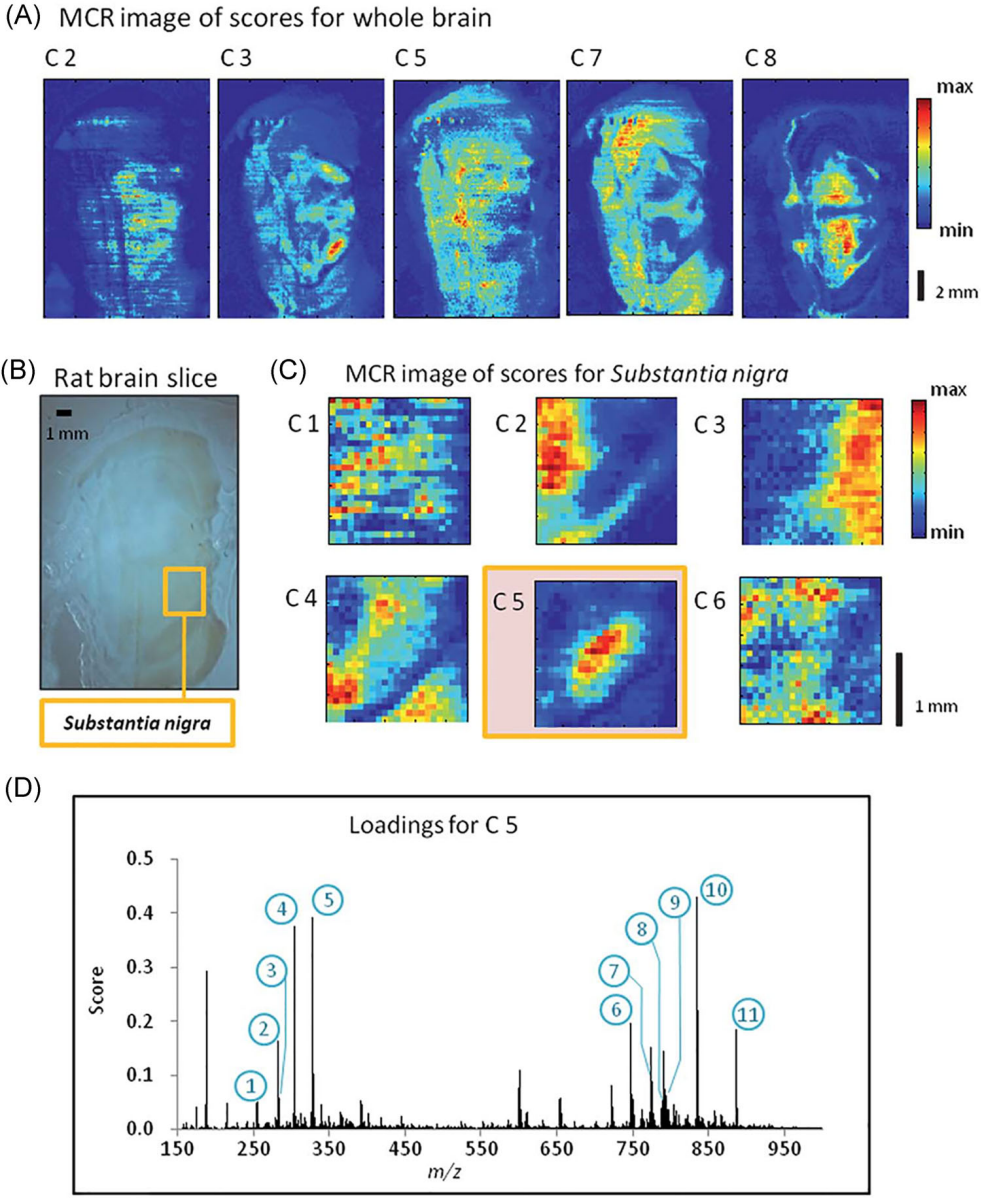
Example of MCR applied in DESI data. Original caption: *Progressive MCR analysis of rat brain slice. (**a**) MCR analysis of the entire image with 8 components. MCR components 2, 3, 5, 7, and 8 show the presence of distinct brain structures. (**b**) Optical image of original brain slice showing the location of the substantia nigra. (**c**) MCR analysis of the substantia nigra part of the image with 6 components. Component 5 picks out the brain region clearly. (**d**) Plot of the loadings scores for component 5 from the MCR analysis of the image for the substantia nigra region. The 11 numbers refer to tentatively identified peaks, which are presented in table 1*. Source: Rao et al. (2012), Figure 7. Reproduced with permission of the Royal Society of Chemistry. DESI, desorption electrospray ionization; MCR, multivariate curve resolution. [Color figure can be viewed at wileyonlinelibrary.com]

With regard to non‐SIMS imaging applications, Jaumot et al. (2015) applied MCR‐ALS to a nanostructure‐initiator mass spectrometry (NIMS) microbial dataset by Louie et al. (2013), results of which are shown in Figure 6. They also applied MCR‐ALS to a mouse lung MALDI Orbitrap IMS dataset by Marko‐Varga et al. (2011), available from the openMSI platform (Rübel et al., 2013). The data were baseline corrected and TIC‐normalized. The resulting components were straightforward to interpret, showing good spatial contrast. The authors reported that the number of components was difficult to estimate, and for this reason the analysis was repeated multiple times using different component numbers. Rao et al. (2012) have used MCR in the analysis of DESI data of intact proteins on biomaterial surfaces and in rat brain tissue, as well as on data from in‐situ surface tryptic digested proteins on a biomaterial surface (Rao et al. 2013). MCR showed promising results for unraveling complex intact protein and protein digestion spectra. An example of MCR applied to DESI IMS is shown in Figure 7.

#### 2 Non‐negative Matrix Factorization and Positive Matrix Factorization

In parallel with the development of MCR‐ALS, Paatero and Tapper (1994) developed positive matrix factorization (PMF) as a method to analyze environmental data (e.g., air pollution; with analysis aimed at assigning contributions of different gases). PMF was later applied for curve resolution (Xie, Hopke, & Paatero, 1998) in NMR spectra. One of the differences with MCR‐ALS is that PMF relies on maximum likelihood to obtain its component solutions (Ruckebusch & Blanchet, 2013). Lee and Seung (1999) popularized the technique as NMF, and focused on its use toward parts‐based learning. This was illustrated in facial recognition, where NMF decomposed facial images as summations of different noses, eyes, mouths, and so forth, while PCA decomposed facial images as different “eigenfaces” and required summation of eigenfaces to construct the original face. PCA resulted in a decomposition that was generally less intuitive and harder to interpret than the parts‐based approach provided by the NMF. Furthermore, the results of the NMF algorithm tended to be sparser than those obtained through PCA, aiding in their interpretability.

PMF and NMF differ primarily in the objective function and update rules employed by the algorithm. NMF uses a relatively straightforward multiplicative update rule (Lee & Seung, 1999; Gendrin et al., 2008) that is guaranteed to converge to a local maximum, although this can be slow (Lee & Seung, 1999). An interesting aspect of this rule is that, with positive initialization, and S C (which contain the pseudospectra and spatial expressions, similar to (4)) remain positive in subsequent iterations, and thus require no additional constraints. PMF, on the other hand, uses an alternating least squares approach with a squared error objective function (Paatero and Tapper, 1994; Gendrin et al., 2008). It gives variables with a high precision (low standard deviation) a higher impact in the error function, ensuring robustness to noise. The optimization task is solved using a conjugate gradient algorithm, iteratively applying non‐negativity constraints during each optimization step.

**Figure 8:**
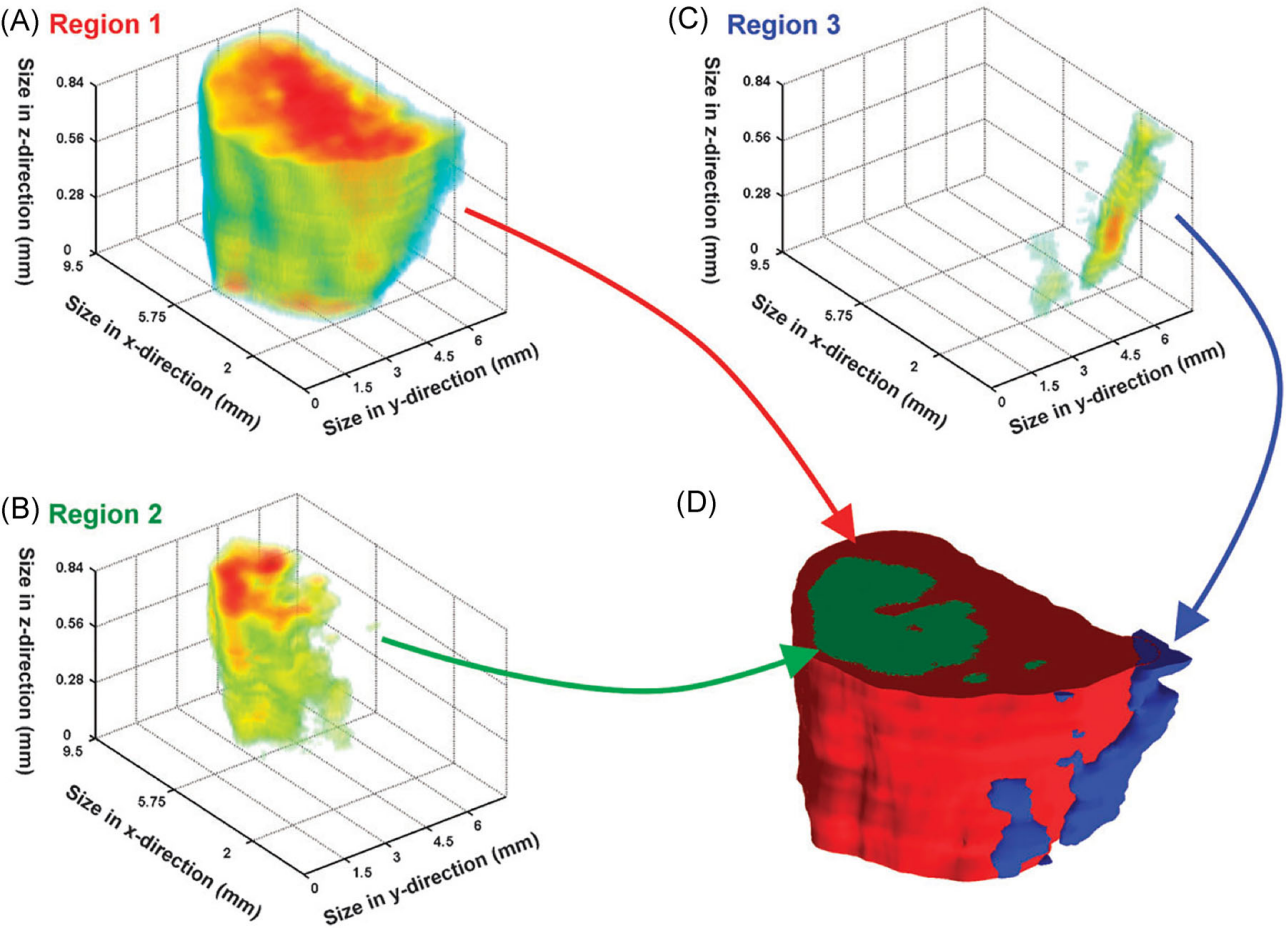
Example of NMF applied to NIMS data. Original caption: *Multivariate analysis of the 3D tumor model. Non‐negative matrix factorization (NMF) was used to identify regions within the 3D dataset. (**a**) The 3D volume defined by component 1/region 1 represents the bulk of the tumor. (**b**) Component 11/region 2 lies within the interior of the tumor. (**c**) A region along the edge of the tumor was identified by component 15/region 3. (**c**) The 3D intensity data for each region can be used to create a 3D space‐filling model, where each color represents a different region within the dataset (region 1: red; region 2: green; region 3: blue)*. Source: Reindl et al., 2011, Figure 3. Reproduced with permission of the Royal Society of Chemistry. NIMS, nanostructure‐initiator mass spectrometry; NMF, nonnegative matrix factorization. [Color figure can be viewed at wileyonlinelibrary.com]

NMF has been used in MALDI IMS on multiple occasions. In the comparison between PCA, ICA, and NMF by Siy et al. (2008), NMF showed similar results to ICA. It exhibited crisp spatial separation with low noise in the score images, but with the added advantage that components were easy to interpret due to non‐negativity. Similar to ICA, NMF directed noise into separate components. In our own work, we have compared the results of PCA and NMF on a synthetic dataset with known composition (Van de Plas, 2010). This experiment showed the potential of NMF to extract the components of the original signal in an easy‐to‐interpret way, particularly when compared with the linear combinations of positive and negative peaks and spatial expressions provided by PCA. Additionally, we demonstrated the use of NMF for the analysis of MALDI IMS data acquired from a sagittal section of the mouse brain. It showed good retrieval of anatomically relevant regions in the brain, where each region had its own pseudospectrum with region‐specific peaks. Reindl et al. (2011) used NMF in the analysis of 3D NIMS data of a mouse mammary tumor, where 30 tissue slices were spatially aligned using correlation‐based registration, resulting in a 4D data array (with x, y, z, and m/z dimensions). Figure 8 shows the decomposition of this dataset. In order to limit the computational load, the 200 most abundant ions were selected for further analysis. The 4D array was unfolded to a 2D matrix, on which NMF analysis was performed. The resulting score images were then reconstructed back to 3D images, resulting in components that are clearly localized in specific 3D regions in the tumor. In the previously mentioned study by Gut et al. (2015), which compares different multivariate analysis techniques on MALDI IMS data of pharmaceutical tablets, NMF and MCR‐ALS showed very comparable results and they were best suited to distinguish semiquantitative information in a relatively heterogeneous tablet. For the NMF and MCR‐ALS analysis, data were scaled using standard deviation. In this study, ICA was the best method to extract the most appropriate contributions of chemical compounds. Xiong et al. (2012b) have used NMF to retrieve lipid profiles distinguishing between tumorous and normal human bladder tissue in DESI imaging data. In recent work by Paine et al. (2016), NMF allowed the identification of metabolites upregulated in tumorous tissue versus healthy tissue in a mouse model of early‐stage ovarian cancer, measured using DESI. In a different DESI IMS application, Tata et al. (2016) have used NMF and PCA to identify regions of necrosis in breast cancer. Boskamp et al. (2017) have used NMF to extract characteristic spectral patterns in formalin‐fixed paraffin‐embedded tissue samples as part of a classification study in lung and pancreatic cancer. Jones et al. (2011) have used both NMF and pLSA in their workflow aggregating results of six different statistical techniques for the analysis of intratumor heterogeneity. In this study, out of all methods applied, NMF and pLSA provided the best description of the heterogeneity, leading to comparable results in the contrast of score images as well as in the retrieval of spectra associated with the tumoral areas. In a later study, Jones et al. (2013) successfully applied NMF to differentiate between microscopically identical and highly heterogeneous tumors. Given the slower calculation speed of pLSA and NMF compared to standard PCA, Jones et al. demonstrated the implementation of these techniques on a GPU. This provided up to a 10‐fold increase in calculation speed over regular CPU implementations using commercially available hardware (Jones et al., 2012b). One of the limiting factors of this approach is that the full dataset needs to fit in GPU memory. This can be a problem for large IMS datasets, but dimensionality reduction can offer a solution there.

**Figure 9:**
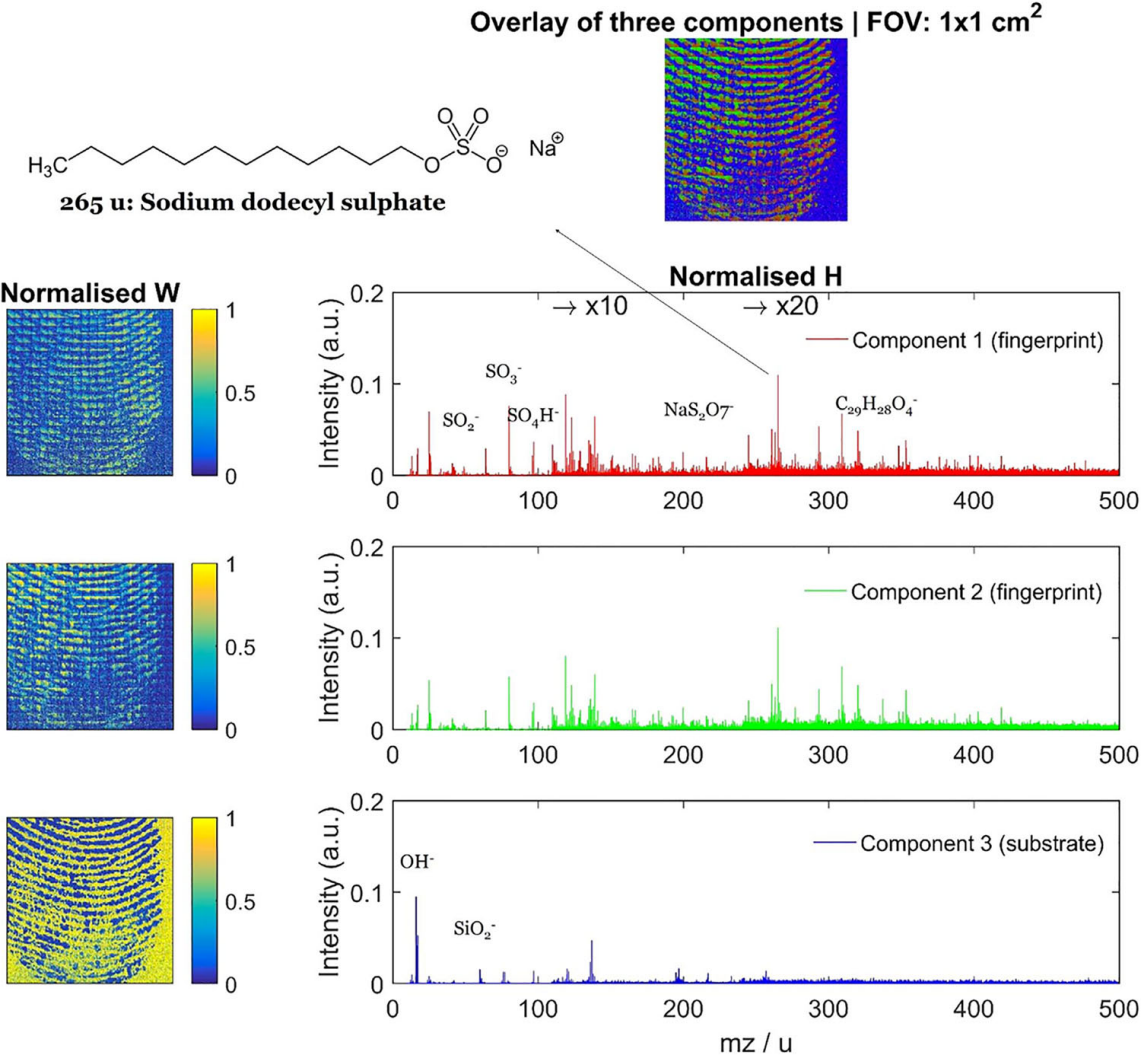
Example of NMF applied to SIMS data. Original caption: *NMF results with three components for the fingerprint dataset. Field of view: 1* *cm × 1* *cm*. Source: Trindade et al. (2017), Figure 4. Reproduced with permission of Elsevier. NMF, nonnegative matrix factorization; SIMS, secondary ion mass spectrometry. [Color figure can be viewed at wileyonlinelibrary.com]

In SIMS imaging approaches, NMF has been applied to identify biochemically distinct signatures of spherular structures in subretinal pigment epithelial deposits (Thompson et al., 2015). As mentioned in Section II.B, NMF has also recently been used in the analysis of high‐spatial resolution 3D TOF‐SIMS data, where a quasirandom sampling scheme was used to compute NMF components (Trindade et al., 2017). This decomposition is shown in Figure 9.

Since its inception, a wide variety of new approaches and algorithms with different objective functions and update rules have been developed to perform NMF (Berry et al., 2007; Cichocki et al., 2009). The newer NMF algorithms often have faster convergence speeds than the original algorithm proposed by Lee & Seung (1999). Furthermore, many of these algorithms are ALS‐based (e.g., the standard NMF algorithm in MATLAB 2016a). Similar to MCR, additional application‐specific constraints have been introduced into NMF, including sparseness (Hoyer, 2004), orthogonality (Ding et al., 2006), and smoothness (Zhe et al., 2006). Conversely, the introduction of weighted alternating least squares to MCR (MCR‐WALS) (Wentzell et al., 2006), which uses a maximum likelihood total least squares approach (Van Huffel & Vandewalle, 1991) instead of standard least squares, allows measurement error information to be incorporated into the modeling process, thus improving MCR's ability to operate for applications with high noise. All these improvements drive NMF and MCR to show increasingly similar characteristics. Albuquerque & Poppi, 2015) have compared NMF‐ALS, MCR‐ALS, and MCR‐WALS in surface‐enhanced Raman imaging spectroscopy data. In low‐noise data, NMF‐ALS produced slightly better results than MCR‐ALS, and superior results to MCR‐WALS, whereas in data with high heteroscedastic noise MCR‐WALS outperformed the other methods. For a comparison of PCA, PMF, MCR‐ALS, and MCR‐WALS, albeit in nonimaging applications, we refer to Tauler et al. (2009).

There are several aspects to consider when using both NMF and MCR. Most non‐negativity constrained algorithms require specification of the number of components prior to starting the analysis. Also, the components retrieved by NMF and MCR are not required to be orthogonal, meaning that the resulting components can be partially overlapping. Furthermore, these algorithms are iterative and do not have a closed form solution. This means that, similar to ICA, they have a chance to get stuck in a local minimum. It is therefore recommended to aggregate results over different iterations using random initializations. Additionally, it is feasible to employ statistical resampling strategies such as bootstrapping (Van de Plas et al., 2015) to get estimations on the errors (De Juan & Tauler, 2003) over multiple runs. It is also possible to guide the method toward a better solution by initializing the algorithm using prior knowledge (De Juan & Tauler, 2003) (e.g., estimates of the expected spectra) or computational methods (e.g., the SIMPLISMA method, which aims to estimate pure components (Windig & Guilment, 1991; Wentzell et al., 2006), or PCA combined with Varimax (Lee et al., 2009)). Donoho & Stodden (2004) have investigated the conditions under which NMF will give a good decomposition into parts. While these conditions are difficult to verify in empirically measured data (where there is no ground truth known), they have shown that in simulated data and under random initialization, difficulties arise when overlap exists between the different parts and common parts are split over different components. In the context of IMS data, this could mean, for example, that when several biomolecular ions are simultaneously present in multiple anatomical structures, these ions could be incorrectly distributed among the different anatomical structures, which is not the expected outcome. Boutsidis & Gallopoulos (2008), however, showed that the original parts can be recovered by using SVD as initialization for the NMF decomposition.

#### 3 Probabilistic Latent Semantic Analysis

Probabilistic latent semantic analysis (pLSA) is a statistical analysis tool that was proposed in the context of automated text analysis (Hofmann, 1999), and was first applied for the analysis of IMS data by Hanselmann et al. (2008). In text analysis, pLSA aims to find the underlying, latent “topics” in documents by analyzing the cooccurrence of words, and it is based on a statistical mixture model called the aspect model, or aggregate Markov model.

The model can be written as

$$p\left(s,c\right)=\;\sum_{t\in T}p\left(t\right)\;p\left(s|t\right)\;p\left(c|t\right),$$

where s is a document, c is a word, t is the latent topic, and T is the set of all latent topics. In IMS, each spectrum can be considered a document, each m/z bin a word, and each pattern within the tissue (e.g., patches of the same cell type) can be seen as a latent topic (Hanselmann et al., 2008). The pLSA method aims to decompose the original data, where each spectrum is considered a mixture of the different tissue types, into the underlying latent variables using the iterative expectation maximization (EM) procedure. The result is a set of probability distributions throughout the tissue for the latent variables or classes (similar to the scores of matrix factorization methods) and the variables that contribute to those classes (similar to the loadings). Given that the resulting components are probabilities, the resulting decomposition is non‐negative and easy to interpret since each peak in the pseudospectrum is directly related to the amount of contribution it gives to that class.

One of the advantages of pLSA is its sound statistical basis. Analysis by pLSA results in a generative model, which means it can be used to generate new spectra on the basis of the learned underlying model parameters. This aids interpretation of the model, and allows verification through for example cross‐validation. It is noteworthy that latent Dirichlet allocation, which is closely related to pLSA and is discussed below in the context of clustering (Chernyavsky et al., 2012), improves and generalizes on the generative model of pLSA. Another important aspect is that it does not assume Gaussian‐distributed noise and signals. Instead, as it is based on word‐counts, it assumes Poisson‐distributed data, which is appropriate for IMS. To deal with Poisson distributions, pLSA uses the Kullback‐Leibler divergence (cross‐entropy) as a measure to fit the model. Ding et al. (2008) have previously noted the parallels between NMF and pLSA, and have shown that NMF using the Kullback‐Leibler divergence measure and pLSA optimize the same objective function. Like NMF, pLSA requires prior selection of the number of components. However, Hanselmann et al. (2008) proposed the use of the AIC (Akaike, 1973) to select the optimal model from multiple models with different numbers of components.

Hanselmann et al. (2008) compared PCA, ICA, NN‐PARAFAC, and pLSA both in simulated IMS data and in genuine MALDI and SIMS data from breast cancer tumors. Although PARAFAC was originally developed as a multiway or tensor decomposition method, it can also be seen as a constrained two‐way PCA model (Hanselmann et al., 2008). Two‐way NN‐PARAFAC is akin to standard NMF, and its solution is generally found using alternating least squares, assuming Gaussian noise (Hanselmann et al., 2008). As mentioned in the section on ICA, NN‐PARAFAC and pLSA were preferred over PCA and ICA due to their interpretability, better component retrieval, and better reconstruction of the original dataset. Furthermore, the components retrieved by NN‐PARAFAC and pLSA were sparser than those obtained with ICA and PCA, further aiding in their interpretability. In the simulated data, pLSA achieved retrieval of the original components comparable to NN‐PARAFAC, but with lower noise in the score images. In the real dataset, pLSA and NN‐PARAFAC provided comparable results. However, one of the main advantages of pLSA components is that they are normalized over all components, and thus the score images are interpretable as probabilities for pixels to belong to one category or another. An earlier application of NN‐PARAFAC for SIMS data can be found in Broersen et al. (2005), where it is compared with PCA and PCA + Varimax, with NN‐PARAFAC results deemed superior over the other techniques.

As mentioned, Jones et al. (2011) have used both NMF and pLSA in their workflow aggregating results of six different statistical techniques for the analysis of intratumor heterogeneity. NMF and pLSA provided the best description of the heterogeneity out of all tested methods. In follow‐up work, Jones et al. (2012b) used GPUs to speed up calculation of pLSA.

#### 4 CX/CUR Matrix Decomposition

Yang et al. (2015) introduced the use of CX/CUR matrix decomposition (Mahoney & Drineas, 2009) for the analysis of IMS data. Similar to SVD and many other matrix factorization methods, CX/CUR matrix decompositions aim to find a low‐rank matrix approximation of the measured data. However, these methods have the property that they construct this low‐rank approximation using original rows and columns from the data matrix as components, rather than using score and loading vectors, which are linear combinations of the original variables. In the case of IMS, CX/CUR decomposition will express its lower‐dimensional representation of the data using a subset of measured ion images and mass spectra, which makes interpretation more straightforward. While the CX/CUR decomposition does not strictly impose non‐negativity, the fact that the measured ion images and spectra it can pull components from are non‐negative (due to the count‐based nature of mass spectrometry measurements), makes the resulting decomposition be non‐negative as well. CX decomposition is used when only the rows or columns are of interest, whereas CUR decomposition calculates both the most relevant rows and columns. Let us take again the original m×n data matrix D, where the rows represent the pixels and the columns represent the spectral (m/z) bins. CX factorization then decomposes D into two matrices C and X, where C is an m×c matrix that consists of c columns originally found in of D, and X is a c×n matrix such that D≈CX.

To select the best columns to construct its low‐rank approximation, the CX/CUR algorithm makes use of the concept of statistical leverage, that is, the algorithm selects the columns that exert a disproportionately large influence on the best low‐rank fit of the data matrix (which can be obtained using SVD) (Mahoney & Drineas, 2009). Compared to SVD, the CX/CUR low‐rank approximation is less accurate, but has a more straightforward interpretation. Furthermore, the algorithm can be implemented efficiently using greedy search algorithms and random vector projections, making this approach typically faster than standard SVD algorithms. Using Apache Spark on a HPC platform, Gittens et al. (2016) demonstrated the potential of parallel computing for speeding up the CX decomposition of a 1TB ion mobility + IMS dataset, achieving decomposition in 1200 sec on 60 nodes (and with NMF and PCA demonstrated on other large datasets).

**Figure 10:**
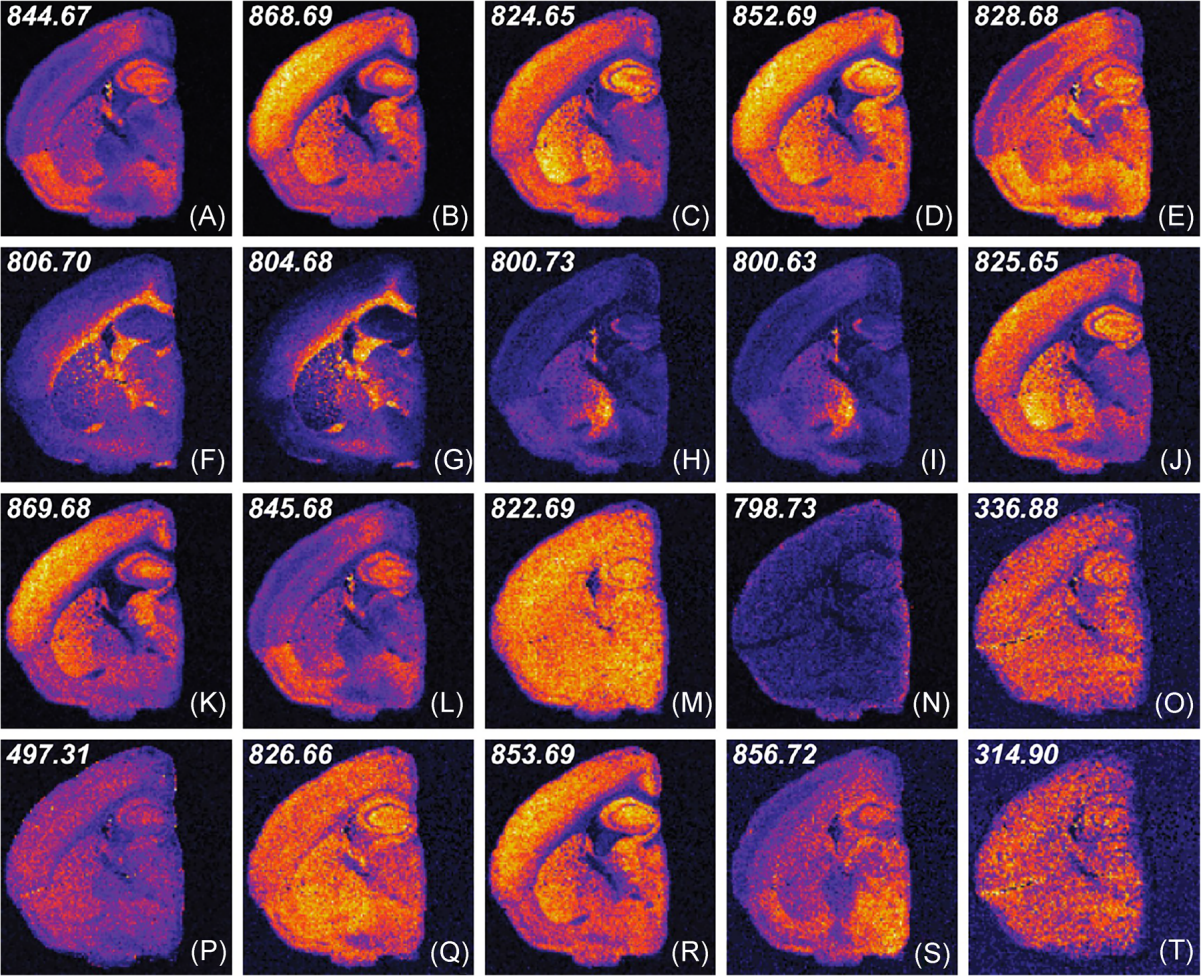
Example of CX/CUR decomposition applied to NIMS data. Original caption: *Ion‐intensity visualization of the 20 most important ions selected via deterministic* CX *decomposition with* k=5 and c=20 *on brain dataset. The distribution of leverage scores is presented in figure 2B [in original paper]. Some of these ions map to distinct regions in the brain. Particular regions of the cortex, pons, and corpus collosum stand out as distinct anatomically identifiable regions. Also in the list are likely background ions and contaminants from the embedding material. Of the 20 ions, little redundancy is present, pointing to the effectiveness of the* CX *approach for information prioritization*. Source: Reprinted with permission from Yang et al. (2015), Figure 3. Copyright 2015 American Chemical Society. NIMS, nanostructure‐initiator mass spectrometry. [Color figure can be viewed at wileyonlinelibrary.com]

Furthermore, CX/CUR matrix decomposition can be employed as a feature selection method, enabling the selection of a subset of ion images or mass spectra that best represent the original data. Yang et al. (2015) show that, using this method, a full IMS dataset could be reconstructed with a 17% reconstruction error using only 20 ions or 40 spectra selected by CX/CUR decomposition. The method can be used as a fast means of gaining quick insight into an IMS dataset with easy‐to‐interpret components, while concurrently reducing the dimensionality of the measurements. Figure 10 shows an example of the CX/CUR decomposition of IMS data.

By selecting original ion images and mass spectra rather than disassembling measurements into underlying component images and pseudospectra, CX/CUR cannot provide the same depth of insight into underlying trends in the data as other methods such as NMF and MCR can deliver. For example, the discovery of cooccurences between ion species is less robust from individually measured mass spectra than it is from pseudospectra that form the consensus across many measurements. Yang et al. (2015) therefore propose the combined use of NMF and CUR/CX decomposition, where the leverage scores computed by CX/CUR decompositions can be used to gauge the informative value of the peaks in NMF components. It is also worth noting that as CUR decomposition bases its statistical leverage scores on subspaces obtained by SVD, its area of applicability will be roughly the same (Mahoney & Drineas, 2009).

#### 5 Dictionary Learning

Harn et al. (2015) introduced a dictionary learning approach, called MOLecular Dictionary Learning (MOLDL), for the analysis of MALDI IMS data. The method considers the Poisson nature of the data, and also uses prior knowledge on the IMS measurements to improve component extraction. First, the method uses prior information on common ion adducts (in microbial data) to construct a dictionary that contains all combinations of such common adducts, and this is used as a basis to decompose the IMS dataset. The MOLDL method restricts the possible decomposition solutions to viable ion + adduct combinations, thus grouping together all peaks in the spectrum that are related to the same molecular species, substantially reducing spurious solutions. Second, MOLDL takes spatial information of neighboring pixels into account to further improve the modeling process. The number of molecular species in the sample does not need to be specified, as this number is uncovered automatically through hyper‐parameter optimization by the algorithm.

MOLDL shows promise for decomposing an IMS dataset into its constituent molecular species, with demonstrations both in a synthetic dataset and in MALDI IMS data of microbial colonies. Due to the fact that the number of possible solutions is strongly reduced, the risk of local minima is also reduced and the method is relatively fast. The authors compare the method with sparse NMF and sparse pLSA, showing better retrieval of the underlying molecular species by the new MOLDL method. One caveat expressed by the authors is that the use of a fixed dictionary can lead to false positives due to real biological but hereto unknown adducts being discarded.

### F Other Methods

Besides the more generally used factorization methods described above, several other decomposition techniques have been applied to IMS data. Gelb et al. (2014) describe a method aimed at including nonlinear interactions between components (e.g., due to ion suppression) into the modeling and decomposition process, by using Taylor series expansion. The method is demonstrated on a synthetic dataset, and solved using alternating least squares. Chen et al. (2014) used (exploratory) factor analysis (FA) in the analysis of a benchmark dataset of air flow‐assisted ionization imaging mass spectrometry (AFAI‐IMS), featuring different types of ink. While FA is related to PCA, it pursues different optimization goals: FA aims to retrieve factors that account for common variance in the data, rather than strive for components explaining maximal variance as is the case in PCA (Suhr, 2005). The authors state that in their benchmark dataset, FA allowed better quantification of the different ink components than PCA. Finally, Signoretto et al. (2011) have demonstrated the potential of using tensor decomposition techniques to infer missing values in IMS data.

## III CLUSTERING

This section provides an overview of clustering techniques, a second widely‐used class of algorithms for exploratory IMS analysis. By grouping or clustering together pixels with similar mass spectral profiles and thus similar chemical content, and by labeling each pixel with the color assigned to its cluster, clustering techniques can use a single false color image to provide a low‐dimensional overview of the high‐dimensional molecular content of an IMS dataset. In IMS, this process is usually referred to as (spatial) segmentation, since clustering on the basis of the spectral domain corresponds to segmentation along the image domain. The use of spatial segmentation is of particular interest in pathology‐directed and clinical applications, where it fits into an area that is sometimes referred to as digital staining. In line with classical pathology, its goal is to delineate medically‐relevant subregions within a tissue sample under study. Alternatively, some applications in IMS have used clustering techniques along the spatial domain, and segment along the spectral domain (spectral segmentation), grouping together ion images with a similar spatial expression. However, most IMS clustering studies focus on spatial segmentation. Unless specified otherwise, our description will therefore default to clustering as grouping together pixels on the basis of their spectral content, rather than grouping together m/z bins on the basis of their spatial distribution.

Where factorization methods had a strong representation in SIMS‐based IMS, clustering techniques seem to have been predominantly applied in MALDI and DESI IMS, and to a lesser extent in SIMS.

### A Intermezzo: Distance Metric and Curse of Dimensionality

Since clustering aims to group together measurements that report similar content, it is important to define what “similar” means exactly. In most clustering algorithms this is accomplished by defining a distance or similarity metric, an expression which grades similarity between measurements in terms of a numerical value (Rokach & Maimon, 2005). These metrics can be customized for the particular data or analysis task at hand. In an IMS context, a distance metric will usually report a distance in molecular content between pixels, with lower values reporting lower distance between the pixels’ mass spectra, or higher similarity. Similarity metrics can be seen as the opposite of distance metrics. A wide variety of distance and similarity metrics have been used in clustering algorithms in the past, including the Euclidean distance, Manhattan distance, Minkowski distance, Pearson correlation coefficient, and cosine similarity, to name a few. Each of these measures has its own advantages and disadvantages, and choosing the right one depends on both the data under study and the target application. We will focus on the Euclidean distance, as it is one of the most commonly used metrics in IMS, and clustering in general (Rokach & Maimon, 2005). The Euclidean distance is given by

$$d\left(p_{1},p_{2}\right)=\sqrt{{\left(\sum_{i=1}^{n}\left(p_{1,i}-p_{2,i}\right)\right)}^{2},}$$

where $p_{1}$ and $p_{2}$ are the mass spectral vectors from two pixels, and i represents the i‐th mass bin of a spectrum. While this is an effective distance measure in low‐dimensional data, in the case of high‐dimensional data (e.g., IMS datasets) the Euclidean distance measure will not perform optimally. Let us assume for a moment that we have two mass spectra with 100,000 m/z bins. Even if we have large intensity changes in 100 m/z bins in one spectrum compared to the other, which can signify an important biological change, this will have only a very minimal effect on the total Euclidean distance. Instead, the accumulated noise over the 99,900 other m/z bins will potentially have a much greater effect on the distance. More formally, the variance in the distance between pairs of points goes toward zero as the dimensionality of the dataset increases. This is a consequence of the “curse of dimensionality” as coined by Bellman (Bellman, 1956; Palmer et al., 2013), which greatly impacts data mining in high‐dimensional data. For this reason, dimensionality reduction techniques, such as those highlighted in the intermezzo on dimensionality reduction, are often applied to IMS data prior to performing clustering analysis. However, as we will see below, there are also clustering methods that have been designed to natively handle such high dimensionality.

### B Hierarchical Clustering

#### 1 Principle

One of the first clustering methods applied to MALDI IMS data was HC (Johnson, 1967; McCombie et al., 2005; Deininger et al., 2008), a clustering algorithm that is widely used in many fields (Jain, Murty, & Flynn, 1999). The result of HC is a hierarchical tree or dendrogram, where each of the leaves of the tree represents a pixel. The tree represents a multilevel hierarchy, where separate clusters on one level are joined together into a more general cluster on the next level. There are two general strategies to construct the dendrogram, namely agglomerative construction and divisive construction. In the agglomerative strategy (a bottom‐up approach), each pixel is assigned its own cluster at initiation. In single linkage clustering, which is one of the ways in which agglomerative HC can be accomplished, the algorithm at each step combines the two clusters whose closest members have the smallest distance between them. It continues this process until the full tree is created and all pixels are together in one single cluster. In the divisive strategy (a top‐down approach), all pixels start within a single large cluster, and at each step the cluster is split into two subclusters, until each pixel has its own one‐pixel cluster.

Once the tree is complete, the data can be queried for a particular cluster set by cutting the dendrogram at a user‐specified number of desired clusters. Alternatively, the dendrogram can be cut by specifying a maximum (or minimum) distance for the clusters to be apart. In that case, the number of clusters will depend on the cluster density and the specific cut‐off distance selected.

#### 2 Application to IMS

An early application of HC on MALDI IMS data can be found in Schwartz et al. (2004). Clustering was performed using the mean spectrum of all spectra collected per tissue sample, effectively clustering the data on a per‐tissue basis. McCombie et al. (2005) applied HC for the spatial segmentation of MALDI IMS data acquired from brain tissue of an Alzheimer's disease mouse model. PCA was used to reduce the dimensionality of the data, primarily to decrease the influence of noise on the clustering process. When using 200 PCs, the HC led to most pixels being members of one large cluster, with all remaining pixels being part of their own single‐pixel clusters, thus resulting in an unsuccessful clustering. The selection of the cut‐off distance is not discussed in detail. When selecting the top five PCs though, the data could be successfully divided into five clusters with multipixel populations for each individual cluster. The authors use PCA/Discriminant Analysis (PCA/DA), a supervised statistical analysis technique, to extract differential signatures between the clusters. This allowed the retrieval of amyloid‐rich regions in the cortex, which were separated by one of the clusters. The authors rightfully comment on the subjectivity of manually preselecting a number of PCs to achieve more telling clusters. An inappropriate choice could lead to incomplete clustering as well as a discarding of useful data features.

**Figure 11:**
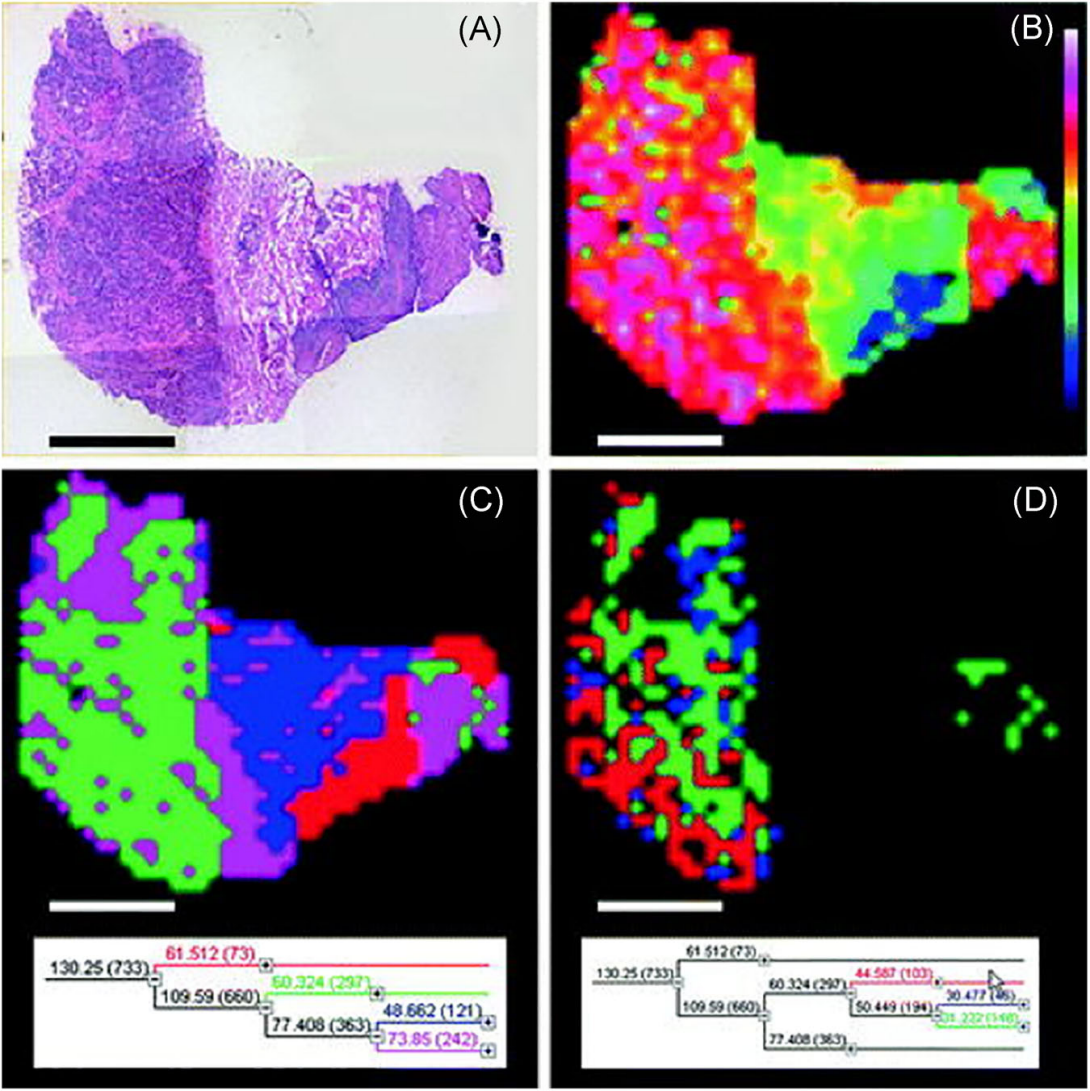
Example of PCA and HC in MALDI IMS data. Original caption: *PCA and hierarchical clustering for a gastric cancer section. (**a**) H&E‐stained tissue section after MALDI imaging measurement. (**b**) Scores of the first principal component show the hot colors in the tumor area. (**c**) Hierarchical clustering: Top dendrogram nodes differentiate tumor (green and magenta) versus nontumor (blue, squamous epithelium in red). (**d**) The dendrogram can be expanded down the tumor node to evaluate the molecular differentiation inside the tumor. This can also be directly correlated with the histology. This workflow enables the fast and concise selection of mass spectra representative for specific tissue states. Scale bar: 2 mm*. Source: Deininger et al., 2010, Figure 22.2. Reproduced with permission from Springer. IMS, imaging mass spectrometry; MALDI, matrix‐assisted laser desorption/ionization; PCA, principal component analysis. [Color figure can be viewed at wileyonlinelibrary.com]

Deininger et al. (2010, 2008) have used HC and PCA in the analysis of MALDI IMS of gastric cancer tissue sections. Figure 11 shows an example from the paper. The HC is performed using the Euclidean distance metric on PCA‐reduced data (70% explained variance), and utilizes the Ward linkage method to construct the dendrogram. This linkage method aims to minimize the “within cluster” variance. Overall, HC analysis shows good overlap with histology, separating tumorous and nontumorous tissue into separate clusters at the higher levels. The authors demonstrate how the constructed HC dendrogram allows for guided exploration of IMS data at the pixel level, by manually selecting the depth to which branches of the tree are expanded. This provides an easy way to explore areas of interest in the tissue to greater depth, for example identifying tumor subareas inside a solid tumor. It should be noted that these IMS‐based subareas do not always correspond to histologically distinct areas. This illustrates that segmentation on the basis of chemical content (through IMS) can potentially reveal tissue delineation not necessarily apparent from microscopy. It hints at substantial potential for IMS to detect phenotypic differences that currently (with non‐IMS modalities) might go undetected. One consideration, though, is that while such manual exploration can be beneficial in the exploration of the IMS data, there is a risk that it takes away part of the objectivity of the analysis, and may lead to confirmation bias. It is therefore preferential to have statistical measures in place to support the obtained final clustering.

Bonnel et al. (2011) use the guided HC approach proposed by Deininger et al. in the analysis of MALDI IMS data of rat brain and FFPE prostate cancer tissue, with trypsin spotted onto the tissue for tryptic digestion of proteins. Data was compared between digested and undigested tissue, as well as between using bottom‐up and in‐source decay strategies for in‐tissue protein identification. After HC, PCA was applied on the clusters of interest, and PC loadings were used to discover discriminating ions between the clusters. El Ayed et al. (2010) used HC with PCA‐based dimensionality reduction for analysis and biomarker discovery in MALDI IMS data of ovarian cancers.

Brulet et al. (2010) employed HC on cluster TOF‐SIMS imaging data, in a study aimed at mapping lipids in colonic mucosa of a cystic fibrosis knockout (KO) mouse model versus wild type (WT). HC was applied on a preselected list of ions generated using a genetic algorithm, and HC analysis allowed clean separation of the KO and WT tissues in two different clusters. Furthermore, k‐means clustering was used on a selected list of peaks, and allowed spatial segmentation of the tissues into regions that showed good agreement with histochemical structure.

Abbassi‐Ghadi et al. (2015) have used HC in a study on the reproducibility of DESI‐MS imaging, applied to esophageal cancer. The tumor was divided into four distinct regions. Four replicates were sectioned for each region, resulting in a total of 16 separate tissue sections. HC was then performed on the mean spectra of each of the four replicates for each quadrant of the tumor, and successfully clustered all replicates into the same cluster.

### C k‐Means Clustering

#### 1 Principle

The k‐means clustering method (Macqueen, 1967; Lloyd, 1982) is one of the most widely used clustering algorithms in data analysis (Rokach & Maimon, 2005), primarily due to its speed and simplicity. In k‐means clustering, the user selects the desired number of clusters, k, prior to starting the analysis. The algorithm is then initialized by selecting k points, in the IMS context these are pixels, which are assigned as the initial cluster centers or centroids. This can be done through random assignment, but is now generally performed using the k‐means++ algorithm (Arthur & Vassilvitskii, 2007), which randomly chooses an initial data point as the first cluster center and then tries to find other points that are “maximally spread out” in the data space. This spreading of the initial clusters has been shown to improve convergence and clustering results. The algorithm then iteratively applies the following steps:

- Calculate the distance of each pixel in the dataset to each of the cluster centers.
- Assign each pixel membership to the cluster whose centroid lies closest.
- Calculate the new cluster centroids by taking the mean spectrum (over all mass bins) of all pixels belonging to the cluster.
- Repeat until the algorithm converges, and pixels no longer change cluster membership.

The k‐means algorithm does not have a unique solution, and the random initialization can influence the clustering results. This can lead to different results each time it is run, and runs the risk that the algorithm can get stuck in a local minimum. For this reason, it is generally recommended to re‐run the algorithm several times and take the consensus over multiple runs. Initialization using prior information, rather than using random values, can also improve clustering results. Similar to the task of determining the number of components in factorization methods, determining the optimal number of clusters can be difficult without prior knowledge. Therefore, it is not uncommon for the clustering process to be performed multiple times with different cluster numbers, enabling selection of a segmentation that is optimal in some sense.

#### 2 Application to IMS

Early applications of k‐means clustering in MALDI IMS data can be found in McCombie et al. (2005) and Muir et al. (2007), both of which applied k‐means clustering to IMS data acquired from mouse brain tissue. Similar to its use in HC, both studies used PCA as a preprocessing method to reduce the dimensionality of the dataset and to remove noisy components in order to achieve a more consistent, smoother spatial segmentation of the data. Jones et al. (2011) used k‐means, directly applied to peak‐picked MALDI IMS data, as one of the techniques in their corroboration analysis. Xiong et al. (2012c) demonstrated the applicability of k‐means clustering to peak‐picked 3D DESI‐MS imaging data, after the data acquired in the different tissue sections were spatially registered using a self‐organizing feature map artificial neural network. This approach allowed the retrieval of biologically relevant volumes from IMS data acquired from a full mouse brain. Brulet et al. (2010) have used k‐means on cluster TOF‐SIMS imaging data. Palmer et al. (2015) successfully used k‐means in the clustering of data after dimensionality reduction and by using less than one percent of the original data (see also Figure 4). Van de Plas et al. (2010, 2008) used k‐means clustering to successfully cluster IMS data after dimensionality reduction using DWT.

**Figure 12:**
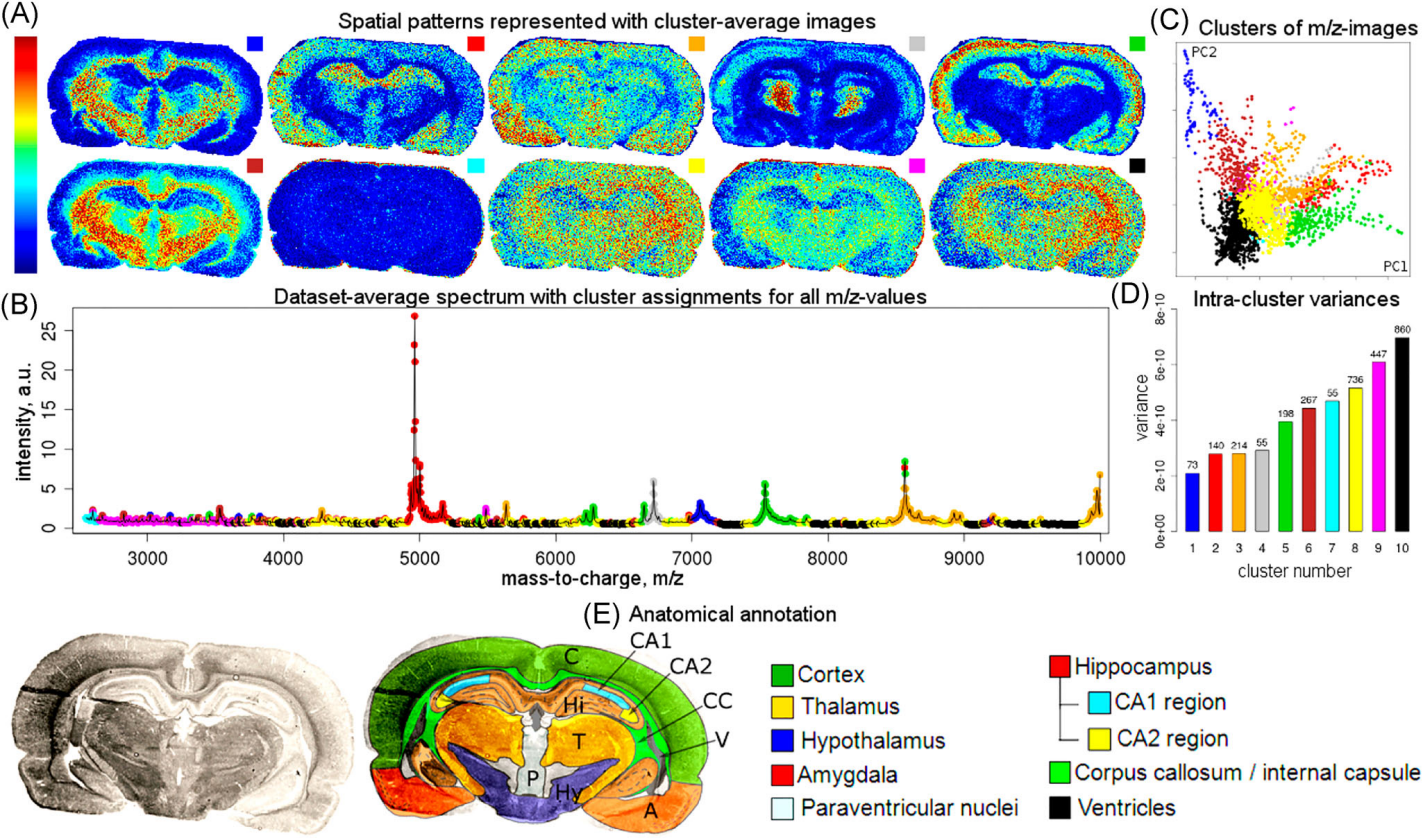
Example of clustering of ion images. Original caption: *Results of the analysis of a MALDI imaging mass spectrometry dataset of a rat brain coronal section, following the proposed approach based on clustering* m/z *images into 10 clusters according to their spatial similarity. (**a**) Cluster‐average images represent detected spatial patterns. (**b**) Data‐set‐average spectrum with assignments of* m/z *values to the clusters; for detailed cluster assignments of all* m/z *values, see Table S2 [in original paper] of the Supporting Information. (**c**) Visualization of* m/z *images in the space of their two first principal components; one dot represents an* m/z *image, and dots are colored according to their cluster assignments. (**d**) Intracluster variances, where the numbers on the top of bars represent the cluster sizes. (**e**) Optical image of the section with anatomical annotation provided. Plots A‐B show the variety of the spatial patterns among* m/z *images and help understand how each* m/z *image looks. Plots **c** and **d** help evaluate the clustering. For results for 5, 15, and 20 clusters, see Figures S3, S4, and S5 [in original paper] of the Supporting Information*. Source: Reprinted with permission from Alexandrov et al., 2013b, Figure 1. Copyright 2013 American Chemical Society. MALDI, matrix‐assisted laser desorption/ionization. [Color figure can be viewed at wileyonlinelibrary.com]

In most IMS applications, clustering has been used to perform a spatial segmentation of the tissue, that is, to group individual pixels together into clusters. However, there are several studies that have taken a different approach by performing the clustering analysis on the ion images and segmenting the m/z bins instead. In this approach, ion images that exhibit a similar spatial expression can be grouped together. Visualization of the cluster centers no longer yields a mean mass spectrum then, but rather a mean ion image per cluster, which allows for straightforward insight into the content of the IMS dataset. Konicek et al. (2012) have used this approach in the analysis of SIMS imaging data of laser‐printed inks. The similarity of different ion images was determined using a correlation‐based distance measure, enabling subclassification and identification of different ink patterns. A similar approach was taken by Alexandrov et al. (2013b), who applied a probabilistic clustering algorithm (a Gaussian mixture model, optimized using EM) to group together ion images in MALDI IMS data, which showed good retrieval of primary trends. An example is shown in Figure 12.

The k‐means clustering method, and more recently its variant bisecting k‐means, have been used in many of the clustering approaches that incorporate spatial information into the segmentation process (see also Section III.E).

#### 3 Bisecting k‐means

Bisecting k‐means (Steinbach, Karypis, & Kumar, 2000) can be considered a hybrid between agglomerative HC and k‐means clustering. In this algorithm, all measurements in the dataset start as members of one large cluster. This cluster is subsequently divided into two subclusters using regular k‐means clustering. At each subsequent step, a subcluster is selected and again bisected or divided into two subclusters using 2‐class k‐means clustering. The process is repeated until the desired number of clusters is reached. In theory, the subcluster to select next should be the one that results in the highest overall intra‐cluster similarity. However, Steinbach et al. (2000) found little difference between methods to select clusters on the basis of similarity, and therefore chose to select the largest remaining cluster in each consecutive step. Trede et al. (2012a) applied bisecting k‐means in the same way as regular HC, repeating the clustering until each cluster has only one sample in it, creating a full hierarchical tree. Bisecting k‐means has been shown to produce better results in document clustering than regular k‐means, and tends to give comparable or better results than agglomerative HC, while having a better run‐time complexity: O(n) vs. $O(n^{2})$ (Steinbach, Karypis, & Kumar, 2000). A substantial advantage of bisecting k‐means clustering over regular k‐means clustering is that the clustering process does not need to be re‐run if more (or fewer) clusters are desired.

### D High Dimensional Data Clustering

#### 1 Principle

High dimensional data clustering (HDDC) is a clustering technique that was developed by Bouveyron et al. (2007) and that aims to improve on the issues related to using Gaussian mixture model (GMM)‐based clustering on high‐dimensional data. Many popular clustering algorithms, including k‐means, assume an underlying Gaussian mixture model (McLachlan & Peel, 2004; Bouveyron et al., 2007). These GMM‐based methods aim to model the observed data as generated from a mixture of a finite number of Gaussian distributions with unknown parameters, which are often estimated using the EM algorithm (Dempster, Laird, & Rubin, 1977). GMM clustering can be considered a generalization of k‐means clustering that incorporates information about the variance and covariance of the data into the clustering process (Press et al., 2007). While k‐means implicitly assumes clusters to have the same variance in each dimension, and consequently expects spherical clusters, GMM clustering allows for the modeling of elliptical‐shaped clusters by taking variance and covariance into account. However, GMMs generally do not scale well when the number of data points is small compared with the number of parameters that need to be estimated, again due to the curse of dimensionality (Bellman, 1956). In order to handle this problem, Bouveyron et al. (2007) assume that the data lies in a subspace that is lower‐dimensional than that of the original data space, and that each class is located around its own class‐specific subspace, a concept that is explored in greater depth in Section IV. These assumptions lead to an adapted version of the GMM, that has a lower number of parameters to be estimated, which, in turn, avoids overfitting of the model. The number of classes in HDDC is automatically estimated using the BIC (Schwarz, 1978), whereas the dimensionality of each class‐specific subspace is estimated using the scree‐test of Cattell (Cattell, 1966) and the parameters of their Gaussian mixtures are estimated using the EM algorithm. In their work, HDDC is shown to outperform standard GMM in clustering of several datasets, and it performs well on a remote sensing hyperspectral imaging dataset consisting of 256 spectral channels.

#### 2 Application to IMS

HDDC was used in spatially‐aware clustering of MALDI IMS data (Alexandrov et al., 2010), obtained from rat brain and human neuroendocrine tumor tissue, and it provided improved results over k‐means clustering. In their follow‐up work, Alexandrov & Kobarg (2011) noted that while k‐means clustering results underperform those obtained by HDDC, the calculation time of HDDC is substantially longer than that of k‐means, which may be prohibitive in some applications. In later work, Alexandrov et al. (2013a) saw minimal differences in clustering results between HDDC and k‐means, which was attributed to improved preprocessing and alignment prior to clustering. It is worth noting that these analyses were performed on peak‐picked data, and thus the dimensionality of the data had been greatly reduced prior to analysis. The difference between HDDC and k‐means on full size full profile IMS data are expected to be larger. Furthermore, although in the preceding work HDDC is used for hard segmentation, HDDC and GMMs natively allow for soft‐segmentation, a topic discussed in Section III.F.

### E Incorporation of Spatial Information

The application of dimensionality reduction prior to performing clustering can remove noise variation from the data and thus improve the smoothness of a spatial segmentation result. However, in many cases a substantial amount of nonbiological inter‐pixel variation will remain, resulting in spatially discontinuous segmentation maps. To address this issue, several approaches have been proposed to smooth spatial segmentation maps. Many of these methods exploit the spatial or neighborhood information available between pixels, also in IMS data.

**Figure 13:**
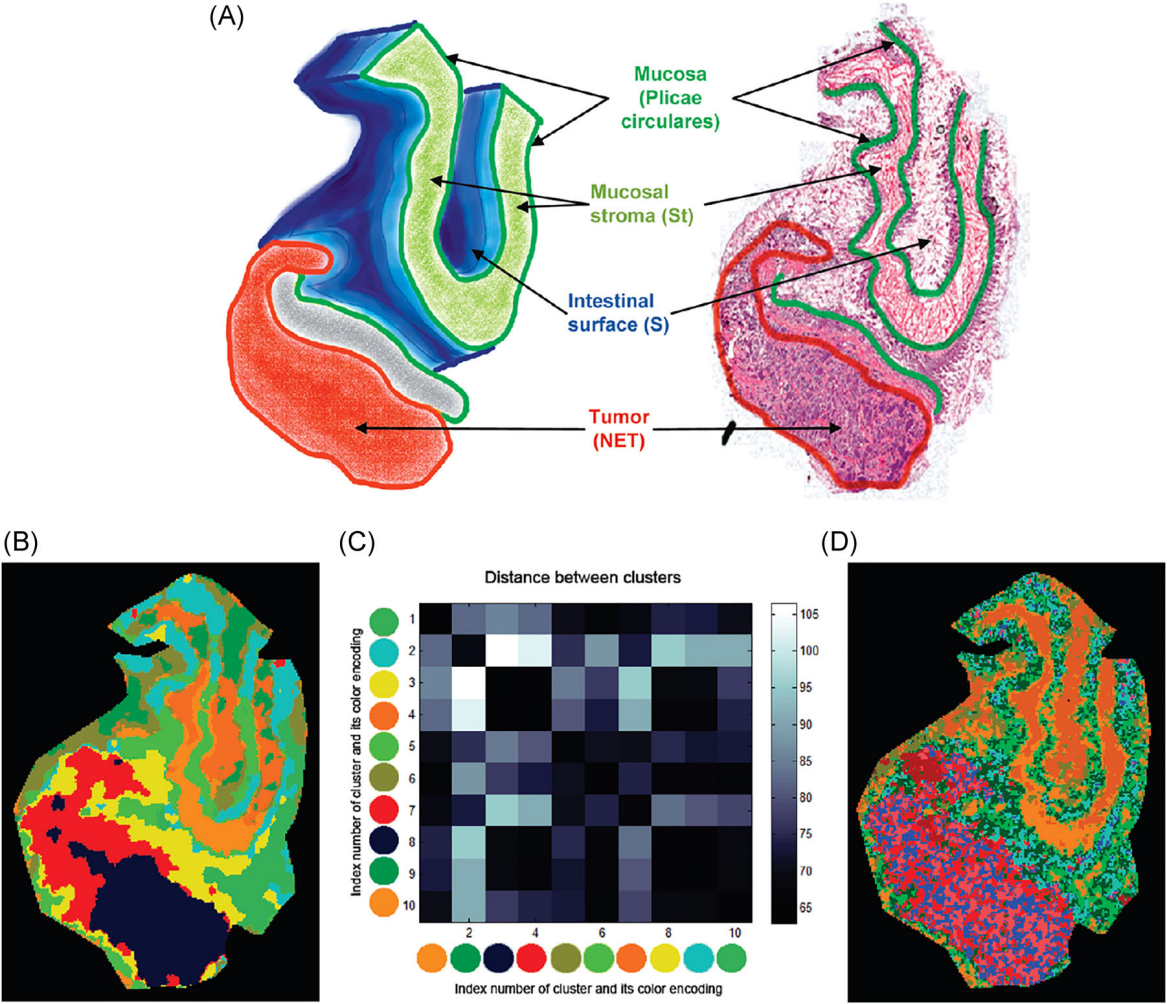
Example of incorporation of spatial information in clustering of MALDI data. Original caption: *The human neuroendocrine tumor dataset. (**a**) 3D‐structure of the tissue used for MALDI‐imaging measurement and optical image of the H&E stained section with main functional structures. (**b**) Segmentation map, strong denoising, 10 clusters. (**c**) The matrix showing distances between clusters for panel B. (**d**) Segmentation map, weak denoising, 10 clusters*. Source: Reprinted with permission from Alexandrov et al., 2010, Figure 10. Copyright 2010 American Chemical Society. MALDI, matrix‐assisted laser desorption/ionization. [Color figure can be viewed at wileyonlinelibrary.com]

One approach is to reduce inter‐pixel variation in IMS data prior to applying the clustering algorithm. McDonnell et al. (2008) demonstrated the use of a basic near‐neighborhood smoothing algorithm on individual ion images, to reduce pixel‐to‐pixel variation in the context of correlation of ion images. While useful for enhancing interpretability of images, a spatial filter such as the median or convolution filter holds the risk of smoothing out the edges between regions and eroding fine structure in the images, which can be an important loss in the context of clustering. Alexandrov et al. (2010) proposed the application of an adaptive, edge‐preserving denoising algorithm. It employs the total variation‐minimizing Chambolle algorithm (Chambolle, 2004), which takes information of neighboring pixels into account and adapts the smoothing to local noise levels, prior to performing HDDC. As shown in Figure 13, the clustering results obtained this way are more spatially homogeneous than those obtained without denoising, and showed consistency with anatomical areas in the tissue. More recently, this strategy was used for data mining of 3D IMS datasets (Trede et al., 2012b; Thiele et al., 2014), where edge‐preserving denoising was applied in three dimensions. The more memory‐efficient bisecting k‐means algorithm was used instead of HDDC in order to cope with the large amounts of data in a 3D IMS dataset. This workflow has also been successfully applied in the clustering of confocal Raman micro‐spectroscopic imaging data (Alexandrov & Lasch, 2013).

Winderbaum et al. (2015) developed a smoothing algorithm that aims to remove spatially dispersed pixels, while smoothing over pixels that show ample regional abundance. The algorithm iterates, using the proportion of neighborhood pixels that exhibit a certain ion to discriminate between noisy pixels and true signal. The resulting smoothed data was used as input for k‐means clustering. Afterward the cluster results were analyzed using a difference in proportions of occurrence (DIPPS) approach, which aims to find the best discriminating m/z bins in the dataset by ranking them for specificity and sensitivity in predicting pixel membership to a cluster.

In other work, Alexandrov et al. (2011) proposed a spatially aware clustering approach, which incorporates spatial information directly into the clustering process rather than through a prefiltering step. The spectral similarity of pixels is combined with their spatial proximity in the tissue (through the use of Gaussian weights) into the distance measure, and clustering is performed on this combined distance matrix.

Alexandrov et al. attempted to assess clustering results using the silhouette criterion (Rousseeuw, 1987). However, no correspondence could be found between the criterion and the visual quality of the maps. The structure‐adaptive version of this algorithm allows the introduction of an additional factor that takes edge‐preservation into account. Furthermore, the FastMap (Faloutsos & Lin, 1995) transformation is introduced for dimensionality reduction, and makes the calculation of the distances between pixels more efficient. The k‐means method, and later bisecting k‐means (Trede et al., 2012b; Klein et al., 2014; Thiele et al., 2014; Krasny et al., 2015), have been used to cluster the data after the dimensionality reduction step. When comparing the newly developed methods to HC combined with PCA, the spatially aware clustering approaches produced smoother, more homogeneous segmentation maps. The spatially aware approaches offered comparable results to the previously used combined edge‐preserving denoising HDDC approach, but with greater speed and memory efficiency.

Hanselmann et al. (2009a) used a posthoc approach to achieve smoother segmentation maps, by applying a median or Markov Random Field (MRF) filter on class‐probability maps generated by a (supervised) random forest‐based classifier. In other work, Hanselmann et al. (2009b) introduced spatial information (also posthoc) into the segmentation of such probability maps using a custom developed multivariate watershed segmentation approach. Chernyavsky et al. (2012) applied posthoc smoothing to the results of latent Dirichlet allocation‐based clustering. It should be noted though that this smoothing is performed on soft segmentation probability maps (discussed below) rather than on hard clustering results. Overall, performing smoothing after clustering does hold the risk of information loss, and integrating spatial information at an earlier level into the analysis is probably preferable. In a different application, Trede et al. (Alexandrov et al., 2011; Trede et al., 2012c) used a topology‐preserving method to increase the spatial resolution of segmentation maps posthoc, creating a super‐resolution map with a spatial resolution finer than that of the original image.

While the integration of spatial information can be valuable for improving the signal‐to‐noise ratio, for removing spurious signals, and for smoothing segmentation results to aid visual interpretation, it must be noted that these methods should be applied with caution. Similar to what was mentioned in Section II.D regarding MAF, these techniques can have adverse effects in datasets where spatial resolution is critical to understanding the underlying biology (or sample content), particularly when the tissue features of interest are only sparsely sampled by the given IMS pixel size. A spatial model that assumes that signals in isolated pixels or small regions are likely due to noise, can potentially filter out small but genuine tissue signals, an aspect mentioned for example in Bruand et al. (2011a). It is therefore important to always consider the underlying assumptions of the model, and consider whether these assumptions are valid for the application at hand.

### F Soft Segmentation Techniques

Much of the work in clustering and digital staining of IMS data uses “hard segmentation,” which means that pixels can only be appointed membership to a single cluster. While hard segmentation can offer valuable and immediate insight into the content of an IMS dataset, there are some caveats to consider. Clustering algorithms such as standard k‐means will in general appoint each pixel to one of k clusters, namely the cluster that has a mean spectrum closest to the spectrum of that pixel. This assignment is done regardless of whether that pixel is a genuinely good match to the cluster mean, or whether that cluster mean is just the best out of a set of not‐so‐good matches. It also means that the pixel does not necessarily resemble the rest of the pixels assigned to that cluster. Besides the fact that cluster quality is often hard to verify in general (also in IMS), the hard assignment of a pixel to one and not several clusters hides a lot of the nuance of how well a pixel fits into its assigned cluster. It is therefore possible that a clustering run groups a set of pixels together because they are loosely similar to each other according to the distance measure, while in reality they are representative of very different tissue classes. That type of situation will be difficult to spot in hard segmentation results.

Another issue poses itself when a pixel exhibits a chemical expression that is a mix of (the means of) two clusters, for example, because the pixel is located at the boundary between two regions. A hard segmentation approach will appoint the pixel to either one region or the other, effectively ignoring the (partial) membership to the region that was not assigned. This becomes especially important in the context of digital staining and border delineation of cancerous tissue, that is, for pixels that lie on the border between different areas of a tumor, or between healthy and diseased tissue.

One way to partly alleviate these problems is to use soft segmentation rather than hard segmentation when performing clustering analysis. In soft segmentation, a pixel can be a member of multiple clusters, rather than being exclusive to a single cluster. This can provide a more nuanced and informed view on the data. However, it has the disadvantage that interpretation becomes harder, as this information can generally no longer be summarized in a single segmentation image. Instead, a pixel‐wise degree of membership or probability map to a particular cluster can be shown as an image.

#### 1 Fuzzy c‐means clustering

**Figure 14:**
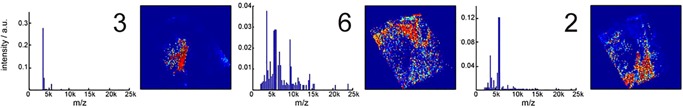
Example of fuzzy clustering. Original caption: *Identification of intratumor heterogeneity in imaging MS datasets by unsupervised multivariate analysis. Target images were created that contain the unrefined heterogeneity in an imaging MS dataset of intermediate grade myxofibrosarcoma. The outputs of principal component analysis, non‐negative matrix factorization, maximum autocorrelation factor analysis, fuzzy* c‐*means, and probabilistic latent semantic analysis were then examined to identify the components that contained the heterogeneity of the target images. The digit contained in the upper right corner of the component mass spectra indicates which component was used. Most data analysis techniques could reproduce the target images. When the component images reproduced the target images it can be seen that the component mass spectra contain the same peptide and protein ions. Note: PCA and MAF can have negative values, consequently the background surrounding the tissue (defined as zero intensity) can change color. y axis labels, a.u., arbitrary units*. Source: Adapted from Jones et al., 2011, Figure 7, under CC‐BY License. MS, mass spectrometry. [Color figure can be viewed at wileyonlinelibrary.com]

A straightforward means of performing soft segmentation is through the fuzzy c‐means clustering (FCM) algorithm. It is similar in its operation to regular k‐means clustering, except that it allows for membership of the data points to multiple clusters. The degree of membership of a data point to a cluster is inversely related to its distance to the various cluster means, with membership summing up to one over all clusters. The degree of “fuzziness,” that is, the degree to which multimembership is allowed, is a hyper‐parameter of the algorithm that needs to be set. Similar to k‐means, the initialization of the algorithm is random, and thus it is preferable to perform multiple iterations of the clustering process. An early application of FCM in 3D SIMS imaging data can be found in Wolkenstein et al. (1999) where wavelet denoising was used to smoothen ion images prior to segmentation. Segmentation was performed on data of individual ion species rather than the full IMS data, with the aim of performing 3D segmentation. In Jones et al. (2011), FCM demonstrated good assessment of tumor heterogeneity in myxofibrosarcoma, providing results comparable with pLSA and NMF. Figure 14 shows an example.

#### 2 AMASS

**Figure 15:**
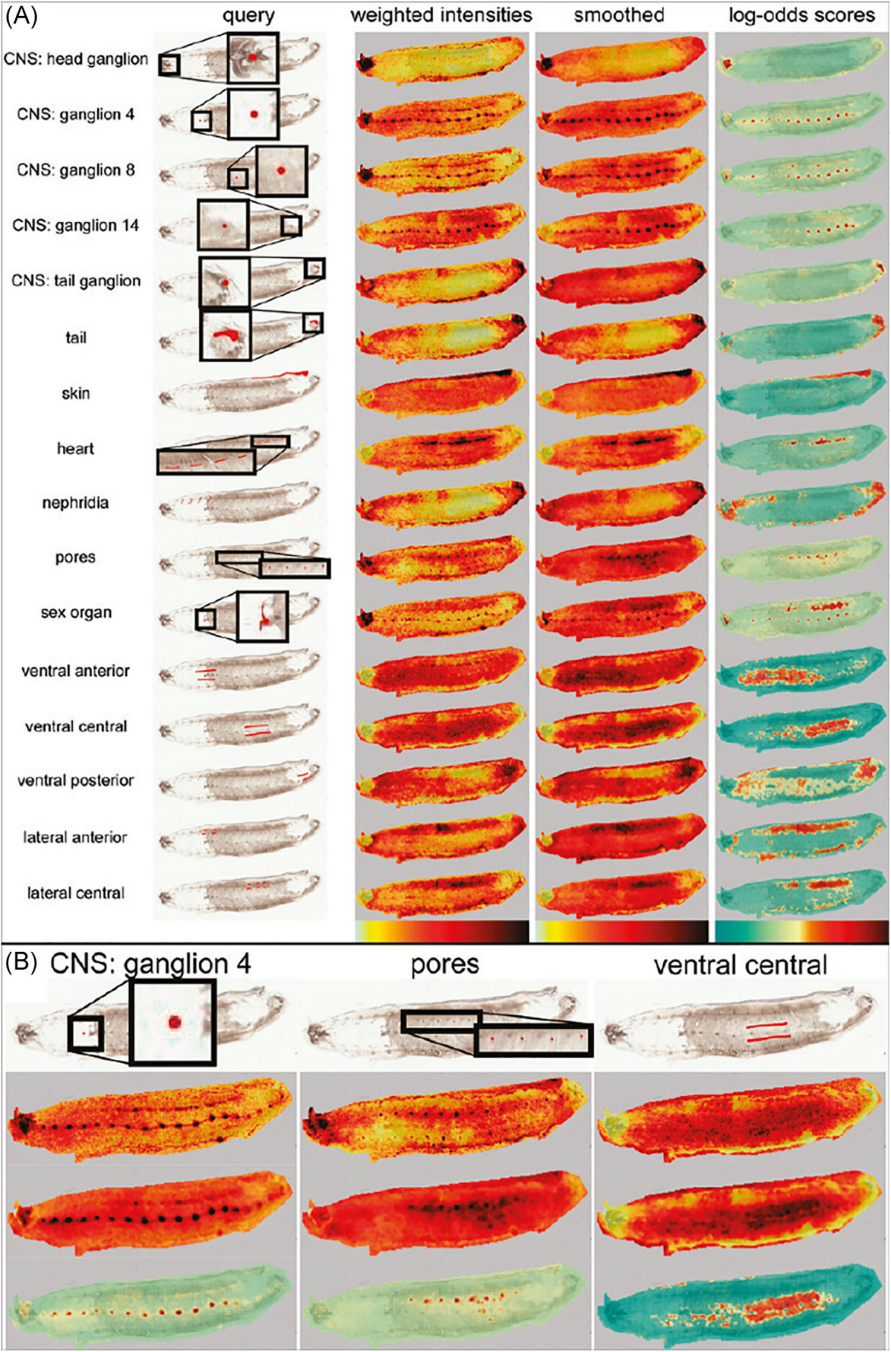
Example of AMASS. Original caption: *(**a**) List of queries and their associated results. Shown on each row are the original query, the corresponding weighted intensities image, the smoothed weighted intensities image, and the log‐odds scores image. Querying with specific image segments results in the recruitment of other spots with similar molecular signatures. For example, querying with one ganglion or a few pores recruits the whole CNS or the rest of the pores, respectively. (**b**) Detailed images for 3 different queries. We can see that while smoothing helps in cleaning noise on larger queries such as the ventral query, it also can cause the loss of some MALDI spots in the case of smaller regions, such as the pores*. Source: Reprinted with permission from Bruand et al., 2011a, Figure 2. Copyright 2011 American Chemical Society. AMASS, algorithm for MSI analysis by semisupervised segmentation. [Color figure can be viewed at wileyonlinelibrary.com]

Bruand et al. (2011a) developed the AMASS algorithm, which allows for soft‐segmentation of an IMS dataset. While this algorithm was developed for semisupervised segmentation, based on an initial segmentation seed provided by user input (AMASS stands for algorithm for MSI analysis by semisupervised segmentation), it also provides good segmentation results when used in an unsupervised way, through random initialization. AMASS uses a probability‐based approach to clustering. After random initialization, the algorithm first ranks the importance of each m/z value in discriminating between clusters using the Mann‐Whitney‐Wilcoxon ρ‐statistic Winderbaum et al. (2015) use a similar approach). Since this is a rank‐based statistic, it prevents single m/z bins from having too much influence on the clustering process. The resulting weighted intensity scores are then used to calculate the log‐odds scores for each individual pixel to belong to each of the clusters. In the next iteration, the algorithm looks for pixels with similar scores against all clusters, and attempts to define new clusters based on these similarities, repeating the process until convergence. Pixels are not required to belong to any of the clusters, if no proper match is found. The final clustering results can be used as soft segmentation maps, where the log‐odds scores show the probability of a pixel belonging to each of the clusters. Furthermore, the log‐odds scores can be used in a further HC step if hard segmentation is desired. An example of the AMASS algorithm is shown in Figure 15.

#### 3 Latent Dirichlet Allocation

Chernyavsky et al. (2012) used latent Dirichlet allocation in the soft segmentation of MALDI IMS data. Latent Dirichlet allocation is a generative statistical model that was developed in the context of natural language text processing, and is closely related to the pLSA method used for factorization by Hanselmann et al. (2008) (discussed above). Latent Dirichlet allocation can be seen as a generalization of pLSA that provides a more advanced generative model, as it allows modeling of topic distributions per document through Dirichlet priors (Blei, Ng, & Jordan, 2003). In the context of clustering IMS data, the latent Dirichlet allocation model gives the distribution of each tissue type (topic, cluster) per pixel (document), and can thus assign probabilities for each pixel to belong to a cluster. Furthermore, the model provides a mass distribution for each topic, which is equivalent to the cluster mean in other methods. Chernyavsky et al. note that latent Dirichlet allocation has reduced memory requirements over HC, and reduced complexity compared to HDDC. They also note that the method incorporates more information into the clustering process than most other clustering methods that incorporate only distance. Chernyavsky et al. (2012) use the method in the segmentation of MALDI IMS data acquired from mouse brain tissue, demonstrating the combination of multiple probability maps. Furthermore, the method is combined with posthoc smoothing (using a Markov Random Field filter on the probability maps) to generate smooth segmentation maps, similar to Hanselmann et al. (2009a). Willse et al. (2002) have proposed the use of Poisson and multinomial mixture models for the segmentation of SIMS imaging data, where model parameters are estimated using an EM approach, and each pixel is assigned a probability of belonging to each of k mixture classes.

#### 4 Spatial Shrunken Centroids

Bemis et al. (2016) developed the spatial shrunken centroids framework, a statistical model‐based framework for classification and segmentation of IMS data (incorporated into the Cardinal R package by the same authors). The spatial shrunken centroid framework combines the concept of nearest shrunken centroid classification (proposed by Tibshirani et al. (2002, 2003) for the classification of gene expression microarray data) with that of spatially aware clustering by Alexandrov et al. (Alexandrov et al., 2010; Alexandrov & Kobarg, 2011). The nearest shrunken centroid method builds on standard nearest centroid classification by adding feature selection directly into the classification step. Standard nearest centroid classification is the classification step that occurs in each iteration of the k‐means clustering algorithm, whereby a pixel is compared to the centroid of each class by calculating the distance between the pixel and the cluster centroid. The pixel is then assigned to, or classified into, the cluster whose centroid lies closest. In standard nearest centroid classification, all features, that is, the full (or peak‐picked) spectrum is used to calculate this distance, whereas the nearest shrunken centroid method aims to use only those features for classification that are relevant to the cluster class. This feature selection process is achieved through a combination of within‐class feature normalization, *t*‐statistics, and soft‐thresholding. This makes the centroids easy to interpret, and gives direct insight into which spectral features are truly relevant for the cluster, while removing noisy features that do not contribute to the clustering process. We refer to Tibshirani et al. (2003) and Bemis et al. (2016) for details.

**Figure 16:**
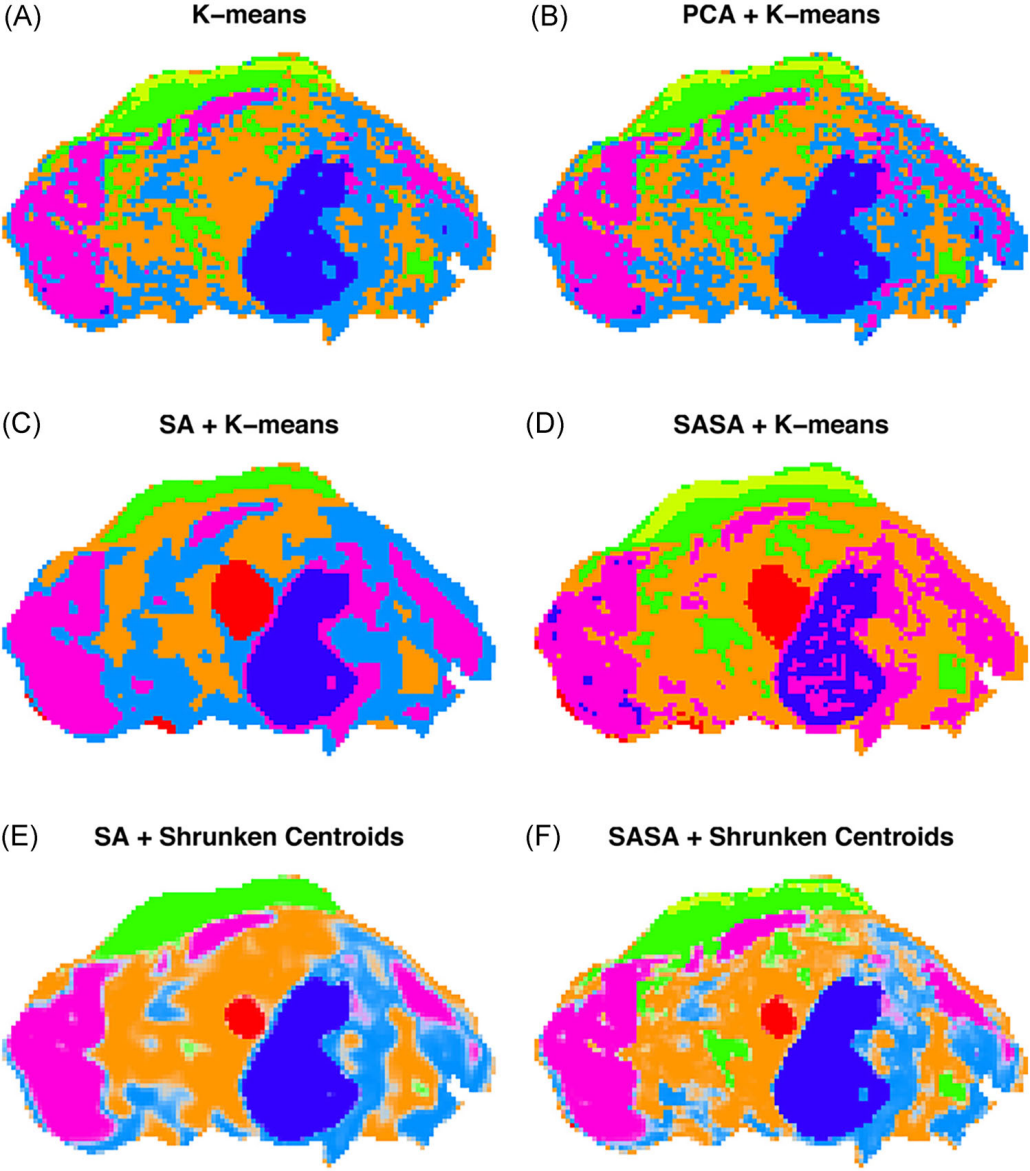
Example of spatial shrunken centroids clustering. Original caption: *Pig fetus cross‐section: segmentation comparison. (**a**)* k *‐means clustering applied to the peak‐picked spectra. (**b**)* k‐*means clustering applied to the first five principal components of the peak‐picked spectra. (**c**) Spatially aware (SA) clustering. (**d**) Spatially aware structurally adaptive (SASA) clustering. (**e**) Spatial shrunken centroids with SA distance. (**f**) Spatial shrunken centroids with SASA distance*. Source: Reproduced from Bemis et al., 2016, Figure 3, under CC‐BY License. [Color figure can be viewed at wileyonlinelibrary.com]

Bemis et al. (2016) have applied the spatial shrunken centroids framework to several datasets, including a DESI IMS dataset obtained from a pig fetus cross‐section and MALDI IMS and DESI IMS datasets from rat brain sections. In the pig fetus dataset, the spatial shrunken centroids method showed spatially smooth clustering results, with little noise and clean edges between segments. An example is shown in Figure 16. The result of the segmentation process is a probability per pixel of membership to each cluster, where the number of clusters is determined in a data‐driven way by observing the interactions between the segmentation results and the shrinkage factor. The method robustly retrieves the same clusters with different types of spatial smoothing incorporated into the analysis, outperforming regular k‐means clustering. The shrunken centroids also allow for easy selection of important and representative ion images for each cluster. Patterson et al. (2016) have used the spatially‐aware shrunken centroids method in the analysis of 3D MALDI IMS data of atherosclerotic plaque collected in human and mouse tissue.

### G Correlation Analysis

A spatial segmentation of an IMS dataset will divide the tissue or sample into spatial regions with similar chemical content. It is generally possible to examine the mean chemical content of a cluster by, for example, taking the mean or median mass spectrum over all pixels in that cluster (i.e., the cluster centroid). However, the segmentation usually does not provide direct information on which ions are specifically or differentially expressed in the spatial region highlighted by that cluster. To retrieve such information, the area defined by the cluster can be used as a mask to perform a query for ion images that have a spatially similar or correlating expression. Although in many cases these techniques can be considered supervised (in that a user provides the target distribution to correlate ion images to), their relationship to clustering of spatial distributions merits a quick overview.

Van de Plas et al. (2007c) developed a non‐negative least squares approach that allowed filtering and retrieval of ion images on the basis of their spatial similarity to a (binary) spatial query image (e.g., a user‐drawn or clustering‐provided ROI). This approach allowed for fast retrieval of relevant ion images, and included more advanced filter criteria such as “don't care pixels.” Alexandrov et al., 2010) used binary masks defined through clustering to find correlating ion images in a full IMS dataset. Suits et al. (Suits et al., 2013; Fehniger et al., 2014) developed and demonstrated an efficient approach, using (spatial) correlation, to find ion images that have a similar spatial expression to a target ion image or mask image. McDonnell et al. (2008) investigated colocalization between ion images on the basis of (spatial) correlation. Kaddi et al. (2013) used a hypergeometric similarity measure to retrieve ion images similar to a query ion image, while Bruand et al. (2011a, 2011b) showed the use of clustering results and ROIs to find molecular signatures and ions of interest, using rank‐based statistics.

In related research, Verbeeck et al. (2014a) demonstrated the use of masks (defined through linking IMS data to an anatomical atlas) to retrieve ions specific to a particular anatomical structure. Similar atlas‐based approaches can be found in Abdelmoula et al. (2014a) and Carreira et al. (2015). Besides providing a more in‐depth analysis of clustering results, spatial correlation querying can also be used to get deeper insights into the results of matrix decompositions. The peaks provided by the pseudospectra in a matrix decomposition are not always directly interpretable. By using the spatial expression image of a factorization component as the input to a spatial query, or by creating a binary mask on the basis of a component image using thresholds, it becomes possible to quickly retrieve ion images related to a particular component in the IMS data.

## IV MANIFOLD LEARNING

Techniques such as PCA, ICA, and NMF are often used to project high‐dimensional IMS data to a lower‐dimensional space. Representing a dataset using a reduced number of variables, while minimizing information loss, tends to reveal underlying structure and trends in the data. While factorization techniques are often useful for this purpose, their linear nature makes them less‐suited if nonlinear structure is present in the data. In that case, to enable integration of nonlinearities into the dimensionality reduction, (nonlinear) manifold learning techniques (Cayton, 2005; Tenenbaum, De Silva, & Langford, 2000; Roweis & Saul, 2000) can be employed.

**Figure 17:**
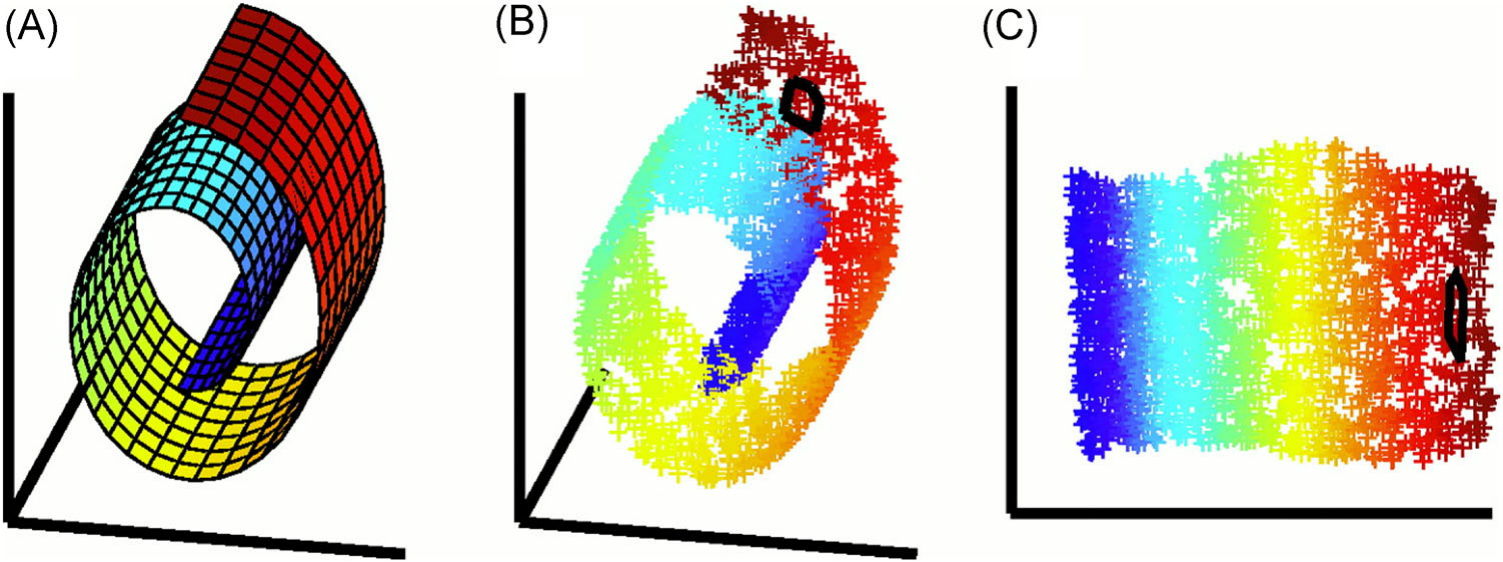
Example of nonlinear dimensionality reduction. Original caption: *The problem of nonlinear dimensionality reduction, as illustrated for three‐dimensional data (**b**) sampled from a two‐dimensional manifold (**a**). An unsupervised learning algorithm must discover the global internal coordinates of the manifold without signals that explicitly indicate how the data should be embedded in two dimensions. The color coding illustrates the neighborhood preserving mapping discovered by LLE; black outlines in (**b**) and (**c**) show the neighborhood of a single point. Unlike LLE, projections of the data by principal component analysis (PCA) or classical MDS map faraway data points to nearby points in the plane, failing to identify the underlying structure of the manifold. Note that mixture models for local dimensionality reduction, which cluster the data and perform PCA within each cluster, do not address the problem considered here: namely, how to map high‐dimensional data into a single global coordinate system of lower dimensionality*. Source: Roweis and Saul, 2000, Figure 1. Reprinted with permission from AAAS. [Color figure can be viewed at wileyonlinelibrary.com]

The idea behind manifold learning is that the dimensionality of the data are only artificially high, and that the data points in reality lie on a lower‐dimensional manifold that is embedded in the high‐dimensional feature space (Cayton, 2005). A manifold is defined as a topological space, which is locally Euclidean. One well‐known example of a manifold is a surface, which is a two‐dimensional manifold. The goal of nonlinear manifold learning is to uncover this underlying manifold structure from the data, and in that sense it can be viewed as a nonlinear partner to the previously discussed linear factorization methods. If the data populates a linear subspace (e.g., a flat plane rather than a curved surface), linear techniques such as PCA can reveal that subspace exactly. If it populates a nonlinear subspace, manifold learning will be more naturally suited to capture that subspace efficiently. An example of this is shown in Figure 17. Nonlinear dimensionality reduction techniques have been shown especially useful for visualization of high‐dimensional data. Easy visualization and human interpretation are largely limited to 3D or 4D representations. Higher‐dimensional visualizations quickly become overwhelming (de Oliveira & Levkowitz, 2003). Since manifold learning is often able to pack more data variation into a lower‐dimensional representation than a linear technique, it is often better suited to provide a visual representation of data without leading to overly complex plots.

In the case of IMS data, linear techniques generally require more dimensions to graphically represent the bulk of the information than manifold learning techniques, which tend to provide a richer and more concise representation of the high‐dimensional measurements.

Furthermore, where most linear techniques (such as PCA) focus on keeping dissimilar points far apart in the low‐dimensional representation, manifold learning techniques such as t‐distributed stochastic neighborhood embedding (t‐SNE) focus on representing similar data points close together in the target space. This is sometimes more informative (van der Maaten & Hinton, 2008) and leads to clustering of similar points regardless of whether their relationship is linear. Manifold learning techniques have been used for dimensionality reduction in applications where nonlinearities play a substantial role, such as the classification of written data (van der Maaten & Hinton, 2008; Roweis & Saul, 2000), facial recognition (Tenenbaum, De Silva, & Langford, 2000; Roweis & Saul, 2000), and recognition of objects photographed from different angles (Tenenbaum, De Silva, & Langford, 2000). A disadvantage of manifold learning techniques, compared to linear factorization, is that it is often difficult to explicitly determine the new dimensions or variables that constitute the projections, whereas in linear factorization methods these arise naturally as products of the decomposition.

### A t‐Distributed Stochastic Neighborhood Embedding

#### 1 Principle

While a host of manifold learning techniques exist (Cayton, 2005), t‐SNE, a technique introduced by van der Maaten and Hinton, 2008), has seen particular application to MALDI IMS data. The t‐SNE algorithm has two steps. First, it converts the Euclidean distances between pairs of data points in the high‐dimensional space into conditional probabilities, where similar points have a high probability of being picked as neighbor and dissimilar points have a very low probability of being picked as neighbor. In the second step, the algorithm takes this probability distribution in the high‐dimensional space and arranges the points in the low‐dimensional space such that their arrangement results in a probability distribution that matches that of the points in the high‐dimensional space as closely as possible. This is achieved by minimizing the Kullback‐Leibler divergence (a statistical measure for comparing probability distributions) between the high and low‐dimensional probability distributions.

#### 2 Application to IMS

**Figure 18:**
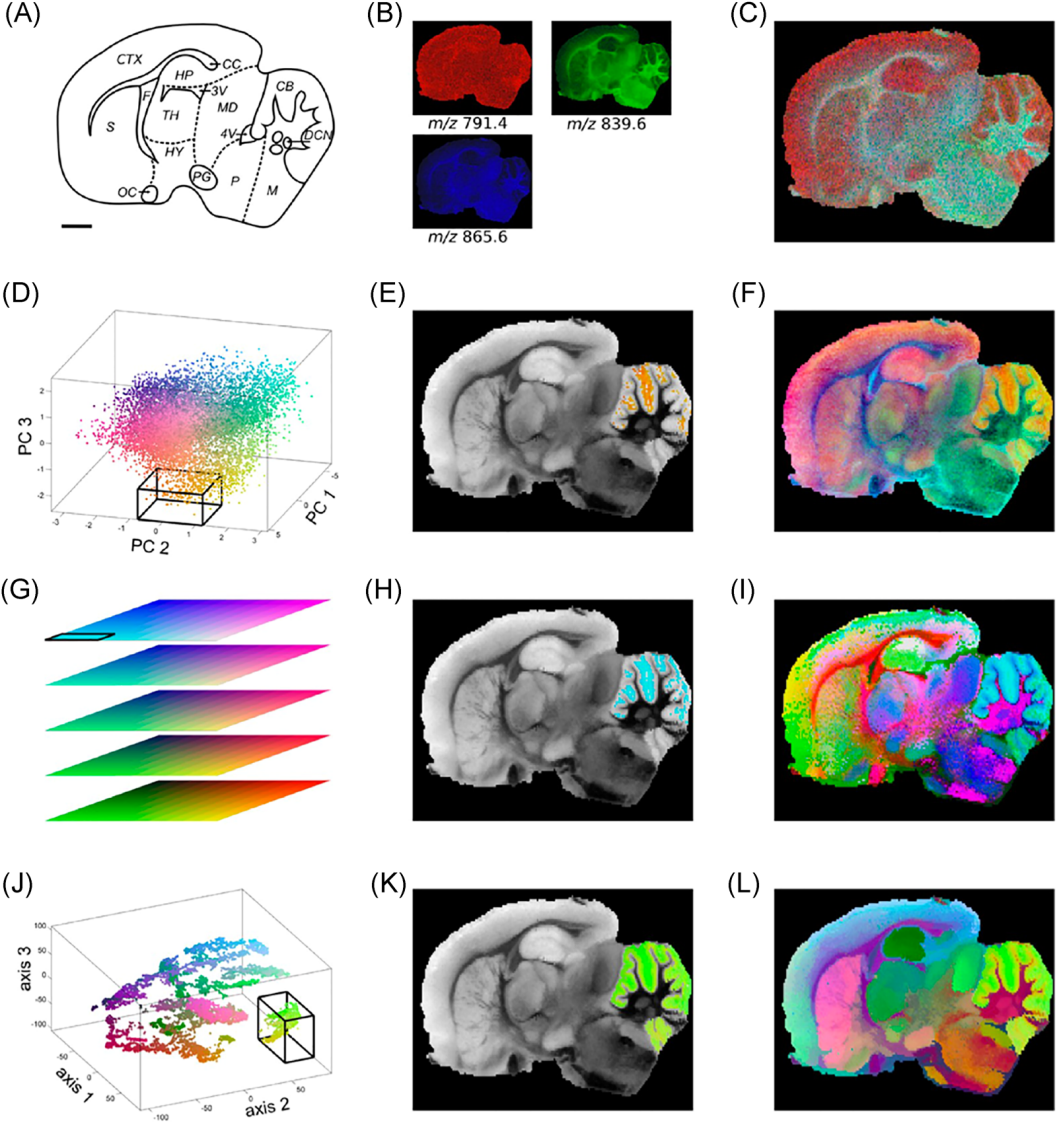
Example of t‐SNE applied to MALDI IMS data. Original caption: *RGB color‐coding of hyperspectral modeling results. (**a**) Schematic of the anatomy for the rat brain that was subjected to MALDI MSI after formalin fixation; scale bar = 2 mm. CB, cerebellum; CC, corpus callosum; CTX, cerebral cortex; DCN, deep cerebellar nuclei; F, fornix; HP, hippocampus; HY, hypothalamus; M, medulla; MD, midbrain; OC, optic chiasm; P, pons; PG, pituitary gland; S, septum; TH, thalamus; 3V, third ventricle; 4V, fourth ventricle. (**b**) Three randomly chosen single m/z images (m/z791.4, 839.6, and 865.6). (**c**) An overlay of the three images in b is shown, through combining the individual red, green, and blue intensities for each pixel as additive colors (white pixels consist of high levels of red, green, and blue). (**d**) PCA space: the location of a pixel (each pixel is represented by a dot) on principal component 1 (PC 1), PC 2 and PC 3 determines the intensity for red, green and blue (RGB), respectively. (**e**) The pixels contained in the box in the PCA scores plot in d are shown in color. (**f**) The image after color‐coding the pixels with the RGB‐scheme shown in d. (**g**) SOM space: a unique color for each SOM unit is assigned with red, green, and blue representing the location along the three dimensions of the 3D SOM map (20 × 10 × 5). (**h**) The pixels that were mapped in the square 3 × 3 × 1 section of the SOMmap highlighted in g can be seen in the original image with the same color‐coding. (**i**) The complete image with SOM‐based RGB color‐coding. (**j**) t‐SNE space: the scatter plot of pixels in the t‐SNE model shows clear clustering patterns, and pixels are RGB color‐coded based on their positions on the three axes. (**k**) The cluster selected with the box in j is shown as colored pixels in the image. (**l**) The image after color‐coding the pixels with RGB values determined by the t‐SNE manifold learning method*. Source: Reprinted with permission from Fonville et al., 2013, Figure 2. Copyright 2013 American Chemical Society. IMS, imaging mass spectrometry; MALDI, matrix‐assisted laser desorption/ionization; t‐SNE, t‐distributed stochastic neighborhood embedding. [Color figure can be viewed at wileyonlinelibrary.com]

In IMS research, t‐SNE was first applied by Fonville et al. (2013) to devise a novel, intuitive visualization technique for MALDI IMS data, and hyperspectral data in general. The goal of this visualization technique is to represent as much information from the data as possible in a single overview image, similar to what standard (hard) segmentation maps aim to do. Rather than clustering the data, however, Fonville et al. combined t‐SNE dimensionality reduction with a color‐coding scheme. The color scheme encodes the distances (in the dimensionality‐reduced IMS data) between pixels. In this way, the method allows for visualization of the continuum of slightly differing molecular profiles between pixels. Figure 18 shows an example of this. In other work, the same group investigated color coding schemes more deeply, and addressed their impact on human perception (Race & Bunch, 2015). Fonville et al. compared the performance of t‐SNE to that of PCA and nonlinear dimensionality reduction techniques such as self‐organizing maps (discussed below), Isomap, Sammon's mapping, and kernel PCA. Out of these methods, t‐SNE came forward as best‐suited for the color‐coding approach. The t‐SNE algorithm did incur a significant computational burden with relatively long processing times. However, optimization of the t‐SNE algorithm is ongoing (van der Maaten, 2014) and this is expected to improve in the future. Abdelmoula et al. (2014b) have used this visualization in the generic spatial registration of IMS data to histological images. This same workflow was used for the registration of SIMS imaging data to histology in Škrášková et al. (2015). Furthermore, Abdelmoula et al. (2016) used t‐SNE, combined with bisecting k‐means, to study tumor heterogeneity and subpopulations in gastric and breast cancer. They used this setup to determine the number of subpopulations and assess their clinical significance. Abdelmoula et al. (2018) have also used hierarchical stochastic neighbor embedding (HSNE) in the clustering of 3D MALDI IMS data. HSNE is a scalable version of the t‐SNE that is better suited to deal with large datasets than standard t‐SNE, and, as its name suggests, operates in a hierarchical manner, first calculating a coarse representation, after which finer representations can be calculated for ROIs. Meanwhile, Inglese et al. (2017) used parametric t‐SNE in the analysis of 3D DESI IMS data from a human colorectal adenocarcinoma biopsy. In parametric t‐SNE, the nonlinear mapping between the original pixel data and the low‐dimensional space is parameterized by means of a (deep) autoencoder neural network. Deep neural networks (Bengio, 2009) are multilayered artificial neural networks (see also Section IV.B) that have shown to be very powerful in a wide number of applications, including biomedical imaging (Shen, Wu, & Suk, 2017), due to their ability to model complex nonlinear relationships. The use of parametric t‐SNE has two important advantages over standard t‐SNE, namely that (i) new samples can easily be projected to an existing t‐SNE mapping, and (ii) the t‐SNE modeling results are more consistent across different runs. After mapping the pixel data to the low‐dimensional space, Inglese et al. used the OPTICS (Ankerst et al., 1999) density‐based clustering algorithm to segment each of the individual (2D) tissue slices in the 3D dataset. This workflow allowed the group to identify clusters in the tissue that were not visible using linear dimensionality reduction methods, and which could be linked to high expressions of key metabolites in colon cancer. Related to this methodology, Thomas et al. (2016) used a neural network autoencoder approach in the dimensionality reduction of MALDI IMS data of transversal sections of mouse brain. In recent work, Smets et al. (2019) have applied Uniform Manifold Approximation and Projection (UMAP), a novel manifold learning method, which is highly suited for the analysis of large and high‐dimensional datasets. Compared to t‐SNE, UMAP showed embedding results of at least the same quality, while greatly reducing runtime and providing improved scalability.

### B Self‐organizing Maps

#### 1 Principle

Self‐organizing maps (SOMs), also called Kohonen (neural) networks (Kohonen, 1998), are a second manifold learning technique that has been applied on multiple occasions in IMS studies. Kohonen developed the SOM as a tool to visualize and analyze high‐dimensional data. A SOM is a type of artificial neural network that aims to organize information spatially, namely by mapping similar concepts to adjacent areas in the neural network. This was inspired by how some types of information are believed to be stored in the human brain. For example, pitches of tones are thought to be mapped to spatial distance in the auditory cortex, while the somatotopic map is thought to map skin surface to spatial locations in the cortex. A SOM consists of a single layer neural network, where the neurons are arranged in an n‐dimensional (usually two‐dimensional) rectangular or hexagonal grid. Each neuron is connected to its neighboring neurons, thus representing an n‐dimensional topological map. When employing SOMs for the clustering of pixels in IMS data, the goal is to map the high‐dimensional IMS data to this low‐dimensional grid, while keeping similar pixels together on the topological map. Pixels that are similar in chemical content and thus close together in the high‐dimensional space, remain close together in the low‐dimensional space. Once mapped, each grid point or neuron represents a prototype object, that is characteristic for the pixels closest to it, similar to the mean value of the clusters in k‐means clustering.

#### 2 Application to IMS

Wolkenstein et al. (1997) applied SOMs for the spatial segmentation of solder alloys, allowing for classification of the different chemical phases in the specimens. The network showed good classification of the phases compared to manual annotation. The size of the SOM was tuned through component selection in PCA or through a secondary clustering step of the SOM neurons.

**Figure 19:**
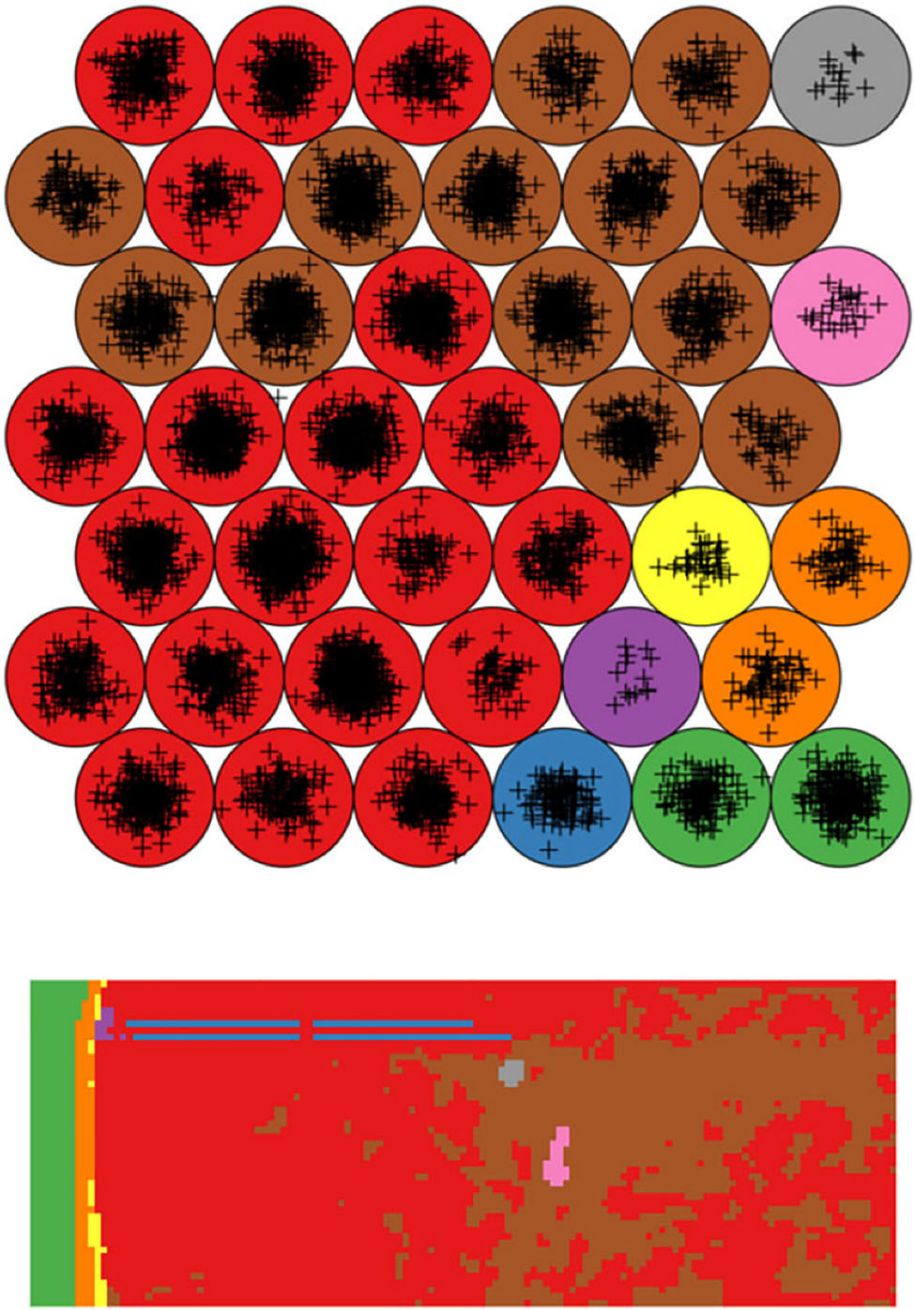
Example of a SOM in MALDI IMS. Original caption: *Results of the SOM mapping of the apple dataset*. 6×7 *The lattice is the 2D representation of the SOM. Each plus is a pixel. The nine different colors highlight the result of the hierarchical clustering on the codebook vectors. Bottom panel: output of the segmentation process. The color of each pixel is selected on the base of the SOM unit it is mapped to*. Source: Franceschi and Wehrens (2014), Figure 2, reproduced with permission from John Wiley & Sons. IMS, imaging mass spectrometry; MALDI, matrix‐assisted laser desorption/ionization; SOM, self‐organizing map. [Color figure can be viewed at wileyonlinelibrary.com]

Franceschi & Wehrens (2014) have used SOMs in the analysis of metabolite‐focused MALDI IMS data collected from slices of apple. In a first application, SOMs were used to perform spatial, pixel‐wise segmentation of the data. A SOM of size 6×7 and the Euclidean distance measure were used to perform the analysis. Each of the 42 gridpoints represents a spatial distribution in the original tissue section, along with a prototype spectral signature. Figure 19 shows the SOM in question. Due to the relatively large number of clusters, HC was performed on the cluster prototypes to further reduce the number of clusters to nine. These clusters were visualized in the tissue, giving areas that overlap with biologically relevant regions. In a second application, the SOMs were used to perform clustering on the ion images, similar to Konicek et al. (2012) and Alexandrov et al. (2013b). This approach resulted in a map where each neuron represents a prototype ion image for all the ion images associated with it. One advantage of SOMs is that it is straightforward to read from the resulting map (on the basis of proximity) which prototype ion images have a similar spatial distribution. The clustering allowed for a quick overview of the contents of the IMS dataset, and enabled highlighting of colocalized metabolites in the sample. Xiong et al. (2012a) have used self‐organizing maps in the segmentation of DESI imaging data, with the goal of identifying tumorous and nontumorous regions in human bladder tissue. Learning vector quantization, a supervised classification algorithm related to SOMs, was used to build a model for classifying new tissues on the basis of a training segmentation. Wijetunge et al. (2014) have applied Growing self‐organizing maps (GSOMs), a variant of SOMs that does not require the map size to be defined a priori. The map can start with a specified minimum number of nodes, and adds new nodes when necessary, side‐stepping one of the main challenges of classic SOMs. Similar to Franceschi & Wehrens (2014), the GSOM was applied to cluster similar ion images together, grouping the original 250 (peak‐picked) ion images into 62 relevant clusters. Furthermore, through color‐coding the applied GSOM algorithm allowed for easy discovery of highly similar grid points. Xiong et al. (2012c) have used SOMs for the spatial registration of IMS data collected in different tissue sections in order to construct a 3D IMS dataset.

## V SOFTWARE PACKAGES AND DATA ANALYSIS TOOLS

This final section enumerates several free software packages that allow the reader to get started with the computational analysis of IMS data. Many of these packages implement several of the techniques and algorithms that have been discussed in this review.

- Datacube Explorer enables fast and easy visualization and basic analysis of large IMS datasets, without requiring programming by the user (Smith et al., 2012; Klinkert et al., 2014).
- BioMap is a biomedical image analysis software package by Rausch and Stoeckli, available at http://ms-imaging.org/wp/biomap/.
- Cardinal is an extensive R‐based statistical analysis package for IMS data (Bemis et al., 2015).
- msIQuant is a software package for IMS that enables fast access, visualization, and analysis for large datasets (Källback et al., 2016).
- MSI.R is a data exploration tool for IMS data, enabling the use of contour lines to visualize ion intensities (Gamboa‐Becerra et al., 2015).
- rMSI is an R‐based IMS data handling and analysis tool (Ràfols et al., 2017), available at https://github.com/prafols/rMSI.
- MALDIquant is an R‐based package for the analysis of mass spectrometry data in general, which allows analysis of IMS data as well (Gibb and Strimmer, 2012).
- OpenMSI is a web‐based visualization, analysis, and management platform for IMS data (Rübel et al., 2013; Fischer et al., 2016).
- MSiReader is an open‐source MATLAB‐based tool for analysis and visualization of IMS data (Robichaud et al., 2013).
- SpectralAnalysis is an open‐source MATLAB‐based IMS data analysis toolbox, allowing multivariate analysis of large IMS datasets (Race et al., 2016).
- BASIS is an open‐source bioinformatics platform for processing large‐scale mass spectrometry imaging datasets, developed by Veselkov et al. (2018).
- OmniSpect is an open‐source MATLAB‐based toolbox, developed for the visualization and analysis of IMS data (Parry et al., 2013).
- MCR‐ALS GUI is a MATLAB‐based toolbox that provides MCR‐ALS analysis for various data, and includes a module for the analysis of spectral imaging data (Jaumot et al., 2005; Jaumot, de Juan, & Tauler, 2015).
- NESAC/BIO MVA Toolbox is a MATLAB‐based toolbox developed by Dan Graham for the multivariate analysis of TOF‐SIMS imaging data, available at http://www.nb.engr.washington.edu/mvsa/nbtoolbox.

## VI CONCLUSIONS

Unsupervised data analysis methods have become crucial for extracting valuable insights from the large and high‐dimensional datasets collected through IMS. With advancing instrumental capabilities and sample preparation improvements, the size and dimensionality of IMS measurements is expected to continue to grow, while the advances in speed of acquisition will deliver more datasets than ever before. These advancements, together with an increased adoption of IMS in a growing set of application domains, will require development of even more capable data exploration methods to navigate the vast amounts of IMS data that will become available. With this review, we hope to provide an overview and a stepping stone for the computational community as well as for the mass spectrometry community, to help make those advances in exploratory methods for IMS possible.

**ABBREVIATIONS**
AFAI‐IMS: airflow assisted ionization imaging mass spectrometry
ALS: alternating least squares
AMASS: algorithm for MSI analysis by semisupervised segmentation
BIC: Bayesian information criterion
DA: discriminant analysis
DESI: desorption electrospray ionization
DIPPS: difference in proportions of occurrence
DWT: discrete wavelet transform
EM: expectation‐maximization
FA: factor analysis
FCM: fuzzy *c*‐means clustering
FFPE: formalin fixed paraffin embedded
FTICR: Fourier transform ion cyclotron resonance
GMM: Gaussian mixture model
GPU: graphical processing unit
GSOM: growing self‐organizing maps
HC: hierarchical clustering
HDDC: high dimensional data clustering
HPC: high performance computing
ICA: independent component analysis
IMS: imaging mass spectrometry
LAESI: laser ablation electrospray ionization
LAICP: laser ablation inductively coupled plasma
LDI: laser desorption/ionization
MAF: maximum autocorrelation factorization
MALDI: matrix‐assisted laser desorption/ionization
MCR: multivariate curve resolution
MCR‐ALS: multivariate curve resolution by alternating least squares
MCR‐WALS: multivariate curve resolution by weighted alternating least squares
MDL: minimum description length
MLPCA: maximum likelihood principal component analysis
MNF: minimum noise fraction
MOLDL: molecular dictionary learning
MRF: Markov random field
MS: mass spectrometry
NMF: non‐negative matrix factorization
NMR: nuclear magnetic resonance
NN‐PARAFAC: non‐negativity constrained parallel factor analysis
O‐PLS: orthogonal projection to latent structures
PC: principal component
PCA: principal component analysis
pLSA: probabilistic latent semantic analysis
PMF: positive matrix factorization
RAM: random‐access memory
REIMS: rapid evaporative ionization mass spectrometry
ROI: region of interest
SIMS: secondary ion mass spectrometry
SMCR: self‐modeling curve resolution
SOM: self‐organizing maps
SVD: singular value decomposition
t‐SNE: t‐distributed stochastic neighborhood embedding
TIC: total ion current
TOF: time of flight
